# Supplementary material for: Swiss-wide multicentre evaluation and prediction of core outcomes in arthroscopic rotator cuff repair: protocol for the ARCR_Pred cohort study
Source: BMJ Open. 2021 Apr 22;11(4):e045702. doi: 10.1136/bmjopen-2020-045702 (PMC8070866; doi:10.1136/bmjopen-2020-045702)
Supplement: Supplementary data [file bmjopen-2020-045702supp001.pdf]

Supplement file 1

**Article title**	Protocol for the ARCR_Pred cohort-study: Swiss-wide multicenter evaluation and prediction of core outcomes in arthroscopic rotator cuff repair
**Journal name**	BMJ Open Access
**Author names**	Laurent Audigé, Heiner C. Bucher, Soheila Aghlmandi, Thomas Stojanov, David Schwappach, Sabina Hunziker, Christian Candrian, Gregory Cunningham, Holger Durchholz, Karim Eid, Matthias Flury, Bernhard Jost, Alexandre Lädermann, Beat Moor, Philipp Moroder, Claudio Rosso, Michael Schär, Markus Scheibel, Christoph Spormann, Thomas Suter, Karl Wieser, Matthias A. Zumstein, ARCR_Pred Study Group*, Andreas Müller
**Corresponding author**	Laurent Audigé, Schulthess Klinik, CH-8008 Zurich, Switzerland
**e-mail address**	laurent.audige@kws.ch

* Members of the ARCR_Pred study group are listed in the main publication of this project protocol.

Patient information sheet and informed consent form (English version) for the project site “University Hospital of Basel” (USB)

Universitätsspital Basel

Engl. Surgical safety and effectiveness in orthopaedics: evaluation of an international consensus core set of adverse events in arthroscopic rotator cuff repair

This project has been organised by: PD Dr. Andreas Müller (project leader; Deputy Consultant of Orthopaedics, Shoulder/Elbow team leader, Orthopaedic and Traumatology Department, University Hospital of Basel) and Prof. Dr. Laurent Audigé (project manager; Research Associate, Orthopaedic and Traumatology Department, University Hospital of Basel and Head of Research Group Upper Extremities, Department of Research and Development, Schulthess Clinic, Zurich)

Sponsor: University Hospital of Basel, PD Dr. Andreas Müller, Deputy Consultant of Orthopaedics and Traumatology

Dear Sir / Madam,

We would like to ask you if you are interested in participating in a research project. The planned project is first presented as a short summary in the table provided below followed by a more detailed description.

Summary of the project

1	**Aim of the project**<br>The research project will investigate how easily the most important results are predicted following surgical interventions to treat tears of the shoulder muscle (rotator cuff) tendons. In order to do this, we will use and assess a predefined set of adverse events (such as frozen shoulder or persistent pain) in practice.
2	**Choice**<br>You are an adult suffering from a tear of the shoulder muscle tendons, which can be treated with an initial, minimally invasive (i.e. arthroscopic) surgical intervention. That is why we are sending you this information leaflet.
3	**General information about the project**<br>The collection of clinical data following surgical interventions is very important, and helps to support an established decision-making process within the orthopaedic field. We are carrying out this project so that we can better evaluate and predict the results following the repair of shoulder muscle tendon tears. This evaluation is being carried out on a representative number of male and female patients in Switzerland.<br>Patient safety is essential here. A list of possible adverse events following the arthroscopic intervention was recently defined by a group of more than 80 experts in the field. We would now like to better understand the development of these adverse events from the patient's point of view.
4	**Procedure**<br>This project will last four years. In total, 970 patients from various clinics in Switzerland and one in Germany will be included in the study within the first year. Various examinations (e.g. measurements of range of motion and strength) will be carried out in the clinic before the operation (= preoperative) and then at the 6- and 12-month postoperative time points. At these times and at 2 years post-surgery, you will receive a questionnaire to complete. We will use ultrasound to check how the tendon is healing twelve months after the operation. Any adverse events will be evaluated independently by the doctor and the patient in question.

Audigé L, *et al. BMJ Open* 2021; 11:e045702. doi: 10.1136/bmjopen-2020-045702

Universitätsspital Basel

5	**Usefulness**<br>You will gain no personal benefit from participating in the project. However, the results could be important to others who have the same condition.
6	**Rights**<br>You decide voluntarily whether you want to participate in this project or not. Your decision does not affect your medical treatment/care and you do not have to justify it.
7	**Duties**<br>If you participate, we ask you to adhere to certain requirements (e.g. attending visits and completing the questionnaires).
8	**Risks**<br>You are not exposed to any additional risk by participating in the project.
9	**Outcomes**<br>You will be informed of new results during the project. We will promptly inform you of any additional findings (known as incidental findings) that are detected during the regular study examinations, which may affect your health directly. Any further course of action will then be discussed in detail with you.
10	**Confidentiality of data and samples**<br>We collect your personal and medical data from you. The Swiss National Science Foundation supports the exchange and reuse of research data. The data will be used for other projects if you give your separate consent. We comply with all legal data protection regulations. All parties involved are bound by confidentiality.
11	**Withdrawal**<br>You can withdraw from the project at any time and no longer participate. The data collected so far are still being evaluated.
12	**Indemnity**<br>You will not receive any compensation for participating in the study. Neither your health insurance provider nor you will incur any additional costs from your participation in the study.
13	**Liability**<br>The liability insurance of the project management is liable for any damages within the scope of the project.
14	**Funding**<br>The project is paid for by the Swiss National Science Foundation.
15	**Contact person:**<br>You can receive information on all your questions at any time:<br><br>PD Dr. Andreas Müller, Senior Consultant, Head Shoulder and Elbow,<br>University Hospital of Basel, Spitalstrasse 21, CH-403 Basel<br>Tel 061 315 25 17 , Email A.Mueller@usb.ch

Audigé L, et al. BMJ Open 2021; 11:e045702. doi: 10.1136/bmjopen-2020-045702

Universitätsspital Basel

More detailed information

1. **Aim of the project**
   The aim of this project is to investigate how the most important results (for example, the occurrence of adverse events or shoulder function) are easily predicted following a surgical intervention to treat tears of the shoulder muscle tendons. Furthermore, we want to investigate how well a predefined set of adverse events, which could occur as part of such a surgical intervention, corresponds to what actually occurs in practice.

2. **Choice**
   Participation is open to anyone with a torn tendon in the shoulder muscles, which can be repaired with minimally invasive (i.e. arthroscopic) surgery.
   Important: This must be the first intervention on the shoulder in question.
   Participation is not open to anyone for whom a detailed medical examination would not be possible or who cannot be called back for check-ups within the follow-up period (e.g. if they live outside Switzerland). Underage persons should also not participate.

3. **General information about the project**
   This project will be carried out in accordance with the laws of Switzerland. The responsible ethics committee has reviewed and approved this project

   The collection of clinical data following surgical interventions is very important. You will help:

   - to assess the effectiveness and safety of the intervention,
   - to make comparisons with other methods,
   - to support an established decision-making process within the orthopaedic field.

   The study intends to document the most important events following arthroscopic interventions, especially in terms of:

   - safety (occurrence of adverse events),
   - healing of the tendon repair,
   - shoulder pain and function,
   - the general state of health and quality of life as well as
   - patient satisfaction.

   Patients' socio-demographic characteristics, examination parameters and treatment parameters will be investigated to enable a prediction of these events to be made.

   In most areas of orthopaedics, there is currently no international standard for the description of adverse events (often known as complications) resulting from surgical interventions. In shoulder orthopaedics, such a list of events does not exist for arthroscopic interventions used in the treatment of tears of the shoulder muscle tendons (rotator cuff). For this reason, the Shoulder and Elbow Surgery research group at the Schulthess Clinic, Zurich, working together with the Orthopaedic and Traumatology Department of the University Hospital of Basel and over 80 experts working in the field of arthroscopy for rotator cuff tears, have defined a list of possible adverse events.

   The aim is to apply and evaluate this predefined list of events. Each event will be evaluated independently by the doctor and the patient in question.

This project is supported by the Swiss National Science Foundation and will last four years. In total, 970 patients will be included in the study within the first year. These patients will be recruited from various clinics in Switzerland and one clinic in Germany. At the University Hospital of Basel about 60 patients are recruited.

4. Procedure

The following diagram shows the course of study events from the time of diagnosis to the follow-up appointment at two years post-surgery.

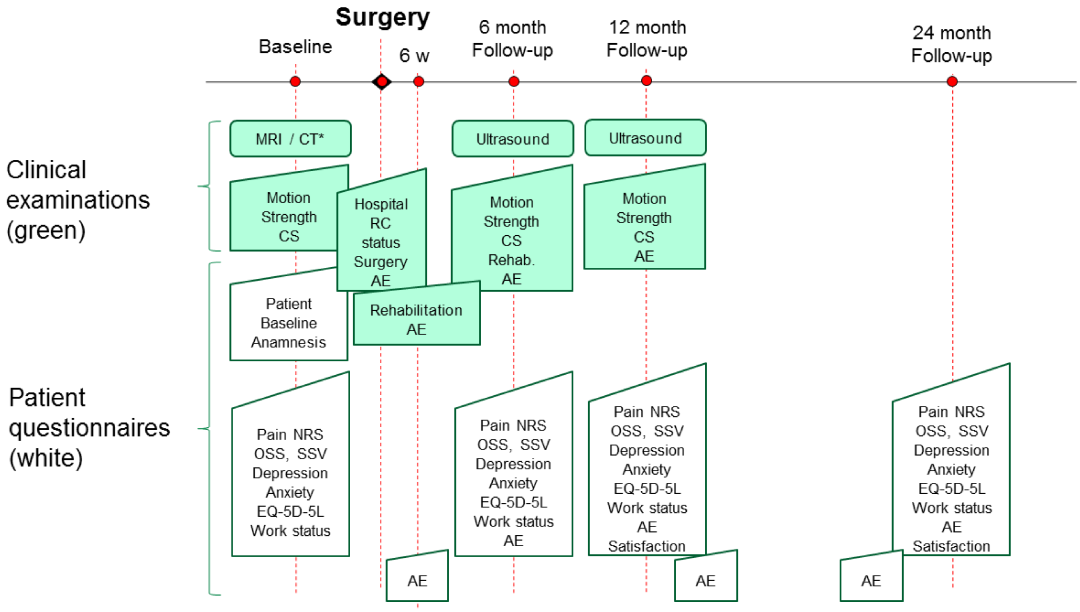

6 W = 6-week follow-up; AE = adverse events

Figure 1: Schematic representation of the study's progress

For your diagnosis, you will be examined by the doctor using various methods (e.g. functional testing, radiological and magnetic resonance imaging (= MRI)) during the first consultation. If you agree to participate in the project, you will receive a questionnaire, which will ask you to provide your personal details and estimate your current level of functional ability in everyday life. Shortly before the operation, your surgeon will record detailed information about your shoulder injury and the surgical intervention. Six weeks after the operation, your surgeon will ask you about your pain levels, rehabilitation programme and the occurrence of any adverse events.
During further follow-up appointments (at 6 and 12 months post-surgery), various examinations (e.g. measurements of range of motion and strength, see figure 1) are routinely performed in the clinic. An independent examiner will carry out an ultrasound routinely 12 months after the operation to document how the tendon is healing. You will also receive a questionnaire to complete. If you wish, you can complete this questionnaire at home - either on paper or electronically by following an invitation link that will be emailed to you. At the end of this document, you will be asked to provide your email address, if you do prefer to receive the electronic version of the questionnaire. Various questionnaires are already routinely recorded at the University Hospital of Basel.

Universitätsspital Basel

Two years after the operation, you will be sent the questionnaire again by post or email (no further check-ups will take place at the clinic). This takes place within the framework of the project. If you have experienced one or more adverse events, the questionnaire will ask you to rate each event by severity and by its relationship to your treatment. The study doctors will evaluate all reported events by severity without knowing the patient, to whom these relate or the clinic in which the events occurred.

Each visit to the clinic (before surgery and 6- and 12-months post-surgery) will last 40 to 60 minutes including all the examinations. Completing the patient questionnaire will take an additional 20 to 30 minutes. An adverse event tends to be evaluated in only a few minutes.

We may have to exclude you from this project prematurely. This can occur if no tear of the rotator cuff can be confirmed intraoperatively (i.e. during the operation) (this occurs very rarely) or if a tendon repair is not possible without additional interventions on the shoulder (a so-called irreparable tear). The study doctor will inform you of any such exclusion from the project.

5. Usefulness

You will not personally benefit from participating in the project. The results can be important for others who have the same condition. As described above, the study aims to enable better evaluation and prediction of the risk of adverse events and the effectiveness of a surgical intervention. We want to support the decision-making processes of future interventions of this kind.

6. Rights

You're volunteering. If you do not want to participate or later withdraw your participation, you do not have to justify this. Your medical treatment/care is guaranteed regardless of your decision. You may ask questions about participation and the project at any time. Please contact the person named at the end of this information.

7. Duties

As a participant, it is necessary that you

- adhere to the necessary specifications and requirements of the project management.
- inform your investigator/project management about the course of the disease and report new symptoms, new complaints and changes in well-being.
- inform your investigator/project management about the simultaneous treatment and therapy with another doctor and about taking medication.

8. **Risks**

You are not exposed to any additional risk by participating in the project.

9. Outcomes

The investigator/project manager will inform you during the project about any new findings that may affect the benefit or your safety and thus your consent to participate. You will be informed of random findings which may contribute to the prevention, detection or treatment of existing or future diseases.

10. Confidentiality of data and samples

Your personal and medical data will be collected for this project. Very few professionals will see your unencrypted data, and only to perform tasks within the scope of the project. Data collection for study purposes is encrypted. Encryption means that all reference data that could identify you (name, date of birth) are deleted and replaced by a key. The key list always remains in the institution/hospital. Those who do not know the key cannot therefore draw any conclusions about you. In the

Audigé L, *et al. BMJ Open* 2021; 11:e045702. doi: 10.1136/bmjopen-2020-045702

case of a publication, the summarised data cannot therefore be traced back to you as an individual. Your name will never appear on the Internet or in any publication. Sometimes there is a requirement in a journal for publication that individual data (so-called raw data) must be transmitted. If individual data must be transmitted, then the data is always encrypted and cannot be traced back to you as a person. All persons who have access to your data within the scope of the project are subject to confidentiality. The requirements of data protection are adhered to and you as a participating person have the right to inspect your data at any time.

If data are stored on site, it is a database for research purposes.

Each centre will encode and save the data centrally in the project database (server location: Schulthess Clinic, Zurich). The data will be deleted 10 years after the end of the project.
It is possible that your data may be used for other investigations (projects) at a later date or that they may be sent to another databank in Switzerland for investigations (further use) not yet defined in more detail. This other database must meet the same standards as the database for this project. For this further use we ask you to sign a further declaration of consent at the very end of this document.

This project may be reviewed by the relevant ethics committee or by the institution that initiated the project. The project manager may need to disclose your personal and medical information for such checks. All persons must maintain absolute confidentiality. We comply with all data protection regulations and will not make your name public either in a publication or on the Internet.

It is possible that your aftercare physician will be contacted to provide information about your medical condition.

11. Withdrawal
You can stop at any time and withdraw from the project if you wish. The data collected so far are still evaluated in encrypted form, otherwise the entire project loses its value. It is not possible to anonymize your data in case of withdrawal, i.e. the data remain encrypted. Please check whether you agree with this before you participate in the project.

12. Indemnity
If you participate in this project, you will not receive any compensation. You or your health insurance company will not incur any costs for participation.

13. Liability
The prerequisites and procedure relating to liability and safeguarding in the event of a claim are legally regulated. If you suffer a health impairment as a result of the study, please contact the study doctor. The institution that is responsible for carrying out the study is liable for the claim, if you can prove that the injury is due to the project-specific examinations. Liability will not be accepted if the project manager can prove that the injury is only minor and temporary, and does not extend beyond the degree expected by current scientific knowledge.

14. Funding
The project is being funded by the Swiss National Science Foundation (SNSF).

15. Contact person(s)
If you have any questions, concerns, or emergencies that arise during or after the project, you can always contact one of these contacts.

Head at the study location:
PD Dr. Andreas Müller, Senior Consultant, Head Shoulder and Elbow
University Hospital of Basel, Spitalstrasse 21, CH-403 Basel
Tel 061 315 25 17 , Email A.Mueller@usb.ch

24-hour emergency number: +41 61 265 25 25

Local project coordination:

PD Dr. Andreas Müller, Senior Consultant, Head Shoulder and Elbow
University Hospital of Basel, Spitalstrasse 21, CH-403 Basel
Tel 061 315 25 17 , Email A.Mueller@usb.ch

Audigé L, *et al. BMJ Open* 2021; 11:e045702. doi: 10.1136/bmjopen-2020-045702

Universitätsspital Basel

Declaration of consent

Written declaration of consent for participation in a study project
Please read this form carefully. Please ask if you do not understand or want to know something

**BASEC number (after submission):**	
**Title of the project (scientific and lay)**:	Surgical safety and effectiveness in orthopaedics: Swiss-wide multicenter evaluation and prediction of core outcomes in arthroscopic rotator cuff reconstruction<br><br>Surgical safeguarding and effectiveness in orthopaedics: Swiss-wide multicentre evaluation and prediction of the most important effects following arthroscopic repair of shoulder tendons (rotator cuff reconstruction)
**Responsible institution (Project management with address)**:	University Hospital of Basel<br>PD Dr. Andreas Müller<br>Orthopaedics and Traumatology<br>Spitalstrasse 21, CH-4031 Basel
**Place of implementation**:	Universitätsspital Basel
**Head of the project at the place of study:**	PD Dr. Andreas Müller
**Participant:**	Name, first name: ____________________<br><br>Date of Birth: ____________________<br><br>☐ Female ☐ Male

The undersigned investigator informed me verbally and in writing about the purpose, the course of the project, about possible advantages and disadvantages as well as about possible risks.

- I voluntarily participate in this project and accept the content of the written information provided on the above mentioned project. I've had plenty of time to make my decision.
- My questions concerning the participation in this project have been answered. I keep the written information and receive a copy of my written consent.
- I agree that the responsible experts of the project management/client of the project and the ethics committee responsible for this project may inspect my unencrypted data for verification and control purposes, but in strict compliance with confidentiality.
- I will be informed of study results or random findings that directly affect my health. If I don't want that, I'll inform my investigator.
- I know that my health-related and personal data can only be passed on in encrypted form for research purposes **for this project**.

Audigé L, et al. BMJ Open 2021; 11:e045702. doi: 10.1136/bmjopen-2020-045702

Universitätsspital Basel

- In the event of further treatment outside the test centre, I authorise my after-treating doctor(s) to forward my after-treatment data relevant to the project to the investigator/project management.
- I can withdraw from participation at any time and without giving reasons, without having any disadvantages in further medical treatment/care. The data collected so far will still be used for the evaluation of the project.
- The liability insurance of the hospital/institution covers any damages.
- I am aware that the obligations stated in the participant information must be complied with.
- If you agree that your email address can be used solely for receiving questionnaires and project-related communications, please enter it here:

______________________________@_____________________

Place, Date	Signature of participant

Confirmation from the investigator: I hereby confirm that I have explained the nature, significance and scope of the project to this participant. I assure you that I will fulfil all obligations in connection with this project in accordance with applicable law. If, at any time during the implementation of the project, I become aware of any aspects that might affect the participant's willingness to participate in the project, I will inform the participant immediately.

Place, Date	Name and first name of the informing investigator in block capitals<br><br>Signature of the investigating physician

Audigé L, *et al. BMJ Open* 2021; 11:e045702. doi: 10.1136/bmjopen-2020-045702

Universitätsspital Basel

Declaration of consent for the further use of data in encrypted form.
(for further use of data of THIS project)

**Participant:**	Name, first name:______________________
	Date of birth: _________________________
	☐ Female ☐ Male

I allow my data from this project to be used in encrypted form for medical research. This means that the data may be stored in a databank and used for future, not yet defined research projects for an indefinite period of time. This consent is unlimited.

I decide voluntarily and can revoke this decision at any time. When I step back, my data is anonymized. I simply inform my investigator/project manager and do not have to justify this decision.

I understand that the data are encrypted and the code is kept safe. The data can be sent to other databanks in Switzerland and abroad for analysis if they comply with the same standards as in Switzerland. All legal requirements regarding data protection are complied with.

Normally, all data are evaluated in their entirety and the results published in summary form. Should a result be relevant for me, it is possible that I will be contacted via my investigator. If I do not wish this, I will inform my investigator/project manager.

If results from the data are handled for commercial purposes, I hereby make no claims on any part of this commercial use.

Place, Date	Signature of participant

Confirmation from the investigator: I hereby confirm that I have explained to this participant the nature, significance and implications of the further use of data.

Place, Date	Name and first name of the informing investigator in block capitals<br><br>Signature of the investigating physician

Audigé L, *et al. BMJ Open* 2021; 11:e045702. doi: 10.1136/bmjopen-2020-045702
