# Supplementary material for: Swiss-wide multicentre evaluation and prediction of core outcomes in arthroscopic rotator cuff repair: protocol for the ARCR_Pred cohort study
Source: BMJ Open. 2021 Apr 22;11(4):e045702. doi: 10.1136/bmjopen-2020-045702 (PMC8070866; doi:10.1136/bmjopen-2020-045702)
Supplement: Author's manuscript [file bmjopen-2020-045702.draft_revisions.pdf]

BMJ Open is committed to open peer review. As part of this commitment we make the peer review history of every article we publish publicly available.

When an article is published we post the peer reviewers' comments and the authors' responses online. We also post the versions of the paper that were used during peer review. These are the versions that the peer review comments apply to.

The versions of the paper that follow are the versions that were submitted during the peer review process. They are not the versions of record or the final published versions. They should not be cited or distributed as the published version of this manuscript.

BMJ Open is an open access journal and the full, final, typeset and author-corrected version of record of the manuscript is available on our site with no access controls, subscription charges or pay-per-view fees (http://bmjopen.bmj.com).

If you have any questions on BMJ Open's open peer review process please email info.bmjopen@bmj.com

BMJ Open

Protocol for the ARCR_Pred cohort-study: Swiss-wide multicenter evaluation and prediction of core outcomes in arthroscopic rotator cuff repair

Journal:	*BMJ Open*
Manuscript ID	bmjopen-2020-045702
Article Type:	Protocol
Date Submitted by the Author:	12-Oct-2020

Complete List of Authors:	Audigé, Laurent ; Schulthess Klinik, Research and Development; University Hospital Basel, Orthopaedic Surgery and Traumatology<br>Bucher, Heiner; University Hospital Basel, Basel Institute for Clinical Epidemiology and Biostatistics<br>Aghlmandi, Soheila; University Hospital Basel, Basel Institute for Clinical Epidemiology and Biostatistics<br>Stojanov, Thomas; University Hospital Basel, Orthopaedic Surgery and Traumatology; University Hospital Basel, Basel Institute for Clinical Epidemiology and Biostatistics<br>Schwappach, David; Patient Safety Foundation; University of Bern, Institute of Social and Preventive Medicine<br>Hunziker, Sabina; University Hospital Basel, Medical Communication / Psychosomatic Medicine<br>Candrian, Christian; Ospedale Regionale di Lugano, Trauma and Ortho Unit<br>Cunningham, Gregory; Hirslanden Clinique La Colline, Shoulder Center; Geneva University Hospitals, Division of Orthopaedics and Trauma Surgery, Department of Surgery<br>Durchholz, Holger; Klinik Gut Sankt Moritz<br>Eid, Karim; Baden Cantonal Hospital, Clinic for Orthopaedics and Traumatology<br>Flury, Matthias; In-Motion, Center for Orthopaedics and Neurosurgery<br>Jost, B; Cantonal Hospital of St.Gallen, Clinic for Orthopaedic Surgery and Traumatology of the Musculoskeletal system<br>Ladermann, Alexandre; La Tour Hospital, Division of Orthopaedics and Trauma Surgery; Geneva University Hospitals, Division of Orthopaedics and Trauma Surgery, Department of Surgery<br>Moor, Beat; Hôpital de Martigny Centre Hospitalier du Valais Romand, Service for Orthopaedics and Traumatology of the Musculoskeletal system<br>Moroder, Philipp; Charite Universitatsmedizin Berlin, Department of Shoulder and Elbow Surgery, Center for Musculoskeletal Surgery,<br>Rosso, Claudio; Arthro Medics, Shoulder and Elbow Center<br>Schär, Michael; Inselspital Universitatsspital Bern, Department of Orthopaedic Surgery and Traumatology<br>Scheibel, Markus; Schulthess Klinik, Shoulder and Elbow Surgery,; Charité Universitätsmedizin Berlin, Department for Shoulder and Elbow surgery - Center for Musculoskeletal Surgery- Campus Virchow<br>Spormann, Christophe; Endoclinic, Center for Endoprosthetics and Joint

	Surgery<br>Suter, Thomas; Canton Hospital Baselland, Orthopaedic Shoulder and Elbow<br>Wieser , Karl ; University of Zurich, Department of Orthopaedics, Balgrist University Hospital<br>Zumstein, Matthias; Orthopaedics Sonnenhof, Shoulder, Elbow and Orthopaedic Sports Medicine<br>ARCR_Pred Study Group, Name; University Hospital Basel<br>Müller, Andreas; University Hospital Basel, Orthopaedic Surgery and Traumatology
Keywords:	ORTHOPAEDIC & TRAUMA SURGERY, Musculoskeletal disorders < ORTHOPAEDIC & TRAUMA SURGERY, Shoulder < ORTHOPAEDIC & TRAUMA SURGERY

SCHOLARONE™
Manuscripts

I, the Submitting Author has the right to grant and does grant on behalf of all authors of the Work (as defined in the below author licence), an exclusive licence and/or a non-exclusive licence for contributions from authors who are: i) UK Crown employees; ii) where BMJ has agreed a CC-BY licence shall apply, and/or iii) in accordance with the terms applicable for US Federal Government officers or employees acting as part of their official duties; on a worldwide, perpetual, irrevocable, royalty-free basis to BMJ Publishing Group Ltd ("BMJ") its licensees and where the relevant Journal is co-owned by BMJ to the co-owners of the Journal, to publish the Work in this journal and any other BMJ products and to exploit all rights, as set out in our licence.

The Submitting Author accepts and understands that any supply made under these terms is made by BMJ to the Submitting Author unless you are acting as an employee on behalf of your employer or a postgraduate student of an affiliated institution which is paying any applicable article publishing charge ("APC") for Open Access articles. Where the Submitting Author wishes to make the Work available on an Open Access basis (and intends to pay the relevant APC), the terms of reuse of such Open Access shall be governed by a Creative Commons licence – details of these licences and which Creative Commons licence will apply to this Work are set out in our licence referred to above.

Other than as permitted in any relevant BMJ Author's Self Archiving Policies, I confirm this Work has not been accepted for publication elsewhere, is not being considered for publication elsewhere and does not duplicate material already published. I confirm all authors consent to publication of this Work and authorise the granting of this licence.

For peer review only

Protocol for the ARCR_Pred cohort-study: Swiss-wide multicenter evaluation and prediction of core outcomes in arthroscopic rotator cuff repair

Laurent Audigé[1,2,3†], Heiner C. Bucher[3], Soheila Aghlmandi[3], Thomas Stojanov[1,2,3], David Schwappach[4,5], Sabina Hunziker[6], Christian Candrian[7], Gregory Cunningham[8,9], Holger Durchholz[10], Karim Eid[11], Matthias Flury[12], Bernhard Jost[13], Alexandre Lädermann[9,14], Beat Moor[15], Philipp Moroder[16], Claudio Rosso[17], Michael Schär[18], Markus Scheibel[16,19], Christoph Spormann[20], Thomas Suter[21], Karl Wieser[22], Matthias A. Zumstein[23,24], ARCR_Pred Study Group*, Andreas Müller[1]

Author affiliations:

1. Orthopaedic Surgery and Traumatology, University Hospital of Basel, Basel, Switzerland
2. Research and Development Department, Schulthess Klinik, Zurich, Switzerland
3. Basel Institute for Clinical Epidemiology and Biostatistics, University Hospital of Basel, Basel, Switzerland
4. Swiss Patient Safety Foundation, Zurich, Switzerland
5. Institute of Social and Preventive Medicine (ISPM), University of Bern, Bern, Switzerland
6. Medical Communication / Psychosomatic, University Hospital of Basel, Basel, Switzerland
7. Trauma and Ortho Unit, Ospedale Regionale di Lugano, Lugano, Switzerland
8. Shoulder Center, Hirslanden Clinique la Colline, Geneva, Switzerland
9. Division of Orthopaedics and Trauma Surgery, Department of Surgery, Geneva University Hospitals, Geneva, Switzerland
10. Klinik Gut, St Moritz, Switzerland
11. Clinic for Orthopaedics and Traumatology, Cantonal Hospital of Baden, Baden, Switzerland
12. Center for Orthopaedics and Neurosurgery, In-Motion, Wallisellen, Switzerland
13. Clinic for Orthopaedic Surgery and Traumatology of the Musculoskeletal system , Cantonal Hospital of St.Gallen, St.Gallen, Switzerland
14. Division of Orthopaedics and Trauma Surgery, La Tour Hospital, Meyrin, Switzerland
15. Service for Orthopaedics and Traumatology of the Musculoskeletal system , Hôpital du Valais – Centre Hospitalier du Valais Romand, Martigny, Switzerland

For peer review only

For peer review only - http://bmjopen.bmj.com/site/about/guidelines.xhtml

16. Department for Shoulder and Elbow surgery - Center for Musculoskeletal Surgery- Campus Virchow, Charitè Medicine University, Berlin, DE

17. Shoulder and Elbow Center, ARTHRO Medics, Basel, Switzerland

18. Department of Orthopaedic Surgery and Traumatology, Inselspital, Bern University Hospital, University of Bern, Switzerland

19. Shoulder and Elbow Surgery, Schulthess Klinik, Zurich, Switzerland

20. Center for Endoprosthetics and Joint Surgery, Endoclinic, Zürich, Switzerland

21. Orthopaedic Shoulder and Elbow, Canton Hospital Baselland, Bruderholz, Switzerland

22. Department of Orthopaedics, Balgrist University Hospital, University of Zurich, Switzerland

23. Shoulder, Elbow and Orthopaedic Sports Medicine, Orthopaedics Sonnenhof, Bern, Switzerland

24. Stiftung Lindenhof, Campus SLB, Swiss Institute for Translational and Entrepreneurial Medicine, Bern, Switzerland

[†]Corresponding author:

Laurent Audigé, DVM PhD

Schulthess Klinik

Research and Development - Shoulder and Elbow Surgery

Lengghalde 2, CH-8008 Zurich, Switzerland

Phone: +41 44 385 7580; Fax: +41 44 385 7590

E-mail: laurent.audige@kws.ch

For peer review only

* Other members of the ARCR_Pred Study Group are listed below per site:

ARTHRO Medics, Basel, Switzerland (ART): Jannine Buchschacher, Lena Fankhauser, Corinne Eicher, Gernot Willscheid; **Charitè Medicine University, Berlin, Germany (BER)**: Doruk Akgün, Kathi Thiele, Marvin Minkus, Victor Danzinger, Katrin Karpinski; **Canton Hospital Baselland, Bruderholz, Switzerland (BRU)**: Sebastian Mueller, Claudia Haag-Schumacher; **Centre Hospitalier du Valais Romand, Martigny, Switzerland (CHV)**: Viviane Steffen, Sarah Fournier, Deborah Marietan, Sebastien Pawlak; **Endoclinic, Zürich,**

For peer review only - http://bmjopen.bmj.com/site/about/guidelines.xhtml

Switzerland (END): Britta Hansen, Ferdinand Lovrek, Marco Zanetti, Nadja Mamisch; **Klinik Gut, St Moritz, Switzerland (GUT)**: Christian Steiner, Georg Ahlbäumer, Jakob Bräm, Jens Fischer, Alexander Delvendahl; **Hirslanden Clinique la Colline, Geneva, Switzerland (HIR)**: Patricia Simao, Abed Khourani; **La Tour Hospital, Meyrin, Switzerland (HUG)**: Anne-Sophie Foucault, Frank Kolo; **Inselspital, Bern University Hospital, Bern, Switzerland (INB)**: Adrian Schenk, Johannes Weihs, Remy Flückiger, Philipp Scacchi, Paolo Lombardo; **In-Motion, Wallisellen, Switzerland (INM)**: Larissa Hübscher, Ralph Berther, Christine Ehrmann; **Cantonal Hospital of Baden, Baden, Switzerland (KSB)**: Raffaela Nobs, Richard Niehaus, Nisha Grünberger, Philipp Kriechling, Susanne Bensler; **Schulthess Klinik, Zurich, Switzerland (KWS)**: Hans-Kaspar Schwyzer, Fabrizio Moro, Michael Glanzmann, Barbara Wirth, Christian Jung, Florian Freislederer, Manuela Nötzli, Frederik Bellmann, Franz Anne, Jörg Oswald, Alex Marzel, Cécile Grobet, Marije de Jong, Martina Wehrli, Jan Schätz; **Ospedale Regionale di Lugano, Lugano, Switzerland (LUG)**: Francesco Marbach, Marco Delcogliano, Davide Previtali, Florian Schönweger, Elena Porro, Gabriela Induni-Lang, Giuseppe Filardo, Filippo Del Grande, Pietro Feltri, Schiavon Guglielmo; **Cantonal Hospital of St.Gallen, St.Gallen, Switzerland (SGA)**: Christian Spross, Martin Olach, Michael Badulescu, Vilijam Zdravkovic, Stephanie Lüscher, Jörg Scheler, Lena Öhrström; **Orthopädie Sonnenhof, Berne, Switzerland (SON)**: Annabel Hayoz, Frederick Schuster, Julia Müller-Lebschi; **Balgrist University Hospital, Zurich, Switzerland (UKB)**: Christian Gerber, Samy Bouaicha, Paul Borbas, Anita Hasler, Florian Grubhofer, Sabrina Catanzaro, Sabine Wyss, Reto Sutter; **University Hospital of Basel, Basel, Switzerland (USB)**: Mohy Taha, Cornelia Baum, Ilona Ahlborn, Simone Hatz, Giorgio Tamborrini-Schütz; **University Library Basel, Basel, Switzerland**: Christian Appenzeller-Herzog.

For peer review only - http://bmjopen.bmj.com/site/about/guidelines.xhtml

Administrative information

Title Protocol for the ARCR_Pred cohort-study: Swiss-wide multicenter evaluation and prediction of core outcomes in arthroscopic rotator cuff repair

Trial registration ClinicalTrial.gov registration number NCT04321005

Protocol version Version 2 (13.12.2019)

Funding This project is funded by the Swiss National Science Foundation (SNF Project ID 320030_184959, http://p3.snf.ch/project-184959). A complementary grant was provided by Swiss Orthopedics. The sites Charitè Medicine University, Berlin, Germany (BER) and University Clinic Balgrist, Zurich, Switzerland (UKB) are funding their own participation in the project.

Roles and responsibilities

LA and AM are the initiators and project leaders. LA, AM, HB, SA, DS and SH were involved in the study design, which was reviewed and commented by principal investigators CC, GC, HD, KE, MF, BJ, AL, BM, PM, CR, MS, MS, CS, TS, KW and MZ.

Preparation of the manuscript was done by LA, AM, SA and TS. HB, DS and SH edited and critically revised the paper. All authors have read and approved the manuscript. LA is the guarantor of the manuscript.

This is an investigator initiated project at the University Hospital of Basel. The principal investigator and project leader (AM) is the official sponsor representative for the project and was involved in all phases of the project from its conception to the current implementation steps. The project initiators and project leaders (LA and AM) have ultimate authority over any of the project activities.

A project scientific board (PSB) comprises the project leaders (LA and AM), project investigators at each site (CC, GC, HD, KE, MF, BJ, AL, BM, PM, CR, MS, MS, CS, TS, KW

For peer review only

and MZ), and project partners (SA, HB, DS and SH). The PSB shall meet at specific time points during the study: before the study start, after completion of recruitment and the 12-month follow-up, and at the end of the study. The agenda of these meetings will focus on (however is not limited to) patient enrollment and the completion of follow-up examinations and questionnaires, the documentation process in REDCap, data quality issues (completeness and consistency), monitoring activities, adverse event assessment and management, baseline patient description, ranking of prognostic factors for prognostic models, progress of data analysis, publication strategy and decisions regarding data sharing. Between these meetings, communication will be maintained between the project coordinating team and investigators via various channels including emails, quarterly newsletters, phone calls and (video) conference calls as required.

For peer review only

For peer review only - http://bmjopen.bmj.com/site/about/guidelines.xhtml

Abstract

Introduction In the field of arthroscopic rotator cuff repair (ARCR), reporting standards of published studies differ dramatically, notably concerning adverse events (AEs). In addition, prognostic studies are overall methodologically poor, based on small datasets, and explore only limited numbers of influencing factors. We aim to develop prognostic models for individual ARCR patients, primarily for the patient-reported assessment of shoulder function (Oxford Shoulder Score [OSS]) and the occurrence of shoulder stiffness 6 months after surgery. We also aim to evaluate the use of a consensus Core Event Set (CES) for AEs and validate a severity classification for these events, considering the patient's perspective.

Methods and analysis A cohort of 970 primary ARCR patients will be prospectively documented from several Swiss and German orthopedic clinics up to 24 months postoperatively. Patient clinical examinations at 6 and 12 months will include shoulder range of motion and strength (Constant Score). Tendon repair integrity status will be assessed by ultrasound at 12 months. Patient-reported questionnaires at 6, 12 and 24 months will determine functional scores (Subjective Shoulder Value, OSS), anxiety and depression scores, working status, sports activities, quality of life (EuroQol EQ-5D-5L). AEs will be documented according to a CES. Prognostic models will be developed using an internationally supported regression methodology. Multiple prognostic factors, including patient baseline demographics, psychological, socioeconomic and clinical factors, rotator cuff integrity, concomitant local findings, and (post)operative management factors will be investigated.

Ethics and dissemination This project contributes to the development of personalized risk predictions for supporting the surgical decision process in ARCR. The consensus CES may become an international reference for the reporting of complications in clinical studies and registries. Ethical approval was obtained on April 1st, 2020, from the lead ethics committee

For peer review only

(EKNZ, Basel Switzerland; ID: 2019-02076). All participants will provide informed, written consent before enrollment in the study.

Strengths and limitations of this study

- Large prospective multicenter observation of routine care
- Assessment of patient-reported outcomes (PROMs)
- Implementation of an international core outcome set of adverse events (CES)
- Internationally supported methodology for prognostic model development
- Potential limited response to patient questionnaires at 24 months

Introduction

Rotator cuff tears are one of the most common injuries of the shoulder joint, which may cause pain and disability associated with severe restrictions in daily activities. Surgical repair is indicated when nonoperative treatment fails or follows extended traumatic tears, notably inactive patients without signs of advanced tendon degeneration or muscle fat infiltration [1]. Clinical studies have demonstrated clinically-relevant improvement in shoulder function and quality of life after arthroscopic rotator cuff tear repair (ARCR) [2-5]. The number of ARCRs has increased over the last two decades [6 7 8 9] due to several contributing factors such as an aging yet active population, improvements in operative repair techniques, and more liberal indications for ARCRs.

Not all patients, however, benefit from ARCR [10]. Patients may be affected by complications and/or adverse events (AEs) like persistent pain, shoulder stiffness, infection, neurological problems, and repair failures [11 12]. About 20% of patients may show, typically between 6 and 12 months following ARCR, a persistent rotator cuff defect [13]. Patients with healed tendons may show better functional outcome after repair [2 14 15]. Postoperative shoulder stiffness, a major complication reported to occur in 1.5% to 11.1% of ARCRs [11], leads to limitations in everyday activities, prolonged rehabilitation, and, in severe cases, to reoperation (capsular

For peer review only - http://bmjopen.bmj.com/site/about/guidelines.xhtml

release) [16-18]. Nonetheless, incident data on outcome and AEs are impaired by the heterogeneity in definition and reporting [13 19].

Valid and representative data on the safety and effectiveness of ARCR are nonexistent at the Swiss national level. However, such data is paramount for optimizing the indication and outcome of ARCR, and for benchmarking orthopedic clinics. Reporting standards are a prerequisite for outcome and safety data. Recently, a Core Outcome Set (COS) [20] was defined for shoulder disorders, which includes inner core domains of pain, physical function and activities, global perceived effect (a person's assessment of their recovery or degree of improvement), and AEs [21 22]. A Core Event Set (CES) was developed by international consensus in ARCR [23 24] and lay the ground for the current project.

Appropriate indication of ARCR and judgment on risks for AEs or unsatisfactory patient outcomes rely on validated clinical prediction tools [25 26], which are still sparse in the field of surgical repair of a rotator cuff tear. Currently existing models focus on early surgical repair [27], tendon healing [28 29] or shoulder functional outcomes [30]. A model for shoulder stiffness included patients with various shoulder pathologies and surgeries [31]. Furthermore, individual outcome predictions in ARCR require the identification of relevant patient and management factors. Several systematic reviews have highlighted the general lack of qualitative studies focused on prognostic factors for ARCR outcomes [32-36]. In addition, we have observed the substantial heterogeneity in terms of applied methodology, core outcomes and studied prognostic factors, where certain factors (e.g., age, tear size, muscle degeneration, smoking) are given greater focus over others (e.g., sex, traumatic onset). The reviews highlight the need for more robust prospective studies to include additional patient-reported outcomes in a multivariable context.

For peer review only - http://bmjopen.bmj.com/site/about/guidelines.xhtml

Objectives

The overall objective is to establish a prospective cohort of patients undergoing ARCR with standardized data collection and follow-up for the evaluation and prediction of targeted core safety, and clinical and patient-reported outcome parameters that are to be routinely collected in standard clinical care.

The primary objective is to develop predictive models for two core outcome parameters:, 1) the patient-reported Oxford Shoulder Score (OSS) functional outcome, and 2) the occurrence of shoulder stiffness (primary safety event) as reported by patients and clinicians.

Secondary objectives are 1) to evaluate the content and applicability of the defined consensus CES (i.e. ARCR CES 1.0) [23] in routine practice considering the patient's perspective, 2) to quantify the incidence of AE up to 24 months after surgery (e.g., persisting or worsening pain, recurrent rotator cuff (RC) defect), 3) to validate an adapted severity classification for postoperative local AEs [12 37], and 4) to develop predictive models for other clinically-relevant outcome parameters including patient-reported outcomes (e.g., perception of improvement, return to work, return to sports, quality of life, satisfaction with surgery, acceptability of symptom state), clinical outcomes (e.g., shoulder strength and motion) and specific AEs (e.g. RC defect at 12 months).

Methods and analysis

Study design and setting

This is a prospective multicenter cohort study in patients undergoing ARCR with 17 participating orthopedic centers in Switzerland and one German center.

Several sub-projects, associated with the main ARCR cohort study, are planned and include a systematic review of prognostic studies in ARCR, the application of the ARCR CES 1.0 for AE documentation, and the application and validation of an AE severity classification

For peer review only - http://bmjopen.bmj.com/site/about/guidelines.xhtml

Eligibility criteria

Adult patients diagnosed with a partial or full-thickness RC tear by magnetic resonance imaging (MRI), planned for a primary arthroscopic surgical repair, and giving their informed consent to participate in the cohort study will be included. Patients undergoing a specific surgical procedure for irreparable tears (i.e. tendon transfer, subacromial spacer or superior capsular reconstruction), revision operations, open or mini-open reconstructions will be excluded. Patients unable to give written informed consent or attend clinics for follow-up visits, not fluent in German, French, Italian, or English or pregnant females will be excluded. Patients undergoing bilateral ARCR will only be included for their first intervention.

Intervention

Shoulder arthroscopies will be performed according to standardized clinic-specific and international guidelines [38] in the context of routine care with patients in a beach-chair or lateral decubitus position under general or local anesthesia. The variability in the repair techniques used between clinics and surgeons will be documented. Typically, after the diagnostic arthroscopy to assess the type of RC tear (partial or full-thickness tear and involved tendons, tendon tear delamination, sign of tendon degeneration) and concomitant injuries or lesions, the ruptured tendons are mobilized until they can be repositioned on the original footprint with as little tension as possible. Tendon fixation may be performed using one of multiple anchor and suture configurations according to the surgeon's decision. An intervention at the biceps tendon is performed if any tendinopathy, or lesions to the superior labrum or biceps pulley system are observed. An anterolateral or lateral acromioplasty is performed at the surgeon’s discretion, generally in the presence of a hooked-shaped acromion or a critical shoulder angle larger than 35°, respectively. Operative details, including additional concomitant procedures (acromioplasty, acromioclavicular joint resection, capsulotomy, and biceps tenotomy or tenodesis) and operation duration are recorded immediately after surgery. A standard 3-phase postoperative rehabilitation scheme

For peer review only - http://bmjopen.bmj.com/site/about/guidelines.xhtml

is usually prescribed and will be documented in detail, including immobilization and passive mobilization in the first phase, active mobilization and coordination training in the second phase, followed by the third phase of specific progressive resistance exercises.

Outcomes

The *first primary outcome* is the patient-reported change in shoulder functional outcome between baseline and 6 months postoperatively as measured with the Oxford Shoulder Score (OSS). [39] The OSS is a condition-specific questionnaire developed for patients with a degenerative or inflammatory state of the shoulder. It contains twelve items to be answered by the patient independently, which deal with pain (degree, time point) and possible handicaps in private and professional life. There are five categories of response for every question, corresponding to a score ranging from 0 to 4. Scores are summed to give a single score with a range from 0 (worst outcome) to 48 (best outcome). Transcultural validations of this questionnaire for the German and Italian populations have been performed [40 41] and are validated for patient-based outcomes after RC repair [42-44]. While functional outcome at the last 24-month follow-up is clinically relevant, the early 6-month primary time point is chosen because of the importance in early surgical recovery and rehabilitation, particularly when considering the socioeconomic impact on professionally active patients [4].

The *second primary outcome* is the occurrence of shoulder stiffness within 6 months after surgery: this event is poorly defined in the literature [19]. We formed a consensus definition of shoulder stiffness among specialized shoulder surgeons in a Delphi survey, which describes a postoperative restriction in passive shoulder motion diagnosed within 6 months after ARCR in at least two of the motion planes of flexion, abduction and external rotation in 0° abduction. Motion restriction is to be assessed separately for each plane according to specific threshold criteria (flexion: total motion equal to or below 90° or glenohumeral motion equal to or below 80°; abduction: total motion equal to or below 80° or glenohumeral motion equal to or below 60°; external rotation in 0° abduction: glenohumeral motion equal to or below 20° or no more

For peer review only - http://bmjopen.bmj.com/site/about/guidelines.xhtml

than 50% of the contralateral side value). In this project, we will identify cases of shoulder stiffness based on our consensus definition as well as clinical records and reports from clinicians and their patients.

Secondary outcomes will include: 1) local AEs according to the ARCR CES, in particular the occurrence of recurrent defect of repaired tendon(s) at 12 months, when at least one repaired tendon is diagnosed with a recurrent defect by ultrasound examination, persistent or worsening pain, infection, any local event (composite outcome); 2) functional parameters of the Constant score (CS) [45] at 6 and 12 months, shoulder strength (kg) in abduction at 6 and 12 months, patient-reported shoulder pain on the numeric rating scale (NRS) at 6, 12 and 24 months, patient-reported shoulder function: OSS at 6, 12 and 24 months, Subjective Shoulder Value (SSV) [46] assessment at 6, 12 and 24 months; 3) general health and socioeconomic parameters including patient-reported quality of sleep (NRS) at 6, 12 and 24 months [47], return to work, change of working condition within 6, 12 and 24 months, level of depression and anxiety at 6, 12 and 24 months based on Patient-Reported Outcomes Measurement Information System (PROMIS) scores [48 49], patient perceived shoulder improvement, acceptability of own symptom state[50], quality of life (utilities and general health) at 6, 12 and 24 months using the European Quality of Life 5 Dimensions 5 Level questionnaire (EQ-5D-5L), patient satisfaction with the surgical outcome at 12 and 24 months; 4) safety outcome assessment, occurrence of all AEs reported by clinicians and patients (including non-local AEs within 6 months after surgery), final independent surgeon and patient-rated assessment of AEs according to perceived severity (rating scale from 0 [no complication] to 100 [death] [51]), comprehensive Complication Index[51] considering all AEs that occurred within 6 months after surgery.

Shoulder ultrasound examinations will be performed at 12 months by experienced clinicians independent of the operating surgeons. The repair integrity will be graded according to the Sugaya classification (where grade 4 or 5 defines the occurrence of a recurrent effect) [52 53].

For peer review only - http://bmjopen.bmj.com/site/about/guidelines.xhtml

Other ultrasound parameters include the location of the recurrent defect (at the footprint | medial cuff failure), long biceps tendon status, signs of anchor displacement, and location and signs of suture cut-through.

Participant timeline

Local investigators will identify patients who meet the eligibility criteria. Patient enrollment started on June 1st, 2020 and is planned for a maximum period of 12 months. Patients will complete a preoperative evaluation no more than 2 months before surgery. Follow-up assessments will be performed at 6 weeks (+/- 1 week), and 6 (+/- 1 month), 12 (+/- 1 month) and 24 months (+/- 2 months) postoperatively. At the final 24-month time point, only patient self-reporting assessments will be documented (Figure 1).

Baseline prognostic factors

Various baseline parameters, operative details, and postoperative management variables are known or suspected to influence ARCR outcomes [32-36].

The following patient-related factors will be recorded: patient demographics [year of birth for age, sex], socioeconomic parameters [nationality, marital status, the highest level of education, employment status, last occupational position, daily physical workload], dominant side, smoking & drinking status, general physical and mental health [Body Mass Index (BMI) and obesity, American Society of Anesthesiologists (ASA) classification, comorbidities (e.g. diabetes), concomitant medication, level of depression and anxiety (PROMIS Depression and Anxiety Short Form 4a) [48 49], quality of life (EQ-5D-5L) [54]].

Disease-related factors are shoulder clinical examinations [pain level on a numeric rating scale (NRS), range of motion, muscle strength, Constant Score [45]], patient-reported shoulder function (see outcome measures), radiograph parameters [Critical Shoulder Angle [55], acromiohumeral distance [56]], MRI or arthro-Computer Tomography (CT) parameters

For peer review only - http://bmjopen.bmj.com/site/about/guidelines.xhtml

[supraspinatus muscle atrophy [57], tangent sign [58], grade of fatty infiltration [59 60]], medical history [cause of injury (trauma event), symptom duration, previous interventions (operation and timing of surgery), actual medication, and the extent of physical therapy].

Rotator cuff integrity and concomitant local findings. The RC tear will be determined by MRI (or arthro-CT) and confirmed intraoperatively: tear size: location (involved tendons) and grading (partial / complete), tendon retraction grade [61] and tear sagittal size [62] , status of the biceps tendon, additional intraoperative observation of concomitant local injuries [Superior Labrum from Anterior to Posterior (SLAP) lesion, Humeral Avulsion Glenohumeral Ligament (HAGL), Bankart lesion, humeral and glenoid-side chondral lesions].

Operative details and postoperative management: type of ARCR procedure [use of anchors, suture techniques], augmentation techniques [e.g. platelet concentrates, scaffolds, …] [63], additional concomitant treatment [acromioplasty, acromioclavicular joint resection, capsulotomy, biceps tenotomy or tenodesis, treatment of SLAP lesion], operation duration, duration of hospital stay, postoperative management [immobilization position and duration, pain medication [e.g. using non-steroidal anti-inflammatory drugs], timing of passive and active shoulder motion, physiotherapy and muscle training].

Adverse event documentation and assessment process

Operating surgeons will report the occurrence of any intraoperative AE on the operation form. The occurrence of postoperative local AEs within 24 months will be reported by investigators at the clinical examination and by patients on the questionnaires. The occurrence of postoperative non-local AEs that are unrelated to the operation will be documented in a similar manner, however only within 6 months after surgery. An AE form was developed according to the ARCR CES 1.0 [23]. Each AE documentation will be structured after Audigé et al. [64] and includes the date/period of occurrence [intra- / postoperative], the affected body location [local at the operated shoulder / non-local], the event group and

For peer review only - http://bmjopen.bmj.com/site/about/guidelines.xhtml

specification, applied health-related intervention(s) [operative / nonoperative procedure(s)], its outcome at the time of reporting (or end of the study), and the assessment of the event [causal factor(s) / severity grade / seriousness]. Severity grading will be made according to existing and adapted systems for intraoperative [65 66] and postoperative [12 37] complications. The documentation of AEs will be checked for completeness and consistency remotely as well as on-site by reviewing selected patient files as part of the monitoring plan. A review committee (LA, AM, TS, HD and DS) will assess all events reported by surgeons and patients, and generate queries to the respective sites as required.

Fully documented local events, including their treatment, outcome and possible causative factors, will be formulated in layman's terms and sent back to the affected patients, so that they can confirm and validate collected AE data as well as assess their severity on a visual analogue scale from 0 (not at all severe) to 100 (extremely severe). This subsequent rating will also be performed by their treating surgeon and four other randomly-selected surgeons involved in the project, blinded from the original severity grading.

Sample size determination

For sample size calculation, we set up a simulation study and used multiple regression to predict the change in OSS within 6 months for the most important prognostic factors. The prognostic factors were derived from an existing ARCR local registry [67] and include age, sex, body mass index/obesity status, tendon quality/degeneration, and RC severity [32-36]. We accounted for the type I error at 5% for statistical significance and the type II error set at 20% for 80% statistical power [68]. Two thousand replications were done, and the p-values were recorded to calculate the mean significance for each of the prognostic factors to reach a minimum of 80% statistical power. This approach led to a sample of 920 patients.

For the second primary outcome of shoulder stiffness, we accounted for a minimum of ten events per variable to allow for the inclusion of a maximum of ten predictors into the model [69]

For peer review only - http://bmjopen.bmj.com/site/about/guidelines.xhtml

[70]. The estimated event rate for shoulder stiffness from our pilot data set was 8.3%, which, according to our experience, might reflect an underestimation of the true rate [17]. Therefore, a 10% stiffness rate was assumed, which resulted in a sample size calculation of 900 patients. The higher resulting number determines the final number of patients to be recruited. Therefore, 920 patients will be included with an additional 50 patients (i.e. 970 patients) due to the anticipated maximum dropout rate of 5% at 6 months (based on personal experience).

Recruitment

Study sites and local investigators were selected based on their expertise in ARCR with support by the shoulder and elbow expert group of Swiss Orthopaedics. Each site was visited by the project leaders to assess the adequacy of local clinical and research settings for the project as well as to ensure prior interest and commitment. The number of included sites was determined based on the reported estimate of the number of ARCR patients that could be realistically enrolled within one year from each site, and included an allowance for overestimation (i.e. all sites together estimated that they could recruit up to 40% more than the expected 970 patients within one year).

Patients who are enrolled after signing an informed consent form are definitively recruited for the project after documentation of baseline parameters (clinical examinations and patient questionnaires) and confirmation of ARCR during surgery. A recruitment curve is prepared every 2 weeks and sent to the project sites along with a recruitment table presenting the performance of each site. Sites that are unable to recruit the expected number of patients within the first 3 months will be considered for exclusion from the project and replaced by additional sites if the estimated total duration of patient enrollment is delayed for more than 3 months.

Data collection methods

Data are collected on electronic or paper-based case report forms or patient questionnaires. Project parameters and used instruments are presented in previous sections of this protocol.

For peer review only - http://bmjopen.bmj.com/site/about/guidelines.xhtml

For peer review only

A training video was prepared for the collection of CS data [45]. For the measurement of shoulder muscle strength, several devices were permitted, i.e. IsoForceControl® (MDS Medical Device Solution, Oberburg, Switzerland), Mark-10 Force Gauge (Mark-10 Corporation, Copiague, USA) as well as hand-held (Lafayette Instrument Co., Lafayette, USA) or MicroFET 2 (Hoggan Scientific, Salt Lake City, USA) dynamometers; the use of a spring balance was not allowed.

Patient clinical examinations, including baseline imaging assessments, are performed at each site by experienced clinicians (who may be assisted by locally-trained research staff) and documented primarily on paper-based case report forms. Baseline MRI and radiographs are coded and centralized at the University of Basel to ensure data quality control. Operative data are collected electronically by the respective surgeons shortly after surgery. Patients complete questionnaires in their preferred language, which is limited to German, French, Italian or English, either electronically after invitation, by email or on a tablet computer at the site, or otherwise on paper. AEs are documented electronically by the respective surgeons with support from their research staff. Data collected on paper forms are entered electronically at each site or at a central location at the University of Basel based on the agreement made with each site.

Data monitoring

A central project data manager will perform data quality control on all collected data. A flowchart will be created to describe the number of consecutively recruited patients who had an RCR by arthroscopic procedure or had a conversion to an open procedure, and who completed follow-up clinical and imaging examinations as well as self-reported outcome questionnaires. The reasons for patient dropout and loss to follow-up status will be monitored and described. All recorded study parameters will be described using standard descriptive statistics; continuous variables will be presented as means with standard deviations and

For peer review only

For peer review only - http://bmjopen.bmj.com/site/about/guidelines.xhtml

categorical variables as counts with percentages. The variability of data between clinics will be explored to support the identification of outlier data.

Weekly site-specific reports, including the patient enrollment list, expected follow-up timing and identification of missing, erroneous or inconsistent data, are sent to the respective local project staff. Data-related queries will be resolved remotely or by on-site monitoring visits before the final analyses are performed.

There is no plan for auditing project conduct other than via reporting at the annual meetings of the project scientific board.

Data management

Study data will be stored using the REDCap web-based electronic data capture system [71 72] on a server that is hosted at Schulthess Klinik. REDCap conforms with Good Clinical Practice guidelines that provide required features for data protection and integrity, e.g., password-protected access and change tracking.

Study data will be coded and exported from the REDCap system into Stata software (StataCorp LP, College Station, USA) for statistical analyses. Data transformations and analyses will be primarily implemented using Stata and fully documented within Stata programming files. Data subsets will be prepared for analyses using alternative software (e.g. R for prediction models) as appropriate.

All patients with an intraoperatively confirmed RC tear and operated by ARCR will be included in the analyses. Existing missing data will be imputed if the number of missing data is non-negligible or could potentially bias the results and conclusions.

Systematic review of prognostic factors

A systematic review of prognostic factors for ARCR outcomes is implemented (PROSPERO registration ID: CRD42020199257). Briefly, literature from 2014 to 2020 will be checked to

For peer review only - http://bmjopen.bmj.com/site/about/guidelines.xhtml

identify longitudinal studies including patients diagnosed with a RC tear. These studies should report the effect of at least one factor on one of the following outcomes: shoulder stiffness, RC tear repair integrity, and shoulder function. Data extraction will follow a pre-defined template and the collected data will be stored within a separate database using REDCap. Data from different studies will be described and may be synthesized depending on the data type and heterogeneity. These data will be used to generate a list of factors most likely to influence our project outcomes and therefore, should be considered for inclusion in the predictive model development process.

Predictive model development

To develop the predictive model(s), the seven steps proposed by Steyerberg et al. [73 74] will be used. The steps comprise: 1) consideration of the research question and initial data inspection, 2) coding of the predictors, 3) model specification, 4) model estimation, 5) evaluation of model performance, 6) internal validation, and 7) model presentation. Depending on the type of outcome, different models will be fitted and evaluated, i.e. multiple regression models for the change in OSS at 6 months and multivariable logistic regression models for shoulder stiffness. Model diagnostics will be performed for all models to check the underlying assumptions.

The prediction of the model(s) will be based on the baseline, operative, and postoperative management variables. Firstly, a subset of the potential prognostic factors will be defined based on whether it is thought to be most predictive. The subset will be selected separately for each outcome by the Delphi method among the investigators, whereby the factors will be noted for their known or potential prognostic value on a 5-point Likert scale from 1 (not important) to 5 (extremely important). These factors, with the highest mean score among investigators, will form the subset.

For peer review only - http://bmjopen.bmj.com/site/about/guidelines.xhtml

For peer review only

We will then use criterion-based procedures (e.g. Akaike Information Criterion [AIC] or adjusted R^2) to select the best set of predictors for the continuous outcome(s) and for the binary outcome, we will use the area under receiving operating characteristics curve (AUC). To assess the predictive performance of the final models as well as the updated version of the prediction models, the calibration plot and discrimination measures will be used. Thereby, apparent performance will be evaluated on the respective development data, and internal validated performance will be determined by bootstrapping. Independent external validation will be estimated by applying the resulting models from the development data set in the respective validation data sets. The resulting models will be used to predict the change of outcome value (i.e. OSS in 6 months) and assess whether a patient will experience the event (i.e. shoulder stiffness).

If we observe missing data, then missing data imputation will be performed using a method that allows for uncertainty in the imputed values (e.g. multiple imputations using chained equation [75]). We will account for the clustering of records within clinics as appropriate.

Adverse events

Occurring AEs other than those listed in the CES as well as events occurring outside the periods defined by the core set will be analyzed separately for consideration of clinical relevance. This analysis will be made by the review committee and project scientific board (PSB) comprising all local project leaders (principal investigators). Recommendations for change of the ARCR CES 1.0 by the PSB will be formulated.

The incidence of AEs, specific individual events and groups of events defined within the ARCR CES 1.0 up to 24 months postoperatively will be displayed as the frequency of patients with an event relative to the number of patients observed, reported together with its 95% Wilson confidence interval. These results will be presented in a summary table together with the absolute frequency. Further details on the period of occurrence will be given by

For peer review only - http://bmjopen.bmj.com/site/about/guidelines.xhtml

stratifying for the time point of event occurrence. We will also stratify AEs according to their severity level and patient relevance. Validation of the postoperative local AE severity classification system will be implemented using previously used methods [76 77].

Patient and public involvement

No patient or member of the public was involved in the design of this cohort study protocol. Enrolled patients will contribute to the evaluation and validation of documented AEs and their severity grading, therefore to a potential revision of the ARCR CES. We are planning to present initial results to patients and the public, and get feedback for further analyses and future model development as well as documentation system in ARCR.

Ethics and dissemination

Research ethics approval

Ethical approval was obtained on April 1st 2020 from the lead ethics committee (EKNZ, Basel Switzerland; ID: 2019-02076).

Protocol amendments

Minor protocol amendments, e.g. database production changes to facilitate monitoring processes or improve outcome assessment by questionnaire, are fully documented. Major amendments, e.g. changes to the patient information sheet and consent form, change of a local project leader or the inclusion of a new project site, will be submitted for approval by the lead ethics committee as required.

Consent or assent

All participants will provide informed written consent prior to being enrolled into the study. The English version of the informed consent form used at the University Hospital of Basel is available as Supplement file 1.

For peer review only - http://bmjopen.bmj.com/site/about/guidelines.xhtml

Confidentiality

Project data will be handled with utmost discretion and can only be accessed by authorized personnel as outlined by a study delegation list created for each project site. Patient data will be coded, i.e. identified by a unique participant number. A participant identification list will be managed and kept in a place (an electronic folder or paper-based form) only accessible to authorized staff at each site.

The project leader affirms and upholds the principle of each patient's right to privacy and that they shall comply with applicable privacy laws. In particular, anonymity of all patients shall be guaranteed when presenting the data at scientific meetings or publishing them in scientific journals.

Declaration of interests

None declared.

Access to data

Project data will be shared at the end of the analysis process by the PSB. The Department of Clinical Research (German Departement Klinische Forschung, DKF) at the University Hospital of Basel will act as an independent data access committee, and will store the data at the time of publication on secure servers, maintained and backed-up by the Information and Communication Technology department at the University Hospital of Basel. Researchers who wish to reuse data will be able to submit a project synopsis to the DKF at dkf.unibas.ch/contact. A data-sharing statement referring researchers to the DKF for data access will be disseminated in the publications. Metadata describing the type, size and content of the data sets will be shared along with the study protocol on the Harvard Dataverse repository available online (https://dataverse.harvard.edu/). Additionally, the case report forms will be uploaded on a medical data models portal (https://medical-data-models.org/) and all variables will be annotated by their Unified Medical Language System Concept Unique Identifier to improve accessibility to other clinicians.

For peer review only - http://bmjopen.bmj.com/site/about/guidelines.xhtml

Dissemination policy

This project will lead to multiple open-access, peer-reviewed scientific publications, which will be prepared according to international standards (e.g. the STROBE statement [78] for cohort studies; TRIPOD [79] statements for prognostic studies; PRISMA[80] statement for systematic reviews). Publication authorship will regulated according to the guidelines of the Swiss Academies of Arts and Sciences [81]. Results will be submitted for presentation at national and international conferences. In addition, lay summary results will be developed and made available for patients and the public.

Scientific relevance and broader impact

This project initiates the development of personalized risk predictions to support the surgical decision process in ARCR. The consensus CES may become an international reference for the reporting of complications in clinical studies and registries, and may therefore provide a solid metric for the documentation of surgical safety in ARCR. Methodological insight gained from this project will be easily transferable to similar initiatives and thus, may foster the realization of other cohorts on safety and effectiveness outcome in shoulder surgery (e.g. arthroplasty) and orthopedics in general.

For patients affected by RC tears and their surgeons, this study will be the first to provide solid data on the incidence of patient-validated AEs and other core outcomes up to two years after surgical repair based on international consensus COS and CES. This study will allow the investigation of a comprehensive list of potential prognostic factors to generate predictive models for these core outcomes and hence, offer personalized health information to support future patients and surgeons in the decision process for surgery. Outcome predictors and risk calculators are increasingly being developed in numerous medical fields including surgery and orthopedics, and they are in development in the field of ARCR.

For peer review only - http://bmjopen.bmj.com/site/about/guidelines.xhtml

This study will assess the structure and content of the ARCR CES and consolidate its validity in capturing unfavorable events of importance to both patients and surgeons; considering the patient's perspective is an essential step in the development of a COS. Furthermore, the validation of an adapted severity classification of AEs in this study will provide an essential system for assessing surgical morbidity in orthopedics. We expect that the ARCR CES and the event severity classification will become international standards for the reporting of ARCR AEs in clinical studies and registries, and therefore provide a solid metric for the documentation of surgical safety in ARCR.

This study fosters the enterprise in developing a Swiss-wide registry of ARCR, which will allow the ongoing evaluation and prediction of targeted core safety, clinical and patient-reported outcomes. The identification of factors mostly associated with relevant outcomes will facilitate a lean and straightforward documentation process for ARCR patients in Switzerland and abroad.

Acknowledgments

The authors acknowledge the support of Dr. Martina Gosteli, medical librarian at the University of Zurich, for implementing the preliminary literature database search to support this protocol development. The authors would also like to acknowledge the support of Dr. Melissa Wilhelmi, medical writer at Schulthess Klinik, Zurich, Switzerland, for manuscript proofreading.

For peer review only - http://bmjopen.bmj.com/site/about/guidelines.xhtml

References

1. Schmidt CC, Morrey BF. Management of full-thickness rotator cuff tears: appropriate use criteria. *J Shoulder Elbow Surg* 2015;24(12):1860-7. doi: 10.1016/j.jse.2015.05.042

2. Carr AJ, Cooper CD, Campbell MK, et al. Clinical effectiveness and cost-effectiveness of open and arthroscopic rotator cuff repair [the UK Rotator Cuff Surgery (UKUFF) randomised trial]. *Health Technol Assess* 2015;19(80):1-218. doi: 10.3310/hta19800 [published Online First: 2015/10/16]

3. Moosmayer S, Lund G, Seljom US, et al. At a 10-Year Follow-up, Tendon Repair Is Superior to Physiotherapy in the Treatment of Small and Medium-Sized Rotator Cuff Tears. *J Bone Joint Surg Am* 2019;101(12):1050-60. doi: 10.2106/JBJS.18.01373 [published Online First: 2019/06/21]

4. Grobet C, Audige L, Eichler K, et al. Cost-Utility Analysis of Arthroscopic Rotator Cuff Repair: A Prospective Health Economic Study Using Real-World Data. *Arthrosc Sports Med Rehabil* 2020;2(3):e193-e205. doi: 10.1016/j.asmr.2020.02.001 [published Online First: 2020/06/18]

5. Piper CC, Hughes AJ, Ma Y, et al. Operative versus nonoperative treatment for the management of full-thickness rotator cuff tears: a systematic review and meta-analysis. *J Shoulder Elbow Surg* 2018;27(3):572-76. doi: 10.1016/j.jse.2017.09.032 [published Online First: 2017/11/25]

6. Ensor KL, Kwon YW, Dibeneditto MR, et al. The rising incidence of rotator cuff repairs. *J Shoulder Elbow Surg* 2013;22(12):1628-32. doi: 10.1016/j.jse.2013.01.006

7. Iyengar JJ, Samagh SP, Schairer W, et al. Current trends in rotator cuff repair: surgical technique, setting, and cost. *Arthroscopy* 2014;30(3):284-8. doi: 10.1016/j.arthro.2013.11.018

8. Colvin AC, Egorova N, Harrison AK, et al. National trends in rotator cuff repair. *J Bone Joint Surg Am* 2012;94(3):227-33. doi: 10.2106/JBJS.J.00739

9. Jung C, Buchmann S, Flury M. Die arthroskopische Rotatorenmanschetten-Rekonstruktion - Von der Aussnahme zum Standard. Swiss Journal of Medicine and Medical Technology (Swiss Med), 2012:35-39.

10. Keener JD, Patterson BM, Orvets N, et al. Degenerative Rotator Cuff Tears: Refining Surgical Indications Based on Natural History Data. *J Am Acad Orthop Surg* 2019;27(5):156-65. doi: 10.5435/JAAOS-D-17-00480 [published Online First: 2018/10/20]

11. Randelli P, Spennacchio P, Ragone V, et al. Complications associated with arthroscopic rotator cuff repair: a literature review. *Musculoskelet Surg* 2012;96(1):9-16. doi: 10.1007/s12306-011-0175-y

For peer review only

For peer review only - http://bmjopen.bmj.com/site/about/guidelines.xhtml

12. Felsch Q, Mai V, Durchholz H, et al. Complications within 6 months after arthroscopic rotator cuff repair: registry-based evaluation according to a core event set and severity grading. *Arthroscopy* 2020:in Press. doi: 10.1016/j.arthro.2020.08.010 [published Online First: 2020/08/25]

13. Muller AM, Flury M, Alsayed HN, et al. Influence of patient and diagnostic parameters on reported retear rates after arthroscopic rotator cuff repair. *Knee Surg Sports Traumatol Arthrosc* 2017 doi: 10.1007/s00167-017-4481-2

14. Yoo JH, Cho NS, Rhee YG. Effect of postoperative repair integrity on health-related quality of life after rotator cuff repair: healed versus retear group. *Am J Sports Med* 2013;41(11):2637-44. doi: 10.1177/0363546513499152 [published Online First: 2013/08/15]

15. Robinson HA, Lam PH, Walton JR, et al. The effect of rotator cuff repair on early overhead shoulder function: a study in 1600 consecutive rotator cuff repairs. *Journal of Shoulder & Elbow Surgery* 2017;26(1):20-29. doi: https://dx.doi.org/10.1016/j.jse.2016.05.022

16. Huberty DP, Schoolfield JD, Brady PC, et al. Incidence and treatment of postoperative stiffness following arthroscopic rotator cuff repair. *Arthroscopy : the journal of arthroscopic & related surgery : official publication of the Arthroscopy Association of North America and the International Arthroscopy Association* 2009;25(8):880-90. doi: 10.1016/j.arthro.2009.01.018 [published Online First: 2009/08/12]

17. Denard PJ, Ladermann A, Burkhart SS. Prevention and management of stiffness after arthroscopic rotator cuff repair: systematic review and implications for rotator cuff healing. *Arthroscopy* 2011;27(6):842-8. doi: 10.1016/j.arthro.2011.01.013

18. Itoi E, Arce G, Bain GI, et al. Shoulder Stiffness: Current Concepts and Concerns. *Arthroscopy : the journal of arthroscopic & related surgery : official publication of the Arthroscopy Association of North America and the International Arthroscopy Association* 2016;32(7):1402-14. doi: 10.1016/j.arthro.2016.03.024 [published Online First: 2016/05/18]

19. Audige L, Blum R, Muller AM, et al. Complications Following Arthroscopic Rotator Cuff Tear Repair: A Systematic Review of Terms and Definitions With Focus on Shoulder Stiffness. *Orthopaedic Journal of Sports Medicine* 2015;3(6) doi: 10.1177/2325967115587861

20. Gargon E, Gurung B, Medley N, et al. Choosing important health outcomes for comparative effectiveness research: a systematic review. *PLoS One* 2014;9(6):e99111. doi: 10.1371/journal.pone.0099111

21. Buchbinder R, Page MJ, Huang H, et al. A Preliminary Core Domain Set for Clinical Trials of Shoulder Disorders: A Report from the OMERACT 2016 Shoulder Core

For peer review only - http://bmjopen.bmj.com/site/about/guidelines.xhtml

Outcome Set Special Interest Group. *J Rheumatol* 2017;44(12):1880-83. doi: 10.3899/jrheum.161123 [published Online First: 2017/01/17]

22. Page MJ, Huang H, Verhagen AP, et al. Identifying a core set of outcome domains to measure in clinical trials for shoulder disorders: a modified Delphi study. *RMD Open* 2016;2(2):e000380. doi: 10.1136/rmdopen-2016-000380 [published Online First: 2017/01/27]
23. Audige L, Flury M, Muller AM, et al. Complications associated with arthroscopic rotator cuff tear repair: definition of a core event set by Delphi consensus process. *J Shoulder Elbow Surg* 2016;25(12):1907-17. doi: 10.1016/j.jse.2016.04.036
24. Felsch Q, Mai V, Durchholz H, et al. Complications within 6 months after arthroscopic rotator cuff repair: registry-based evaluation according to a core event set and severity grading. *Arthroscopy : the journal of arthroscopic & related surgery : official publication of the Arthroscopy Association of North America and the International Arthroscopy Association* 2020 doi: 10.1016/j.arthro.2020.08.010 [published Online First: 2020/08/25]
25. Steyerberg EW, Moons KG, van der Windt DA, et al. Prognosis Research Strategy (PROGRESS) 3: prognostic model research. *PLoS Med* 2013;10(2):e1001381. doi: 10.1371/journal.pmed.1001381 [published Online First: 2013/02/09]
26. Sepucha KR, Atlas SJ, Chang Y, et al. Informed, Patient-Centered Decisions Associated with Better Health Outcomes in Orthopedics: Prospective Cohort Study. *Med Decis Making* 2018;38(8):1018-26. doi: 10.1177/0272989X18801308 [published Online First: 2018/11/08]
27. Guo W, Jin J, Paynabar K, et al. A decision support system on surgical treatments for rotator cuff tears. *IIE Transactions on Healthcare Systems Engineering* 2015;5(3):197-210. doi: 10.1080/19488300.2015.1065935
28. Kwon J, Kim SH, Lee YH, et al. The Rotator Cuff Healing Index: A New Scoring System to Predict Rotator Cuff Healing After Surgical Repair. *Am J Sports Med* 2019;47(1):173-80. doi: 10.1177/0363546518810763 [published Online First: 2018/11/30]
29. Jeong HY, Kim HJ, Jeon YS, et al. Factors predictive of healing in large rotator cuff tears: Is it possible to predict retear preoperatively? *The American journal of sports medicine* 2018;46(7):1693-700.
30. Frangiamore S, Dornan GJ, Horan MP, et al. Predictive Modeling to Determine Functional Outcomes After Arthroscopic Rotator Cuff Repair. *Am J Sports Med* 2020;48(7):1559-67. doi: 10.1177/0363546520914632 [published Online First: 2020/05/15]

For peer review only

31. Koorevaar RC, van't Riet E, Ipskamp M, et al. Incidence and prognostic factors for postoperative frozen shoulder after shoulder surgery: a prospective cohort study. *Archives of orthopaedic and trauma surgery* 2017;137(3):293-301.

32. Fermont AJ, Wolterbeek N, Wessel RN, et al. Prognostic factors for recovery after arthroscopic rotator cuff repair: a prognostic study. *J Shoulder Elbow Surg* 2015;24(8):1249-56. doi: 10.1016/j.jse.2015.04.013

33. Lambers Heerspink FO, Dorrestijn O, van Raay JJ, et al. Specific patient-related prognostic factors for rotator cuff repair: a systematic review. *J Shoulder Elbow Surg* 2014;23(7):1073-80. doi: 10.1016/j.jse.2014.01.001

34. McElvany MD, McGoldrick E, Gee AO, et al. Rotator cuff repair: published evidence on factors associated with repair integrity and clinical outcome. *American Journal of Sports Medicine* 2015;43(2):491-500. doi: http://dx.doi.org/10.1177/0363546514529644

35. Saccomanno MF, Sircana G, Cazzato G, et al. Prognostic factors influencing the outcome of rotator cuff repair: a systematic review. *Knee Surg Sports Traumatol Arthrosc* 2016;24(12):3809-19.

36. Raman J, Walton D, MacDermid JC, et al. Predictors of outcomes after rotator cuff repair-A meta-analysis. *J Hand Ther* 2017;30(3):276-92. doi: https://dx.doi.org/10.1016/j.jht.2016.11.002

37. Dindo D, Demartines N, Clavien PA. Classification of surgical complications: a new proposal with evaluation in a cohort of 6336 patients and results of a survey. *Ann Surg* 2004;240(2):205-13. [published Online First: 2004/07/27]

38. Lafosse L, Jost B, Reiland Y, et al. Structural integrity and clinical outcomes after arthroscopic repair of isolated subscapularis tears. *J Bone Joint Surg Am* 2007;89(6):1184-93. doi: 10.2106/JBJS.F.00007

39. Dawson J, Rogers K, Fitzpatrick R, et al. The Oxford shoulder score revisited. *Arch Orthop Trauma Surg* 2009;129(1):119-23. doi: 10.1007/s00402-007-0549-7 [published Online First: 2008/01/10]

40. Huber W, Hofstaetter JG, Hanslik-Schnabel B, et al. The German version of the Oxford Shoulder Score--cross-cultural adaptation and validation. *Arch Orthop Trauma Surg* 2004;124(8):531-6.

41. Murena L, Vulcano E, D'Angelo F, et al. Italian cross-cultural adaptation and validation of the Oxford Shoulder Score. *J Shoulder Elbow Surg* 2010;19(3):335-41. doi: S1058-2746(09)00376-0 [pii]

10.1016/j.jse.2009.07.068 [published Online First: 2009/11/10]

For peer review only

42. Olley LM, Carr AJ. The use of a patient-based questionnaire (the Oxford Shoulder Score) to assess outcome after rotator cuff repair. *Ann R Coll Surg Engl* 2008;90(4):326-31. doi: 10.1308/003588408X285964 [published Online First: 2008/05/22]
43. Xu S, Chen JY, Lie HME, et al. Minimal Clinically Important Difference of Oxford, Constant, and UCLA shoulder score for arthroscopic rotator cuff repair. *J* 2020;19:21-27. doi: 10.1016/j.jor.2019.11.037 [published Online First: 2020/02/06]
44. Allom R, Colegate-Stone T, Gee M, et al. Outcome analysis of surgery for disorders of the rotator cuff: a comparison of subjective and objective scoring tools. *J Bone Joint Surg Br* 2009;91(3):367-73. doi: 10.1302/0301-620X.91B3.20714 [published Online First: 2009/03/05]
45. Constant CR, Gerber C, Emery RJ, et al. A review of the Constant score: modifications and guidelines for its use. *J Shoulder Elbow Surg* 2008;17(2):355-61. doi: 10.1016/j.jse.2007.06.022 [published Online First: 2008/01/26]
46. Gilbart MK, Gerber C. Comparison of the subjective shoulder value and the Constant score. *J Shoulder Elbow Surg* 2007;16(6):717-21. doi: 10.1016/j.jse.2007.02.123 [published Online First: 2007/12/07]
47. Cappelleri JC, Bushmakin AG, McDermott AM, et al. Psychometric properties of a single-item scale to assess sleep quality among individuals with fibromyalgia. *Health and quality of life outcomes* 2009;7(1):54.
48. Potter MQ, Wylie JD, Greis PE, et al. Psychological distress negatively affects self-assessment of shoulder function in patients with rotator cuff tears. *Clin Orthop Relat Res* 2014;472(12):3926-32. doi: 10.1007/s11999-014-3833-1
49. Makhni EC, Meadows M, Hamamoto JT, et al. Patient Reported Outcomes Measurement Information System (PROMIS) in the upper extremity: the future of outcomes reporting? *Journal of Shoulder and Elbow Surgery* 2017;26(2):352-57. doi: 10.1016/j.jse.2016.09.054
50. Tubach F, Wells GA, Ravaud P, et al. Minimal clinically important difference, low disease activity state, and patient acceptable symptom state: methodological issues. *J Rheumatol* 2005;32(10):2025-9. [published Online First: 2005/10/06]
51. Slankamenac K, Graf R, Barkun J, et al. The comprehensive complication index: a novel continuous scale to measure surgical morbidity. *Ann Surg* 2013;258(1):1-7. doi: 10.1097/SLA.0b013e318296c732
52. Sugaya H, Maeda K, Matsuki K, et al. Functional and structural outcome after arthroscopic full-thickness rotator cuff repair: single-row versus dual-row fixation. *Arthroscopy : the journal of arthroscopic & related surgery : official publication of the Arthroscopy Association of North America and the International Arthroscopy Association* 2005;21(11):1307-16. doi: 10.1016/j.arthro.2005.08.011 [published Online First: 2005/12/06]

For peer review only

For peer review only - http://bmjopen.bmj.com/site/about/guidelines.xhtml

53. Collin P, Yoshida M, Delarue A, et al. Evaluating postoperative rotator cuff healing: Prospective comparison of MRI and ultrasound. *Orthop Traumatol Surg Res* 2015;101(6 Suppl):S265-8. doi: 10.1016/j.otsr.2015.06.006 [published Online First: 2015/08/19]

54. Herdman M, Gudex C, Lloyd A, et al. Development and preliminary testing of the new five-level version of EQ-5D (EQ-5D-5L). *Qual Life Res* 2011;20(10):1727-36. doi: 10.1007/s11136-011-9903-x [published Online First: 2011/04/12]

55. Moor BK, Wieser K, Slankamenac K, et al. Relationship of individual scapular anatomy and degenerative rotator cuff tears. *J Shoulder Elbow Surg* 2014;23(4):536-41. doi: 10.1016/j.jse.2013.11.008

56. Werner CM, Conrad SJ, Meyer DC, et al. Intermethod agreement and interobserver correlation of radiologic acromiohumeral distance measurements. *J Shoulder Elbow Surg* 2008;17(2):237-40. doi: 10.1016/j.jse.2007.06.002

57. Thomazeau H, Rolland Y, Lucas C, et al. Atrophy of the supraspinatus belly assessment by MRI in 55 patients with rotator cuff pathology. *Acta orthopaedica Scandinavica* 1996;67(3):264-68.

58. Zanetti M, Gerber C, Hodler J. Quantitative assessment of the muscles of the rotator cuff with magnetic resonance imaging. *Investigative radiology* 1998;33(3):163-70.

59. Schiefer M, Mendonca R, Magnanini MM, et al. Intraobserver and interobserver agreement of Goutallier classification applied to magnetic resonance images. *J Shoulder Elbow Surg* 2015;24(8):1314-21. doi: 10.1016/j.jse.2015.02.011

60. Goutallier D, Postel JM, Bernageau J, et al. Fatty muscle degeneration in cuff ruptures. Pre- and postoperative evaluation by CT scan. *Clin Orthop Relat Res* 1994(304):78-83. [published Online First: 1994/07/01]

61. Patte D. Classification of rotator cuff lesions. *Clin Orthop Relat Res* 1990(254):81-6. [published Online First: 1990/05/01]

62. Okoroha KR, Mehran N, Duncan J, et al. Characterization of Rotator Cuff Tears: Ultrasound Versus Magnetic Resonance Imaging. *Orthopedics* 2016:1-7. doi: 10.3928/01477447-20161013-04

63. Zumstein MA, Ladermann A, Raniga S, et al. The biology of rotator cuff healing. *Orthopaedics & traumatology, surgery & research : OTSR* 2016 doi: 10.1016/j.otsr.2016.11.003 [published Online First: 2017/01/04]

64. Audige L, Goldhahn S, Daigl M, et al. How to document and report orthopedic complications in clinical studies? A proposal for standardization. *Arch Orthop Trauma Surg* 2014;134(2):269-75. doi: 10.1007/s00402-011-1384-4

65. Rosenthal R, Hoffmann H, Clavien PA, et al. Definition and Classification of Intraoperative Complications (CLASSIC): Delphi Study and Pilot Evaluation. *World J Surg* 2015;39(7):1663-71. doi: 10.1007/s00268-015-3003-y

For peer review only

66. Dell-Kuster S, Gomes NV, Gawria L, et al. Prospective validation of classification of intraoperative adverse events (ClassIntra): international, multicentre cohort study. *BMJ* 2020;370:m2917. doi: 10.1136/bmj.m2917 [published Online First: 2020/08/28]

67. Flury M, Kolling C, Grobet C, et al. Implementation of a local outcome register for arthroscopic rotator cuff tear repair. *Obere Extremität* 2014;10(1):33-40. doi: 10.1007/s11678-014-0299-4

68. Vach W. Regression models as a tool in medical research. Boca Raton, FL: CRC Press, Taylor & Francis Group 2013.

69. Peduzzi P, Concato J, Kemper E, et al. A simulation study of the number of events per variable in logistic regression analysis. *J Clin Epidemiol* 1996;49(12):1373-9. [published Online First: 1996/12/01]

70. Hemingway H, Croft P, Perel P, et al. Prognosis research strategy (PROGRESS) 1: a framework for researching clinical outcomes. *BMJ* 2013;346:e5595. doi: 10.1136/bmj.e5595 [published Online First: 2013/02/07]

71. Harris PA, Taylor R, Thielke R, et al. Research electronic data capture (REDCap)--a metadata-driven methodology and workflow process for providing translational research informatics support. *Journal of biomedical informatics* 2009;42(2):377-81. doi: 10.1016/j.jbi.2008.08.010 [published Online First: 2008/10/22]

72. REDCap web-based Electronic Data Capture system 2017 [Available from: www.project-redcap.org accessed 15.03.2017 2017.

73. Steyerberg EW, Vergouwe Y. Towards better clinical prediction models: seven steps for development and an ABCD for validation. *European Heart Journal* 2014;35(29):1925-31. doi: 10.1093/eurheartj/ehu207

74. Steyerberg EW. Clinical prediction models, 2009.

75. Team RC. R: A language and environment for statistical computing. : R Foundation for Statistical Computing, Vienna, Austria; 2016 [Available from: www.R-project.org.

76. Porembka MR, Hall BL, Hirbe M, et al. Quantitative weighting of postoperative complications based on the accordion severity grading system: demonstration of potential impact using the american college of surgeons national surgical quality improvement program. *Journal of the American College of Surgeons* 2010;210(3):286-98. doi: 10.1016/j.jamcollsurg.2009.12.004

77. de la Rosette JJ, Opondo D, Daels FP, et al. Categorisation of complications and validation of the Clavien score for percutaneous nephrolithotomy. *European urology* 2012;62(2):246-55.

78. Vandenbroucke JP, Von Elm E, Altman DG, et al. Strengthening the Reporting of Observational Studies in Epidemiology (STROBE): explanation and elaboration. *PLoS Med* 2007;4(10):e297.

For peer review only

For peer review only - http://bmjopen.bmj.com/site/about/guidelines.xhtml

79. Collins GS, Reitsma JB, Altman DG, et al. Transparent Reporting of a multivariable prediction model for Individual Prognosis or Diagnosis (TRIPOD): the TRIPOD statement. *Ann Intern Med* 2015;162(1):55-63. doi: 10.7326/M14-0697 [published Online First: 2015/01/07]

80. Moher D, Liberati A, Tetzlaff J, et al. Preferred reporting items for systematic reviews and meta-analyses: the PRISMA statement. *PLoS Med* 2009;6(7):e1000097. doi: 10.1371/journal.pmed.1000097 [published Online First: 2009/07/22]

81. Swiss Academies of Arts and Sciences. Authorship in scientific publications – Analysis and recommendations [Available from: http://www.swiss-academies.ch/en/index/Publikationen/Archiv/Richtlinien-Empfehlungen.html accessed 06.10.2020.

For peer review only

916 **Figure legend**

917

918 **Figure 1**: Flowchart of study procedures

919 w = week; mo = month; FU = follow-up; MRI = Magnetic Resonance Imaging; CT = Arthro-

920 Computer Tomography (*if MRI not possible); Motion = Shoulder range of motion; CS =

921 Constant Score; RC = Rotator Cuff; AE = Adverse Event; Rehab. = recall on postoperative

922 rehabilitation; DE = German; FR = French; IT = Italian; EN = English; NRS = Numeric Rating

923 Scale; OSS = Oxford Shoulder Score; SSV = Subjective Shoulder Value; EQ-5D-5L =

924 European Quality of Life 5 Dimensions 5 Level questionnaire; CES = Core Event Set; AE

925 survey = surgeons and patients survey regarding AE severity (sev)

926

For peer review only

For peer review only - http://bmjopen.bmj.com/site/about/guidelines.xhtml

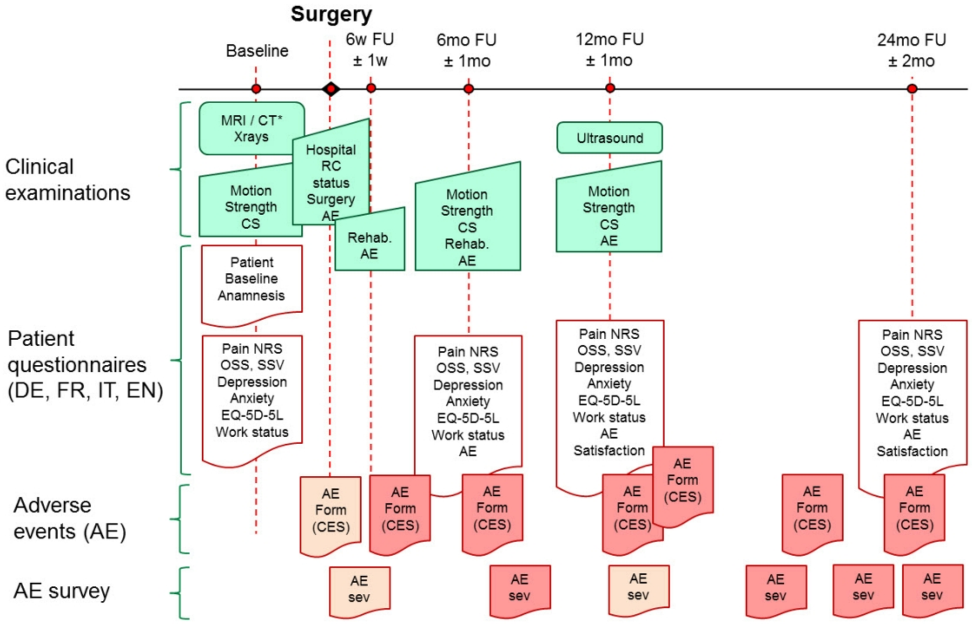

Figure 1: Flowchart of study procedures
w = week; mo = month; FU = follow-up; MRI = Magnetic Resonance Imaging; CT = Arthro-Computer Tomography (*if MRI not possible); Motion = Shoulder range of motion; CS = Constant Score; RC = Rotator Cuff; AE = Adverse Event; Rehab. = recall on postoperative rehabilitation; DE = German; FR = French; IT = Italian; EN = English; NRS = Numeric Rating Scale; OSS = Oxford Shoulder Score; SSV = Subjective Shoulder Value; EQ-5D-5L = European Quality of Life 5 Dimensions 5 Level questionnaire; CES = Core Event Set; AE survey = surgeons and patients survey regarding AE severity (sev)

342x220mm (96 x 96 DPI)

Supplement file 1

**Article title**	Protocol for the ARCR_Pred cohort-study: Swiss-wide multicenter evaluation and prediction of core outcomes in arthroscopic rotator cuff repair
**Journal name**	BMJ Open Access
**Author names**	Laurent Audigé, Heiner C. Bucher, Soheila Aghlmandi, Thomas Stojanov, David Schwappach, Sabina Hunziker, Christian Candrian, Gregory Cunningham, Holger Durchholz, Karim Eid, Matthias Flury, Bernhard Jost, Alexandre Lädermann, Beat Moor, Philipp Moroder, Claudio Rosso, Michael Schär, Markus Scheibel, Christoph Spormann, Thomas Suter, Karl Wieser, Matthias A. Zumstein, ARCR_Pred Study Group*, Andreas Müller
**Corresponding author**	Laurent Audigé, Schulthess Klinik, CH-8008 Zurich, Switzerland
**e-mail address**	laurent.audige@kws.ch

* Members of the ARCR_Pred study group are listed in the main publication of this project protocol.

Patient information sheet and informed consent form (English version) for the project site “University Hospital of Basel” (USB)

For peer review only

Universitätsspital Basel

Engl. Surgical safety and effectiveness in orthopaedics: evaluation of an international consensus core set of adverse events in arthroscopic rotator cuff repair

This project has been organised by: PD Dr. Andreas Müller (project leader; Deputy Consultant of Orthopaedics, Shoulder/Elbow team leader, Orthopaedic and Traumatology Department, University Hospital of Basel) and Prof. Dr. Laurent Audigé (project manager; Research Associate, Orthopaedic and Traumatology Department, University Hospital of Basel and Head of Research Group Upper Extremities, Department of Research and Development, Schulthess Clinic, Zurich)

Sponsor: University Hospital of Basel, PD Dr. Andreas Müller, Deputy Consultant of Orthopaedics and Traumatology

Dear Sir / Madam,

We would like to ask you if you are interested in participating in a research project. The planned project is first presented as a short summary in the table provided below followed by a more detailed description.

Summary of the project

1	**Aim of the project**<br>The research project will investigate how easily the most important results are predicted following surgical interventions to treat tendinosis (= rotator cuff tears) of the shoulder muscles. In order to do this, we will use and assess a predefined set of adverse events (such as frozen shoulder or persistent pain) in practice.
2	**Choice**<br>You are an adult suffering from tendinosis of the shoulder muscles, which can be treated with an initial, minimally invasive (i.e. arthroscopic) surgical intervention. That is why we are sending you this information leaflet.
3	**General information about the project**<br>The collection of clinical data following surgical interventions is very important, and helps to support an established decision-making process within the orthopaedic field. We are carrying out this project so that we can better evaluate and predict the results following the repair of shoulder tendinosis. This evaluation is being carried out on a representative number of male and female patients in Switzerland.<br>Patient safety is essential here. A list of possible adverse events following the arthroscopic intervention was recently defined by a group of more than 80 experts in the field. We would now like to better understand the development of these adverse events from the patient's point of view.
4	**Procedure**<br>This project will last four years. In total, 970 patients from various clinics in Switzerland and one in Germany will be included in the study within the first year. Various examinations (e.g. measurements of range of motion and strength) will be carried out in the clinic before the operation (= preoperative) and then at the 6- and 12-month postoperative time points. At these times and at 2 years post-surgery, you will receive a questionnaire to complete. We will use ultrasound to check how the tendon is healing twelve months after the operation. Any adverse events will be evaluated independently by the doctor and the patient in question.

For peer review only - http://bmjopen.bmj.com/site/about/guidelines.xhtml

Universitätsspital Basel

5	**Usefulness**<br>You will gain no personal benefit from participating in the project. However, the results could be important to others who have the same condition.
6	**Rights**<br>You decide voluntarily whether you want to participate in this project or not. Your decision does not affect your medical treatment/care and you do not have to justify it.
7	**Duties**<br>If you participate, we ask you to adhere to certain requirements (e.g. attending visits and completing the questionnaires).
8	**Risks**<br>You are not exposed to any additional risk by participating in the project.
9	**Outcomes**<br>You will be informed of new results during the project. We will promptly inform you of any additional findings (known as incidental findings) that are detected during the regular study examinations, which may affect your health directly. Any further course of action will then be discussed in detail with you.
10	**Confidentiality of data and samples**<br>We collect your personal and medical data from you. The Swiss National Science Foundation supports the exchange and reuse of research data. The data will be used for other projects if you give your separate consent. We comply with all legal data protection regulations. All parties involved are bound by confidentiality.
11	**Withdrawal**<br>You can withdraw from the project at any time and no longer participate. The data collected so far are still being evaluated.
12	**Indemnity**<br>You will not receive any compensation for participating in the study. Neither your health insurance provider nor you will incur any additional costs from your participation in the study.
13	**Liability**<br>The liability insurance of the project management is liable for any damages within the scope of the project.
14	**Funding**<br>The project is paid for by the Swiss National Science Foundation.
15	**Contact person:**<br>You can receive information on all your questions at any time:<br><br>PD Dr. Andreas Müller, Senior Consultant, Head Shoulder and Elbow,<br>University Hospital of Basel, Spitalstrasse 21, CH-403 Basel<br>Tel 061 315 25 17 , Email A.Mueller@usb.ch

For peer review only - http://bmjopen.bmj.com/site/about/guidelines.xhtml

Universitätsspital Basel

More detailed information

1. Aim of the project

The aim of this project is to investigate how the most important results (for example, the occurrence of adverse events or shoulder function) are easily predicted following a surgical intervention to treat tendinosis of the shoulder muscles. Furthermore, we want to investigate how well a predefined set of adverse events, which could occur as part of such a surgical intervention, corresponds to what actually occurs in practice.

2. Choice

Participation is open to anyone with a torn tendon in the shoulder muscles, which can be repaired with minimally invasive (i.e. arthroscopic) surgery.

Important: This must be the first intervention on the shoulder in question.

Participation is not open to anyone for whom a detailed medical examination would not be possible or who cannot be called back for check-ups within the follow-up period (e.g. if they live outside Switzerland). Underage persons should also not participate.

3. General information about the project

This project will be carried out in accordance with the laws of Switzerland. The responsible ethics committee has reviewed and approved this project

The collection of clinical data following surgical interventions is very important. You will help:

- to assess the effectiveness and safety of the intervention,
- to make comparisons with other methods,
- to support an established decision-making process within the orthopaedic field.

The study intends to document the most important events following arthroscopic interventions, especially in terms of:

- safety (occurrence of adverse events),
- healing of the tendon repair,
- shoulder pain and function,
- the general state of health and quality of life as well as
- patient satisfaction.

Patients' socio-demographic characteristics, examination parameters and treatment parameters will be investigated to enable a prediction of these events to be made.

In most areas of orthopaedics, there is currently no international standard for the description of adverse events (often known as complications) resulting from surgical interventions. In shoulder orthopaedics, such a list of events does not exist for arthroscopic interventions used in the treatment of rotator cuff tears (shoulder tendinosis). For this reason, the Shoulder and Elbow Surgery research group at the Schulthess Clinic, Zurich, working together with the Orthopaedic and Traumatology Department of the University Hospital of Basel and over 80 experts working in the field of arthroscopy for rotator cuff tears, have defined a list of possible adverse events.

The aim is to apply and evaluate this predefined list of events. Each event will be evaluated independently by the doctor and the patient in question.

For peer review only - http://bmjopen.bmj.com/site/about/guidelines.xhtml

Universitätsspital Basel

This project is supported by the Swiss National Science Foundation and will last four years. In total, 970 patients will be included in the study within the first year. These patients will be recruited from various clinics in Switzerland and one clinic in Germany. At the University Hospital of Basel about 60 patients are recruited.

4. Procedure

The following diagram shows the course of study events from the time of diagnosis to the follow-up appointment at two years post-surgery.

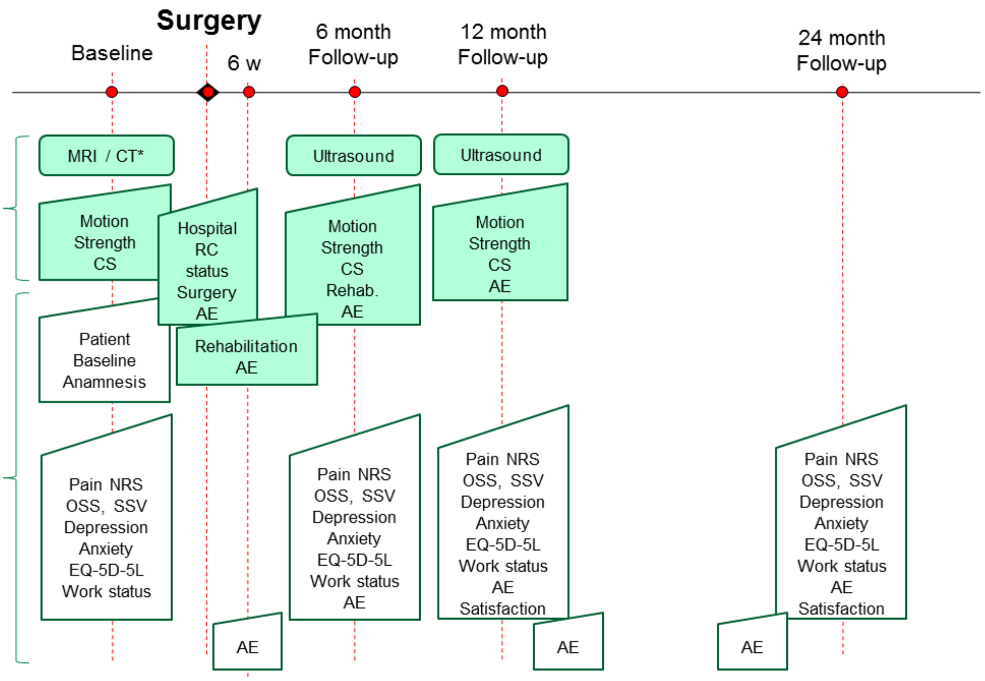

6 W = 6-week follow-up; AE = adverse events

Figure 1: Schematic representation of the study's progress

For your diagnosis, you will be examined by the doctor using various methods (e.g. functional testing, radiological and magnetic resonance imaging (= MRI)) during the first consultation. If you agree to participate in the project, you will receive a questionnaire, which will ask you to provide your personal details and estimate your current level of functional ability in everyday life. Shortly before the operation, your surgeon will record detailed information about your shoulder injury and the surgical intervention. Six weeks after the operation, your surgeon will ask you about your pain levels, rehabilitation programme and the occurrence of any adverse events.

During further follow-up appointments (at 6 and 12 months post-surgery), various examinations (e.g. measurements of range of motion and strength, see figure 1) are routinely performed in the clinic. An independent examiner will carry out an ultrasound routinely 12 months after the operation to document how the tendon is healing. You will also receive a questionnaire to complete. If you wish, you can complete this questionnaire at home - either on paper or electronically by following an invitation link that will be emailed to you. At the end of this document, you will be asked to provide your email address, if you do prefer to receive the electronic version of the questionnaire. Various questionnaires are already routinely recorded at the University Hospital of Basel.

For peer review only - http://bmjopen.bmj.com/site/about/guidelines.xhtml

Universitätsspital Basel

Two years after the operation, you will be sent the questionnaire again by post or email (no further check-ups will take place at the clinic). This takes place within the framework of the project. If you have experienced one or more adverse events, the questionnaire will ask you to rate each event by severity and by its relationship to your treatment. The study doctors will evaluate all reported events by severity without knowing the patient, to whom these relate or the clinic in which the events occurred.

Each visit to the clinic (before surgery and 6- and 12-months post-surgery) will last 40 to 60 minutes including all the examinations. Completing the patient questionnaire will take an additional 20 to 30 minutes. An adverse event tends to be evaluated in only a few minutes.

We may have to exclude you from this project prematurely. This can occur if no tear of the rotator cuff can be confirmed intraoperatively (i.e. during the operation) (this occurs very rarely) or if a tendon repair is not possible without additional interventions on the shoulder (a so-called irreparable tear). The study doctor will inform you of any such exclusion from the project.

5. Usefulness

You will not personally benefit from participating in the project. The results can be important for others who have the same condition. As described above, the study aims to enable better evaluation and prediction of the risk of adverse events and the effectiveness of a surgical intervention. We want to support the decision-making processes of future interventions of this kind.

6. Rights

You're volunteering. If you do not want to participate or later withdraw your participation, you do not have to justify this. Your medical treatment/care is guaranteed regardless of your decision. You may ask questions about participation and the project at any time. Please contact the person named at the end of this information.

7. Duties

As a participant, it is necessary that you

- adhere to the necessary specifications and requirements of the project management.
- inform your investigator/project management about the course of the disease and report new symptoms, new complaints and changes in well-being.
- inform your investigator/project management about the simultaneous treatment and therapy with another doctor and about taking medication.

8. Risks

You are not exposed to any additional risk by participating in the project.

9. Outcomes

The investigator/project manager will inform you during the project about any new findings that may affect the benefit or your safety and thus your consent to participate. You will be informed of random findings which may contribute to the prevention, detection or treatment of existing or future diseases.

10. Confidentiality of data and samples

Your personal and medical data will be collected for this project. Very few professionals will see your unencrypted data, and only to perform tasks within the scope of the project. Data collection for study purposes is encrypted. Encryption means that all reference data that could identify you (name, date of birth) are deleted and replaced by a key. The key list always remains in the institution/hospital. Those who do not know the key cannot therefore draw any conclusions about you. In the

For peer review only - http://bmjopen.bmj.com/site/about/guidelines.xhtml

Universitätsspital Basel

case of a publication, the summarised data cannot therefore be traced back to you as an individual. Your name will never appear on the Internet or in any publication. Sometimes there is a requirement in a journal for publication that individual data (so-called raw data) must be transmitted. If individual data must be transmitted, then the data is always encrypted and cannot be traced back to you as a person. All persons who have access to your data within the scope of the project are subject to confidentiality. The requirements of data protection are adhered to and you as a participating person have the right to inspect your data at any time.

If data are stored on site, it is a database for research purposes.

Each centre will encode and save the data centrally in the project database (server location: Schulthess Clinic, Zurich). The data will be deleted 10 years after the end of the project.
It is possible that your data may be used for other investigations (projects) at a later date or that they may be sent to another databank in Switzerland for investigations (further use) not yet defined in more detail. This other database must meet the same standards as the database for this project. For this further use we ask you to sign a further declaration of consent at the very end of this document.

This project may be reviewed by the relevant ethics committee or by the institution that initiated the project. The project manager may need to disclose your personal and medical information for such checks. All persons must maintain absolute confidentiality. We comply with all data protection regulations and will not make your name public either in a publication or on the Internet.

It is possible that your aftercare physician will be contacted to provide information about your medical condition.

11. Withdrawal
You can stop at any time and withdraw from the project if you wish. The data collected so far are still evaluated in encrypted form, otherwise the entire project loses its value. It is not possible to anonymize your data in case of withdrawal, i.e. the data remain encrypted. Please check whether you agree with this before you participate in the project.

12. Indemnity
If you participate in this project, you will not receive any compensation. You or your health insurance company will not incur any costs for participation.

13. Liability
The prerequisites and procedure relating to liability and safeguarding in the event of a claim are legally regulated. If you suffer a health impairment as a result of the study, please contact the study doctor. The institution that is responsible for carrying out the study is liable for the claim, if you can prove that the injury is due to the project-specific examinations. Liability will not be accepted if the project manager can prove that the injury is only minor and temporary, and does not extend beyond the degree expected by current scientific knowledge.

14. Funding
The project is being funded by the Swiss National Science Foundation (SNSF).

15. Contact person(s)
If you have any questions, concerns, or emergencies that arise during or after the project, you can always contact one of these contacts.

For peer review only - http://bmjopen.bmj.com/site/about/guidelines.xhtml

Universitätsspital Basel

Head at the study location:
PD Dr. Andreas Müller, Senior Consultant, Head Shoulder and Elbow
University Hospital of Basel, Spitalstrasse 21, CH-403 Basel
Tel 061 315 25 17 , Email A.Mueller@usb.ch

24-hour emergency number: +41 61 265 25 25

Local project coordination:

PD Dr. Andreas Müller, Senior Consultant, Head Shoulder and Elbow
University Hospital of Basel, Spitalstrasse 21, CH-403 Basel
Tel 061 315 25 17 , Email A.Mueller@usb.ch

For peer review only

For peer review only - http://bmjopen.bmj.com/site/about/guidelines.xhtml

Universitätsspital Basel

Declaration of consent

Written declaration of consent for participation in a study project
Please read this form carefully. Please ask if you do not understand or want to know something

**BASEC number (after submission):**	
**Title of the project (scientific and lay):**	Surgical safety and effectiveness in orthopaedics: Swiss-wide multicenter evaluation and prediction of core outcomes in arthroscopic rotator cuff reconstruction<br><br>Surgical safeguarding and effectiveness in orthopaedics: Swiss-wide multicentre evaluation and prediction of the most important effects following arthroscopic repair of shoulder tendons (rotator cuff reconstruction)
**Responsible institution (Project management with address):**	University Hospital of Basel<br>PD Dr. Andreas Müller<br>Orthopaedics and Traumatology<br>Spitalstrasse 21, CH-4031 Basel
**Place of implementation:**	Universitätsspital Basel
**Head of the project at the place of study:**	PD Dr. Andreas Müller
**Participant:**	Name, first name: ____________________<br><br>Date of Birth: ____________________<br><br>☐ Female ☐ Male

The undersigned investigator informed me verbally and in writing about the purpose, the course of the project, about possible advantages and disadvantages as well as about possible risks.

- I voluntarily participate in this project and accept the content of the written information provided on the above mentioned project. I've had plenty of time to make my decision.
- My questions concerning the participation in this project have been answered. I keep the written information and receive a copy of my written consent.
- I agree that the responsible experts of the project management/client of the project and the ethics committee responsible for this project may inspect my unencrypted data for verification and control purposes, but in strict compliance with confidentiality.
- I will be informed of study results or random findings that directly affect my health. If I don't want that, I'll inform my investigator.
- I know that my health-related and personal data can only be passed on in encrypted form for research purposes **for this project**.

For peer review only - http://bmjopen.bmj.com/site/about/guidelines.xhtml

Universitätsspital Basel

- In the event of further treatment outside the test centre, I authorise my after-treating doctor(s) to forward my after-treatment data relevant to the project to the investigator/project management.
- I can withdraw from participation at any time and without giving reasons, without having any disadvantages in further medical treatment/care. The data collected so far will still be used for the evaluation of the project.
- The liability insurance of the hospital/institution covers any damages.
- I am aware that the obligations stated in the participant information must be complied with.

- If you agree that your email address can be used solely for receiving questionnaires and project-related communications, please enter it here:

______________________________@______________________

Place, Date	Signature of participant

Confirmation from the investigator: I hereby confirm that I have explained the nature, significance and scope of the project to this participant. I assure you that I will fulfil all obligations in connection with this project in accordance with applicable law. If, at any time during the implementation of the project, I become aware of any aspects that might affect the participant's willingness to participate in the project, I will inform the participant immediately.

Place, Date	Name and first name of the informing investigator in block capitals<br><br>Signature of the investigating physician

For peer review only

For peer review only - http://bmjopen.bmj.com/site/about/guidelines.xhtml

Universitätsspital Basel

Declaration of consent for the further use of data in encrypted form.
(for further use of data of THIS project)

**Participant:**	Name, first name:______________________<br>Date of birth: __________________________<br>☐ Female ☐ Male

I allow my data from this project to be used in encrypted form for medical research. This means that the data may be stored in a databank and used for future, not yet defined research projects for an indefinite period of time. This consent is unlimited.

I decide voluntarily and can revoke this decision at any time. When I step back, my data is anonymized. I simply inform my investigator/project manager and do not have to justify this decision.

I understand that the data are encrypted and the code is kept safe. The data can be sent to other databanks in Switzerland and abroad for analysis if they comply with the same standards as in Switzerland. All legal requirements regarding data protection are complied with.

Normally, all data are evaluated in their entirety and the results published in summary form. Should a result be relevant for me, it is possible that I will be contacted via my investigator. If I do not wish this, I will inform my investigator/project manager.

If results from the data are handled for commercial purposes, I hereby make no claims on any part of this commercial use.

Place, Date	Signature of participant

Confirmation from the investigator: I hereby confirm that I have explained to this participant the nature, significance and implications of the further use of data.

Place, Date	Name and first name of the informing investigator in block capitals<br><br>Signature of the investigating physician

For peer review only - http://bmjopen.bmj.com/site/about/guidelines.xhtml

Reporting checklist for protocol of a clinical trial.

Based on the SPIRIT guidelines.

Instructions to authors

Complete this checklist by entering the page numbers from your manuscript where readers will find each of the items listed below.

Your article may not currently address all the items on the checklist. Please modify your text to include the missing information. If you are certain that an item does not apply, please write "n/a" and provide a short explanation.

Upload your completed checklist as an extra file when you submit to a journal.

In your methods section, say that you used the SPIRITreporting guidelines, and cite them as:

Chan A-W, Tetzlaff JM, Gøtzsche PC, Altman DG, Mann H, Berlin J, Dickersin K, Hróbjartsson A, Schulz KF, Parulekar WR, Krleža-Jerić K, Laupacis A, Moher D. SPIRIT 2013 Explanation and Elaboration: Guidance for protocols of clinical trials. BMJ. 2013;346:e7586

		Reporting Item	Page Number
**Administrative information**			
Title	#1	Descriptive title identifying the study design, population, interventions, and, if applicable, trial acronym	1
Trial registration	#2a	Trial identifier and registry name. If not yet registered, name of intended registry	4
Trial registration: data set	#2b	All items from the World Health Organization Trial Registration Data Set	n.a.
Protocol version	#3	Date and version identifier	4
Funding	#4	Sources and types of financial, material, and other support	4
Roles and responsibilities: contributorship	#5a	Names, affiliations, and roles of protocol contributors	4

For peer review only

Roles and responsibilities: sponsor contact information	#5b	Name and contact information for the trial sponsor	4
Roles and responsibilities: sponsor and funder	#5c	Role of study sponsor and funders, if any, in study design; collection, management, analysis, and interpretation of data; writing of the report; and the decision to submit the report for publication, including whether they will have ultimate authority over any of these activities	4
Roles and responsibilities: committees	#5d	Composition, roles, and responsibilities of the coordinating centre, steering committee, endpoint adjudication committee, data management team, and other individuals or groups overseeing the trial, if applicable (see Item 21a for data monitoring committee)	4
**Introduction**			
Background and rationale	#6a	Description of research question and justification for undertaking the trial, including summary of relevant studies (published and unpublished) examining benefits and harms for each intervention	7
Background and rationale: choice of comparators	#6b	Explanation for choice of comparators	n.a.
Objectives	#7	Specific objectives or hypotheses	9
Trial design	#8	Description of trial design including type of trial (eg, parallel group, crossover, factorial, single group), allocation ratio, and framework (eg, superiority, equivalence, non-inferiority, exploratory)	9
**Methods: Participants, interventions, and outcomes**			
Study setting	#9	Description of study settings (eg, community clinic,	9

For peer review only

		academic hospital) and list of countries where data will be collected. Reference to where list of study sites can be obtained	
Eligibility criteria	#10	Inclusion and exclusion criteria for participants. If applicable, eligibility criteria for study centres and individuals who will perform the interventions (eg, surgeons, psychotherapists)	10
Interventions: description	#11a	Interventions for each group with sufficient detail to allow replication, including how and when they will be administered	10
Interventions: modifications	#11b	Criteria for discontinuing or modifying allocated interventions for a given trial participant (eg, drug dose change in response to harms, participant request, or improving / worsening disease)	n.a.
Interventions: adherance	#11c	Strategies to improve adherence to intervention protocols, and any procedures for monitoring adherence (eg, drug tablet return; laboratory tests)	n.a.
Interventions: concomitant care	#11d	Relevant concomitant care and interventions that are permitted or prohibited during the trial	10
Outcomes	#12	Primary, secondary, and other outcomes, including the specific measurement variable (eg, systolic blood pressure), analysis metric (eg, change from baseline, final value, time to event), method of aggregation (eg, median, proportion), and time point for each outcome. Explanation of the clinical relevance of chosen efficacy and harm outcomes is strongly recommended	11
Participant timeline	#13	Time schedule of enrolment, interventions (including any run-ins and washouts), assessments, and visits for participants. A schematic diagram is highly recommended (see Figure)	13
Sample size	#14	Estimated number of participants needed to achieve study objectives and how it was determined, including clinical and statistical assumptions supporting any sample size	15

For peer review only

		calculations	
Recruitment	#15	Strategies for achieving adequate participant enrolment to reach target sample size	16
**Methods: Assignment of interventions (for controlled trials)**			
Allocation: sequence generation	#16a	Method of generating the allocation sequence (eg, computer-generated random numbers), and list of any factors for stratification. To reduce predictability of a random sequence, details of any planned restriction (eg, blocking) should be provided in a separate document that is unavailable to those who enrol participants or assign interventions	n.a.
Allocation concealment mechanism	#16b	Mechanism of implementing the allocation sequence (eg, central telephone; sequentially numbered, opaque, sealed envelopes), describing any steps to conceal the sequence until interventions are assigned	n.a.
Allocation: implementation	#16c	Who will generate the allocation sequence, who will enrol participants, and who will assign participants to interventions	n.a.
Blinding (masking)	#17a	Who will be blinded after assignment to interventions (eg, trial participants, care providers, outcome assessors, data analysts), and how	n.a.
Blinding (masking): emergency unblinding	#17b	If blinded, circumstances under which unblinding is permissible, and procedure for revealing a participant's allocated intervention during the trial	n.a.
**Methods: Data collection, management, and analysis**			
Data collection plan	#18a	Plans for assessment and collection of outcome, baseline, and other trial data, including any related processes to promote data quality (eg, duplicate	16

For peer review only

		measurements, training of assessors) and a description of study instruments (eg, questionnaires, laboratory tests) along with their reliability and validity, if known. Reference to where data collection forms can be found, if not in the protocol	
Data collection plan: retention	#18b	Plans to promote participant retention and complete follow-up, including list of any outcome data to be collected for participants who discontinue or deviate from intervention protocols	16
Data management	#19	Plans for data entry, coding, security, and storage, including any related processes to promote data quality (eg, double data entry; range checks for data values). Reference to where details of data management procedures can be found, if not in the protocol	18
Statistics: outcomes	#20a	Statistical methods for analysing primary and secondary outcomes. Reference to where other details of the statistical analysis plan can be found, if not in the protocol	19
Statistics: additional analyses	#20b	Methods for any additional analyses (eg, subgroup and adjusted analyses)	20
Statistics: analysis population and missing data	#20c	Definition of analysis population relating to protocol non-adherence (eg, as randomised analysis), and any statistical methods to handle missing data (eg, multiple imputation)	20
**Methods: Monitoring**			
Data monitoring: formal committee	#21a	Composition of data monitoring committee (DMC); summary of its role and reporting structure; statement of whether it is independent from the sponsor and competing interests; and reference to where further details about its charter can be found, if not in the protocol. Alternatively, an explanation of why a DMC is not needed	4<br>project scientific board (PSB)

For peer review only

Data monitoring: interim analysis	#21b	Description of any interim analyses and stopping guidelines, including who will have access to these interim results and make the final decision to terminate the trial	n.a.
Harms	#22	Plans for collecting, assessing, reporting, and managing solicited and spontaneously reported adverse events and other unintended effects of trial interventions or trial conduct	14
Auditing	#23	Frequency and procedures for auditing trial conduct, if any, and whether the process will be independent from investigators and the sponsor	18
**Ethics and dissemination**			
Research ethics approval	#24	Plans for seeking research ethics committee / institutional review board (REC / IRB) approval	21
Protocol amendments	#25	Plans for communicating important protocol modifications (eg, changes to eligibility criteria, outcomes, analyses) to relevant parties (eg, investigators, REC / IRBs, trial participants, trial registries, journals, regulators)	21
Consent or assent	#26a	Who will obtain informed consent or assent from potential trial participants or authorised surrogates, and how (see Item 32)	21
Consent or assent: ancillary studies	#26b	Additional consent provisions for collection and use of participant data and biological specimens in ancillary studies, if applicable	n.a.
Confidentiality	#27	How personal information about potential and enrolled participants will be collected, shared, and maintained in order to protect confidentiality before, during, and after the trial	22
Declaration of interests	#28	Financial and other competing interests for principal investigators for the overall trial and each study site	22
Data access	#29	Statement of who will have access to the final trial dataset, and disclosure of contractual agreements	22

For peer review only

		that limit such access for investigators	
Ancillary and post trial care	#30	Provisions, if any, for ancillary and post-trial care, and for compensation to those who suffer harm from trial participation	n.a.
Dissemination policy: trial results	#31a	Plans for investigators and sponsor to communicate trial results to participants, healthcare professionals, the public, and other relevant groups (eg, via publication, reporting in results databases, or other data sharing arrangements), including any publication restrictions	23
Dissemination policy: authorship	#31b	Authorship eligibility guidelines and any intended use of professional writers	23
Dissemination policy: reproducible research	#31c	Plans, if any, for granting public access to the full protocol, participant-level dataset, and statistical code	22
**Appendices**			
Informed consent materials	#32	Model consent form and other related documentation given to participants and authorised surrogates	Supplementary file
Biological specimens	#33	Plans for collection, laboratory evaluation, and storage of biological specimens for genetic or molecular analysis in the current trial and for future use in ancillary studies, if applicable	n.a.

None The SPIRIT checklist is distributed under the terms of the Creative Commons Attribution License CC-BY-ND 3.0. This checklist can be completed online using https://www.goodreports.org/, a tool made by the EQUATOR Network in collaboration with Penelope.ai

For peer review only

BMJ Open

Protocol for the ARCR_Pred cohort-study: Swiss-wide multicenter evaluation and prediction of core outcomes in arthroscopic rotator cuff repair

Journal:	*BMJ Open*
Manuscript ID	bmjopen-2020-045702.R1
Article Type:	Protocol
Date Submitted by the Author:	20-Mar-2021

Complete List of Authors:	Audigé, Laurent ; Schulthess Klinik, Research and Development; University Hospital Basel, Orthopaedic Surgery and Traumatology<br>Bucher, Heiner; University Hospital Basel, Basel Institute for Clinical Epidemiology and Biostatistics<br>Aghlmandi, Soheila; University Hospital Basel, Basel Institute for Clinical Epidemiology and Biostatistics<br>Stojanov, Thomas; University Hospital Basel, Orthopaedic Surgery and Traumatology; University Hospital Basel, Basel Institute for Clinical Epidemiology and Biostatistics<br>Schwappach, David; Patient Safety Foundation; University of Bern, Institute of Social and Preventive Medicine<br>Hunziker, Sabina; University Hospital Basel, Medical Communication / Psychosomatic Medicine<br>Candrian, Christian; Ospedale Regionale di Lugano, Trauma and Ortho Unit<br>Cunningham, Gregory; Hirslanden Clinique La Colline, Shoulder Center; Geneva University Hospitals, Division of Orthopaedics and Trauma Surgery, Department of Surgery<br>Durchholz, Holger; Klinik Gut Sankt Moritz<br>Eid, Karim; Baden Cantonal Hospital, Clinic for Orthopaedics and Traumatology<br>Flury, Matthias; In-Motion, Center for Orthopaedics and Neurosurgery<br>Jost, B; Cantonal Hospital of St.Gallen, Clinic for Orthopaedic Surgery and Traumatology of the Musculoskeletal system<br>Ladermann, Alexandre; La Tour Hospital, Division of Orthopaedics and Trauma Surgery; Geneva University Hospitals, Division of Orthopaedics and Trauma Surgery, Department of Surgery<br>Moor, Beat; Hôpital de Martigny Centre Hospitalier du Valais Romand, Service for Orthopaedics and Traumatology of the Musculoskeletal system<br>Moroder, Philipp; Charite Universitatsmedizin Berlin, Department of Shoulder and Elbow Surgery, Center for Musculoskeletal Surgery,<br>Rosso, Claudio; Arthro Medics, Shoulder and Elbow Center<br>Schär, Michael; Inselspital Universitatsspital Bern, Department of Orthopaedic Surgery and Traumatology<br>Scheibel, Markus; Schulthess Klinik, Shoulder and Elbow Surgery,; Charité Universitätsmedizin Berlin, Department for Shoulder and Elbow surgery - Center for Musculoskeletal Surgery- Campus Virchow<br>Spormann, Christophe; Endoclinic, Center for Endoprosthetics and Joint

	Surgery<br>Suter, Thomas; Canton Hospital Baselland, Orthopaedic Shoulder and Elbow<br>Wieser , Karl ; University of Zurich, Department of Orthopaedics, Balgrist University Hospital<br>Zumstein, Matthias; Orthopaedics Sonnenhof, Shoulder, Elbow and Orthopaedic Sports Medicine<br>ARCR_Pred Study Group, Name; University Hospital Basel<br>Müller, Andreas; University Hospital Basel, Orthopaedic Surgery and Traumatology
**Primary Subject Heading**:	Surgery
Secondary Subject Heading:	Epidemiology, Evidence based practice
Keywords:	ORTHOPAEDIC & TRAUMA SURGERY, Musculoskeletal disorders < ORTHOPAEDIC & TRAUMA SURGERY, Shoulder < ORTHOPAEDIC & TRAUMA SURGERY

SCHOLARONE™
Manuscripts

I, the Submitting Author has the right to grant and does grant on behalf of all authors of the Work (as defined in the below author licence), an exclusive licence and/or a non-exclusive licence for contributions from authors who are: i) UK Crown employees; ii) where BMJ has agreed a CC-BY licence shall apply, and/or iii) in accordance with the terms applicable for US Federal Government officers or employees acting as part of their official duties; on a worldwide, perpetual, irrevocable, royalty-free basis to BMJ Publishing Group Ltd ("BMJ") its licensees and where the relevant Journal is co-owned by BMJ to the co-owners of the Journal, to publish the Work in this journal and any other BMJ products and to exploit all rights, as set out in our licence.

The Submitting Author accepts and understands that any supply made under these terms is made by BMJ to the Submitting Author unless you are acting as an employee on behalf of your employer or a postgraduate student of an affiliated institution which is paying any applicable article publishing charge ("APC") for Open Access articles. Where the Submitting Author wishes to make the Work available on an Open Access basis (and intends to pay the relevant APC), the terms of reuse of such Open Access shall be governed by a Creative Commons licence – details of these licences and which Creative Commons licence will apply to this Work are set out in our licence referred to above.

Other than as permitted in any relevant BMJ Author's Self Archiving Policies, I confirm this Work has not been accepted for publication elsewhere, is not being considered for publication elsewhere and does not duplicate material already published. I confirm all authors consent to publication of this Work and authorise the granting of this licence.

For peer review only

Protocol for the ARCR_Pred cohort-study: Swiss-wide multicenter evaluation and prediction of core outcomes in arthroscopic rotator cuff repair

Laurent Audigé[1,2,3†], Heiner C. Bucher[3], Soheila Aghlmandi[3], Thomas Stojanov[1,2,3], David Schwappach[4,5], Sabina Hunziker[6], Christian Candrian[7], Gregory Cunningham[8,9], Holger Durchholz[10], Karim Eid[11], Matthias Flury[12], Bernhard Jost[13], Alexandre Lädermann[9,14], Beat Moor[15], Philipp Moroder[16], Claudio Rosso[17], Michael Schär[18], Markus Scheibel[16,19], Christophe Spormann[20], Thomas Suter[21], Karl Wieser[22], Matthias A. Zumstein[23,24], ARCR_Pred Study Group*, Andreas Müller[1]

Author affiliations:

1. Orthopaedic Surgery and Traumatology, University Hospital of Basel, Basel, Switzerland
2. Research and Development Department, Schulthess Klinik, Zurich, Switzerland
3. Basel Institute for Clinical Epidemiology and Biostatistics, University Hospital of Basel, Basel, Switzerland
4. Swiss Patient Safety Foundation, Zurich, Switzerland
5. Institute of Social and Preventive Medicine (ISPM), University of Bern, Bern, Switzerland
6. Medical Communication / Psychosomatic, University Hospital of Basel, Basel, Switzerland
7. Trauma and Ortho Unit, Ospedale Regionale di Lugano, Lugano, Switzerland
8. Shoulder Center, Hirslanden Clinique la Colline, Geneva, Switzerland
9. Division of Orthopaedics and Trauma Surgery, Department of Surgery, Geneva University Hospitals, Geneva, Switzerland
10. Klinik Gut, St Moritz, Switzerland
11. Clinic for Orthopaedics and Traumatology, Cantonal Hospital of Baden, Baden, Switzerland
12. Center for Orthopaedics and Neurosurgery, In-Motion, Wallisellen, Switzerland
13. Clinic for Orthopaedic Surgery and Traumatology of the Musculoskeletal system , Cantonal Hospital of St.Gallen, St.Gallen, Switzerland
14. Division of Orthopaedics and Trauma Surgery, La Tour Hospital, Meyrin, Switzerland
15. Service for Orthopaedics and Traumatology of the Musculoskeletal system , Hôpital du Valais – Centre Hospitalier du Valais Romand, Martigny, Switzerland

For peer review only - http://bmjopen.bmj.com/site/about/guidelines.xhtml

16. Department for Shoulder and Elbow surgery - Center for Musculoskeletal Surgery- Campus Virchow, Charitè Medicine University, Berlin, DE

17. Shoulder and Elbow Center, ARTHRO Medics, Basel, Switzerland

18. Department of Orthopaedic Surgery and Traumatology, Inselspital, Bern University Hospital, University of Bern, Switzerland

19. Shoulder and Elbow Surgery, Schulthess Klinik, Zurich, Switzerland

20. Center for Endoprosthetics and Joint Surgery, Endoclinic, Zürich, Switzerland

21. Orthopaedic Shoulder and Elbow, Canton Hospital Baselland, Bruderholz, Switzerland

22. Department of Orthopaedics, Balgrist University Hospital, University of Zurich, Switzerland

23. Shoulder, Elbow and Orthopaedic Sports Medicine, Orthopaedics Sonnenhof, Bern, Switzerland

24. Stiftung Lindenhof, Campus SLB, Swiss Institute for Translational and Entrepreneurial Medicine, Bern, Switzerland

†Corresponding author:

Laurent Audigé, DVM PhD

Schulthess Klinik

Research and Development - Shoulder and Elbow Surgery

Lengghalde 2, CH-8008 Zurich, Switzerland

Phone: +41 44 385 7580; Fax: +41 44 385 7590

E-mail: laurent.audige@kws.ch

* Other members of the ARCR_Pred Study Group are listed below per site:

ARTHRO Medics, Basel, Switzerland (ART): Jannine Buchschacher, Lena Fankhauser, Corinne Eicher, Gernot Willscheid; **Charitè Medicine University, Berlin, Germany (BER)**: Doruk Akgün, Kathi Thiele, Marvin Minkus, Victor Danzinger, Katrin Karpinski; **Canton Hospital Baselland, Bruderholz, Switzerland (BRU)**: Sebastian Mueller, Claudia Haag-Schumacher; **Centre Hospitalier du Valais Romand, Martigny, Switzerland (CHV)**: Viviane Steffen, Sarah Fournier, Deborah Marietan, Sebastien Pawlak; **Endoclinic, Zürich,**

For peer review only

Switzerland (END): Britta Hansen, Ferdinand Lovrek, Marco Zanetti, Nadja Mamisch; **Klinik Gut, St Moritz, Switzerland (GUT)**: Christian Steiner, Georg Ahlbäumer, Jakob Bräm, Jens Fischer, Alexander Delvendahl; **Hirslanden Clinique la Colline, Geneva, Switzerland (HIR)**: Patricia Simao, Abed Khourani; **La Tour Hospital, Meyrin, Switzerland (HUG)**: Anne-Sophie Foucault, Frank Kolo; **Inselspital, Bern University Hospital, Bern, Switzerland (INB)**: Adrian Schenk, Johannes Weihs, Remy Flückiger, Philipp Scacchi, Paolo Lombardo; **In-Motion, Wallisellen, Switzerland (INM)**: Larissa Hübscher, Ralph Berther, Christine Ehrmann; **Cantonal Hospital of Baden, Baden, Switzerland (KSB)**: Raffaela Nobs, Richard Niehaus, Nisha Grünberger, Philipp Kriechling, Susanne Bensler; **Schulthess Klinik, Zurich, Switzerland (KWS)**: Hans-Kaspar Schwyzer, Fabrizio Moro, Michael Glanzmann, Barbara Wirth, Christian Jung, Florian Freislederer, Manuela Nötzli, Frederik Bellmann, Franz Anne, Jörg Oswald, Alex Marzel, Cécile Grobet, Marije de Jong, Martina Wehrli, Jan Schätz; **Ospedale Regionale di Lugano, Lugano, Switzerland (LUG)**: Francesco Marbach, Marco Delcogliano, Davide Previtali, Florian Schönweger, Elena Porro, Gabriela Induni-Lang, Giuseppe Filardo, Filippo Del Grande, Pietro Feltri, Schiavon Guglielmo; **Cantonal Hospital of St.Gallen, St.Gallen, Switzerland (SGA)**: Christian Spross, Martin Olach, Michael Badulescu, Vilijam Zdravkovic, Stephanie Lüscher, Jörg Scheler, Lena Öhrström; **Orthopädie Sonnenhof, Berne, Switzerland (SON)**: Annabel Hayoz, Frederick Schuster, Julia Müller-Lebschi; **Balgrist University Hospital, Zurich, Switzerland (UKB)**: Christian Gerber, Samy Bouaicha, Paul Borbas, Anita Hasler, Florian Grubhofer, Sabrina Catanzaro, Sabine Wyss, Reto Sutter; **University Hospital of Basel, Basel, Switzerland (USB)**: Mohy Taha, Cornelia Baum, Ilona Ahlborn, Simone Hatz, Giorgio Tamborrini-Schütz; **University Library Basel, Basel, Switzerland**: Christian Appenzeller-Herzog.

For peer review only

Administrative information

Title Protocol for the ARCR_Pred cohort-study: Swiss-wide multicenter evaluation and prediction of core outcomes in arthroscopic rotator cuff repair

Trial registration ClinicalTrial.gov registration number NCT04321005

Protocol version Version 2 (13.12.2019)

Funding This project is funded by the Swiss National Science Foundation (SNF Project ID 320030_184959, http://p3.snf.ch/project-184959). A complementary grant was provided by Swiss Orthopedics. The sites Charitè Medicine University, Berlin, Germany (BER) and University Clinic Balgrist, Zurich, Switzerland (UKB) are funding their own participation in the project.

For peer review only

Roles and responsibilities

LA and AM are the initiators and project leaders. LA, AM, HB, SA, DS and SH were involved in the study design, which was reviewed and commented by principal investigators CC, GC, HD, KE, MF, BJ, AL, BM, PM, CR, MS, MS, CS, T Suter, KW and MZ.

Preparation of the manuscript was done by LA, AM, SA and T Stojanov. HB, DS and SH edited and critically revised the paper. All authors have read and approved the manuscript.

LA is the guarantor of the manuscript.

This is an investigator initiated project at the University Hospital of Basel. The principal investigator and project leader (AM) is the official sponsor representative for the project and was involved in all phases of the project from its conception to the current implementation steps. The project initiators and project leaders (LA and AM) have ultimate authority over any of the project activities.

A project scientific board (PSB) comprises the project leaders (LA and AM), project investigators at each site (CC, GC, HD, KE, MF, BJ, AL, BM, PM, CR, MS, MS, CS, T Suter,

For peer review only - http://bmjopen.bmj.com/site/about/guidelines.xhtml

KW and MZ), and project partners (SA, HB, DS and SH). The PSB shall meet at specific time points during the study: before the study start, after completion of recruitment and the 12-month follow-up, and at the end of the study. The agenda of these meetings will focus on (however is not limited to) patient enrollment and the completion of follow-up examinations and questionnaires, the documentation process in REDCap, data quality issues (completeness and consistency), monitoring activities, adverse event assessment and management, baseline patient description, ranking of prognostic factors for prognostic models, progress of data analysis, publication strategy and decisions regarding data sharing. Between these meetings, communication will be maintained between the project coordinating team and investigators via various channels including emails, quarterly newsletters, phone calls and (video) conference calls as required.

Competing interests

There are no competing interests for any author

For peer review only

Abstract

Introduction In the field of arthroscopic rotator cuff repair (ARCR), reporting standards of published studies differ dramatically, notably concerning adverse events (AEs). In addition, prognostic studies are overall methodologically poor, based on small datasets, and explore only limited numbers of influencing factors. We aim to develop prognostic models for individual ARCR patients, primarily for the patient-reported assessment of shoulder function (Oxford Shoulder Score [OSS]) and the occurrence of shoulder stiffness 6 months after surgery. We also aim to evaluate the use of a consensus Core Event Set (CES) for AEs and validate a severity classification for these events, considering the patient's perspective.

Methods and analysis A cohort of 970 primary ARCR patients will be prospectively documented from several Swiss and German orthopedic clinics up to 24 months postoperatively. Patient clinical examinations at 6 and 12 months will include shoulder range of motion and strength (Constant Score). Tendon repair integrity status will be assessed by ultrasound at 12 months. Patient-reported questionnaires at 6, 12 and 24 months will determine functional scores (Subjective Shoulder Value, OSS), anxiety and depression scores, working status, sports activities, quality of life (EuroQol EQ-5D-5L). AEs will be documented according to a CES. Prognostic models will be developed using an internationally supported regression methodology. Multiple prognostic factors, including patient baseline demographics, psychological, socioeconomic and clinical factors, rotator cuff integrity, concomitant local findings, and (post)operative management factors will be investigated.

Ethics and dissemination This project contributes to the development of personalized risk predictions for supporting the surgical decision process in ARCR. The consensus CES may become an international reference for the reporting of complications in clinical studies and registries. Ethical approval was obtained on April 1st, 2020, from the lead ethics committee

For peer review only

(EKNZ, Basel Switzerland; ID: 2019-02076). All participants will provide informed, written consent before enrollment in the study.

Strengths and limitations of this study

- Large prospective multicenter observation of routine care
- Assessment of patient-reported outcomes (PROMs)
- Implementation of an international core outcome set of adverse events (CES)
- Internationally supported methodology for prognostic model development
- Potential limited response to patient questionnaires at 24 months

Introduction

Rotator cuff tears are one of the most common injuries of the shoulder joint, which may cause pain and disability associated with severe restrictions in daily activities. Surgical repair is indicated when nonoperative treatment fails or follows extended traumatic tears, notably inactive patients without signs of advanced tendon degeneration or muscle fat infiltration [1]. Clinical studies have demonstrated clinically-relevant improvement in shoulder function and quality of life after arthroscopic rotator cuff tear repair (ARCR) [2-5]. The number of ARCRs has increased over the last two decades [6 7 8 9] due to several contributing factors such as an aging yet active population, improvements in operative repair techniques, and more liberal indications for ARCRs.

Not all patients, however, benefit from ARCR [10]. Patients may be affected by complications and/or adverse events (AEs) like persistent pain, shoulder stiffness, infection, neurological problems, and repair failures [11 12]. About 20% of patients may show, typically between 6 and 12 months following ARCR, a persistent rotator cuff defect [13]. Patients with healed tendons may show better functional outcome after repair [2 14 15]. Postoperative shoulder stiffness, a major complication reported to occur in 1.5% to 11.1% of ARCRs [11], leads to limitations in everyday activities, prolonged rehabilitation, and, in severe cases, to reoperation (capsular

For peer review only - http://bmjopen.bmj.com/site/about/guidelines.xhtml

release) [16-18]. Nonetheless, incident data on outcome and AEs are impaired by the heterogeneity in definition and reporting [13 19].

Valid and representative data on the safety and effectiveness of ARCR are nonexistent at the Swiss national level. However, such data is paramount for optimizing the indication and outcome of ARCR, and for benchmarking orthopedic clinics. Reporting standards are a prerequisite for outcome and safety data. Recently, a Core Outcome Set (COS) [20] was defined for shoulder disorders, which includes inner core domains of pain, physical function and activities, global perceived effect (a person's assessment of their recovery or degree of improvement), and AEs [21 22]. A Core Event Set (CES) was developed by international consensus in ARCR [23 24] and lay the ground for the current project.

Appropriate indication of ARCR and judgment on risks for AEs or unsatisfactory patient outcomes rely on validated clinical prediction tools [25 26], which are still sparse in the field of surgical repair of a rotator cuff tear. Currently existing models focus on early surgical repair [27], tendon healing [28 29] or shoulder functional outcomes [30]. A model for shoulder stiffness included patients with various shoulder pathologies and surgeries [31]. Furthermore, individual outcome predictions in ARCR require the identification of relevant patient and management factors. Several systematic reviews have highlighted the general lack of qualitative studies focused on prognostic factors for ARCR outcomes [32-36]. In addition, we have observed the substantial heterogeneity in terms of applied methodology, core outcomes and studied prognostic factors, where certain factors (e.g., age, tear size, muscle degeneration, smoking) are given greater focus over others (e.g., sex, traumatic onset). The reviews highlight the need for more robust prospective studies to include additional patient-reported outcomes in a multivariable context.

For peer review only

For peer review only - http://bmjopen.bmj.com/site/about/guidelines.xhtml

Objectives

The overall objective is to establish a prospective cohort of patients undergoing ARCR with standardized data collection and follow-up for the evaluation and prediction of targeted core safety, and clinical and patient-reported outcome parameters that are to be routinely collected in standard clinical care.

The primary objective is to develop predictive models for two core outcome parameters:, 1) the patient-reported Oxford Shoulder Score (OSS) functional outcome, and 2) the occurrence of shoulder stiffness (primary safety event) as reported by patients and clinicians.

Secondary objectives are 1) to evaluate the content and applicability of the defined consensus CES (i.e. ARCR CES 1.0) [23] in routine practice considering the patient's perspective, 2) to quantify the incidence of AE up to 24 months after surgery (e.g., persisting or worsening pain, recurrent rotator cuff (RC) defect), 3) to validate an adapted severity classification for postoperative local AEs [12 37], and 4) to develop predictive models for other clinically-relevant outcome parameters including patient-reported outcomes (e.g., perception of improvement, return to work, return to sports, quality of life, satisfaction with surgery, acceptability of symptom state), clinical outcomes (e.g., shoulder strength and motion) and specific AEs (e.g. RC defect at 12 months).

Methods and analysis

Study design and setting

This is a prospective multicenter cohort study in patients undergoing ARCR with 17 participating orthopedic centers in Switzerland and one German center.

Several sub-projects, associated with the main ARCR cohort study, are planned and include a systematic review of prognostic studies in ARCR, the application of the ARCR CES 1.0 for AE documentation, and the application and validation of an AE severity classification

For peer review only - http://bmjopen.bmj.com/site/about/guidelines.xhtml

Eligibility criteria

Adult patients diagnosed with a partial or full-thickness RC tear by magnetic resonance imaging (MRI), planned for a primary arthroscopic surgical repair, and giving their informed consent to participate in the cohort study will be included. Patients undergoing a specific surgical procedure for irreparable tears (i.e. tendon transfer, subacromial spacer or superior capsular reconstruction), revision operations, open or mini-open reconstructions will be excluded. Patients unable to give written informed consent or attend clinics for follow-up visits, not fluent in German, French, Italian, or English or pregnant females will be excluded. Patients undergoing bilateral ARCR will only be included for their first intervention.

Intervention

Shoulder arthroscopies will be performed according to standardized clinic-specific and international guidelines [38] in the context of routine care with patients in a beach-chair or lateral decubitus position under general or local anesthesia. The variability in the repair techniques used between clinics and surgeons will be documented. Typically, after the diagnostic arthroscopy to assess the type of RC tear (partial or full-thickness tear and involved tendons, tendon tear delamination, sign of tendon degeneration) and concomitant injuries or lesions, the ruptured tendons are mobilized until they can be repositioned on the original footprint with as little tension as possible. Tendon fixation may be performed using one of multiple anchor and suture configurations according to the surgeon's decision. An intervention at the biceps tendon is performed if any tendinopathy, or lesions to the superior labrum or biceps pulley system are observed. An anterolateral or lateral acromioplasty is performed at the surgeon’s discretion, generally in the presence of a hooked-shaped acromion or a critical shoulder angle larger than 35°, respectively. Operative details, including additional concomitant procedures (acromioplasty, acromioclavicular joint resection, capsulotomy, and biceps tenotomy or tenodesis) and operation duration are recorded immediately after surgery. A standard 3-phase postoperative rehabilitation scheme

For peer review only - http://bmjopen.bmj.com/site/about/guidelines.xhtml

is usually prescribed and will be documented in detail, including immobilization and passive mobilization in the first phase, active mobilization and coordination training in the second phase, followed by the third phase of specific progressive resistance exercises.

Outcomes

The *first primary outcome* is the patient-reported change in shoulder functional outcome between baseline and 6 months postoperatively as measured with the Oxford Shoulder Score (OSS). [39] The OSS is a condition-specific questionnaire developed for patients with a degenerative or inflammatory state of the shoulder. It contains twelve items to be answered by the patient independently, which deal with pain (degree, time point) and possible handicaps in private and professional life. There are five categories of response for every question, corresponding to a score ranging from 0 to 4. Scores are summed to give a single score with a range from 0 (worst outcome) to 48 (best outcome). Transcultural validations of this questionnaire for the German and Italian populations have been performed [40 41] and are validated for patient-based outcomes after RC repair [42-44]. While functional outcome at the last 24-month follow-up is clinically relevant, the early 6-month primary time point is chosen because of the importance in early surgical recovery and rehabilitation, particularly when considering the socioeconomic impact on professionally active patients [4].

The *second primary outcome* is the occurrence of shoulder stiffness within 6 months after surgery: this event is poorly defined in the literature [19]. We formed a consensus definition of shoulder stiffness among specialized shoulder surgeons in a Delphi survey, which describes a postoperative restriction in passive shoulder motion diagnosed within 6 months after ARCR in at least two of the motion planes of flexion, abduction and external rotation in 0° abduction. Motion restriction is to be assessed separately for each plane according to specific threshold criteria (flexion: total motion equal to or below 90° or glenohumeral motion equal to or below 80°; abduction: total motion equal to or below 80° or glenohumeral motion equal to or below 60°; external rotation in 0° abduction: glenohumeral motion equal to or below 20° or no more

For peer review only - http://bmjopen.bmj.com/site/about/guidelines.xhtml

than 50% of the contralateral side value). In this project, we will identify cases of shoulder stiffness based on our consensus definition as well as clinical records and reports from clinicians and their patients.

Secondary outcomes will include: 1) local AEs according to the ARCR CES, in particular the occurrence of recurrent defect of repaired tendon(s) at 12 months, when at least one repaired tendon is diagnosed with a recurrent defect by ultrasound examination, persistent or worsening pain, infection, any local event (composite outcome); 2) functional parameters of the Constant score (CS) [45] at 6 and 12 months, shoulder strength (kg) in abduction at 6 and 12 months, patient-reported shoulder pain on the numeric rating scale (NRS) at 6, 12 and 24 months, patient-reported shoulder function: OSS at 6, 12 and 24 months, Subjective Shoulder Value (SSV) [46] assessment at 6, 12 and 24 months; 3) general health and socioeconomic parameters including patient-reported quality of sleep (NRS) at 6, 12 and 24 months [47], return to work, change of working condition within 6, 12 and 24 months, level of depression and anxiety at 6, 12 and 24 months based on Patient-Reported Outcomes Measurement Information System (PROMIS) scores [48 49], patient perceived shoulder improvement, acceptability of own symptom state[50], quality of life (utilities and general health) at 6, 12 and 24 months using the European Quality of Life 5 Dimensions 5 Level questionnaire (EQ-5D-5L), patient satisfaction with the surgical outcome at 12 and 24 months; 4) safety outcome assessment, occurrence of all AEs reported by clinicians and patients (including non-local AEs within 6 months after surgery), final independent surgeon and patient-rated assessment of AEs according to perceived severity (rating scale from 0 [no complication] to 100 [death] [51]), comprehensive Complication Index[51] considering all AEs that occurred within 6 months after surgery.

Shoulder ultrasound examinations will be performed at 12 months by experienced clinicians independent of the operating surgeons. The repair integrity will be graded according to the Sugaya classification (where grade 4 or 5 defines the occurrence of a recurrent effect) [52 53].

For peer review only - http://bmjopen.bmj.com/site/about/guidelines.xhtml

Other ultrasound parameters include the location of the recurrent defect (at the footprint | medial cuff failure), long biceps tendon status, signs of anchor displacement, and location and signs of suture cut-through.

Participant timeline

Local investigators will identify patients who meet the eligibility criteria. Patient enrollment started on June 1st, 2020 and is planned for a maximum period of 15 months. Patients will complete a preoperative evaluation no more than 2 months before surgery. Follow-up assessments will be performed at 6 weeks (+/- 1 week), and 6 (+/- 1 month), 12 (+/- 1 month) and 24 months (+/- 2 months) postoperatively. At the final 24-month time point, only patient self-reporting assessments, including surveys on adverse events, will be documented (Figure 1). The end date for the study representing the collection of the last patient questionnaire is expected on November 1st, 2023.

Baseline prognostic factors

Various baseline parameters, operative details, and postoperative management variables are known or suspected to influence ARCR outcomes [32-36].

The following patient-related factors will be recorded: patient demographics [year of birth for age, sex], socioeconomic parameters [nationality, marital status, the highest level of education, employment status, last occupational position, daily physical workload], dominant side, smoking & drinking status, general physical and mental health [Body Mass Index (BMI) and obesity, American Society of Anesthesiologists (ASA) classification, comorbidities (e.g. diabetes), concomitant medication, level of depression and anxiety (PROMIS Depression and Anxiety Short Form 4a) [48 49], quality of life (EQ-5D-5L) [54]].

Disease-related factors are shoulder clinical examinations [pain level on a numeric rating scale (NRS), range of motion, muscle strength, Constant Score [45]], patient-reported shoulder

For peer review only - http://bmjopen.bmj.com/site/about/guidelines.xhtml

function (see outcome measures), radiograph parameters [Critical Shoulder Angle [55], acromiohumeral distance [56]], MRI or arthro-Computer Tomography (CT) parameters [supraspinatus muscle atrophy [57], tangent sign [58], grade of fatty infiltration [59 60]], medical history [cause of injury (trauma event), symptom duration, previous interventions (operation and timing of surgery), actual medication, and the extent of physical therapy].

Rotator cuff integrity and concomitant local findings. The RC tear will be determined by MRI (or arthro-CT) and confirmed intraoperatively: tear size: location (involved tendons) and grading (partial / complete), tendon retraction grade [61] and tear sagittal size [62] , status of the biceps tendon, additional intraoperative observation of concomitant local injuries [Superior Labrum from Anterior to Posterior (SLAP) lesion, Humeral Avulsion Glenohumeral Ligament (HAGL), Bankart lesion, humeral and glenoid-side chondral lesions].

Operative details and postoperative management: type of ARCR procedure [use of anchors, suture techniques], augmentation techniques [e.g. platelet concentrates, scaffolds, …] [63], additional concomitant treatment [acromioplasty, acromioclavicular joint resection, capsulotomy, biceps tenotomy or tenodesis, treatment of SLAP lesion], operation duration, duration of hospital stay, postoperative management [immobilization position and duration, pain medication [e.g. using non-steroidal anti-inflammatory drugs], timing of passive and active shoulder motion, physiotherapy and muscle training].

Adverse event documentation and assessment process

Operating surgeons will report the occurrence of any intraoperative AE on the operation form. The occurrence of postoperative local AEs within 24 months will be reported by investigators at the clinical examination and by patients on the questionnaires. The occurrence of postoperative non-local AEs that are unrelated to the operation will be documented in a similar manner, however only within 6 months after surgery. An AE form was developed according to the ARCR CES 1.0 [23]. Each AE documentation will be structured

For peer review only - http://bmjopen.bmj.com/site/about/guidelines.xhtml

after Audigé et al. [64] and includes the date/period of occurrence [intra- / postoperative], the affected body location [local at the operated shoulder / non-local], the event group and specification, applied health-related intervention(s) [operative / nonoperative procedure(s)], its outcome at the time of reporting (or end of the study), and the assessment of the event [causal factor(s) / severity grade / seriousness]. Severity grading will be made according to existing and adapted systems for intraoperative [65 66] and postoperative [12 37] complications. The documentation of AEs will be checked for completeness and consistency remotely as well as on-site by reviewing selected patient files as part of the monitoring plan. A review committee (LA, AM, TS, HD and DS) will assess all events reported by surgeons and patients, and generate queries to the respective sites as required.

Fully documented local events, including their treatment, outcome and possible causative factors, will be formulated in layman's terms and sent back to the affected patients, so that they can confirm and validate collected AE data as well as assess their severity on a visual analogue scale from 0 (not at all severe) to 100 (extremely severe). This subsequent rating will also be performed by their treating surgeon and four other randomly-selected surgeons involved in the project, blinded from the original severity grading.

Sample size determination

For sample size calculation, we set up a simulation study and used multiple regression to predict the change in OSS within 6 months for the most important prognostic factors. The prognostic factors were derived from an existing ARCR local registry [67] and include age, sex, body mass index/obesity status, tendon quality/degeneration, and RC severity [32-36]. We accounted for the type I error at 5% for statistical significance and the type II error set at 20% for 80% statistical power [68]. Two thousand replications were done, and the p-values were recorded to calculate the mean significance for each of the prognostic factors to reach a minimum of 80% statistical power. This approach led to a sample of 920 patients.

For peer review only - http://bmjopen.bmj.com/site/about/guidelines.xhtml

For the second primary outcome of shoulder stiffness, we accounted for a minimum of ten events per variable to allow for the inclusion of a maximum of ten predictors into the model [69 70]. The estimated event rate for shoulder stiffness from our pilot data set was 8.3%, which, according to our experience, might reflect an underestimation of the true rate [17]. Therefore, a 10% stiffness rate was assumed, which resulted in a sample size calculation of 900 patients. The higher resulting number determines the final number of patients to be recruited. Therefore, 920 patients will be included with an additional 50 patients (i.e. 970 patients) due to the anticipated maximum dropout rate of 5% at 6 months (based on personal experience).

Recruitment

Study sites and local investigators were selected based on their expertise in ARCR with support by the shoulder and elbow expert group of Swiss Orthopaedics. Each site was visited by the project leaders to assess the adequacy of local clinical and research settings for the project as well as to ensure prior interest and commitment. The number of included sites was determined based on the reported estimate of the number of ARCR patients that could be realistically enrolled within one year from each site, and included an allowance for overestimation (i.e. all sites together estimated that they could recruit up to 40% more than the expected 970 patients within one year).

Patients who are enrolled after signing an informed consent form are definitively recruited for the project after documentation of baseline parameters (clinical examinations and patient questionnaires) and confirmation of ARCR during surgery. A recruitment curve is prepared every 2 weeks and sent to the project sites along with a recruitment table presenting the performance of each site. Sites that are unable to recruit the expected number of patients within the first 3 months will be considered for exclusion from the project and replaced by additional sites if the estimated total duration of patient enrollment is delayed for more than 3 months.

Data collection methods

For peer review only - http://bmjopen.bmj.com/site/about/guidelines.xhtml

Data are collected on electronic or paper-based case report forms or patient questionnaires. Project parameters and used instruments are presented in previous sections of this protocol. A training video was prepared for the collection of CS data [45]. For the measurement of shoulder muscle strength, several devices were permitted, i.e. IsoForceControl® (MDS Medical Device Solution, Oberburg, Switzerland), Mark-10 Force Gauge (Mark-10 Corporation, Copiague, USA) as well as hand-held (Lafayette Instrument Co., Lafayette, USA) or MicroFET 2 (Hoggan Scientific, Salt Lake City, USA) dynamometers; the use of a spring balance was not allowed.

Patient clinical examinations, including baseline imaging assessments, are performed at each site by experienced clinicians (who may be assisted by locally-trained research staff) and documented primarily on paper-based case report forms. Baseline MRI and radiographs are coded and centralized at the University of Basel to ensure data quality control. Operative data are collected electronically by the respective surgeons shortly after surgery. Patients complete questionnaires in their preferred language, which is limited to German, French, Italian or English, either electronically after invitation, by email or on a tablet computer at the site, or otherwise on paper. AEs are documented electronically by the respective surgeons with support from their research staff. Data collected on paper forms are entered electronically at each site or at a central location at the University of Basel based on the agreement made with each site.

Data monitoring

A central project data manager will perform data quality control on all collected data. A flowchart will be created to describe the number of consecutively recruited patients who had an RCR by arthroscopic procedure or had a conversion to an open procedure, and who completed follow-up clinical and imaging examinations as well as self-reported outcome questionnaires. The reasons for patient dropout and loss to follow-up status will be monitored and described. All recorded study parameters will be described using standard descriptive

For peer review only - http://bmjopen.bmj.com/site/about/guidelines.xhtml

statistics; continuous variables will be presented as means with standard deviations and categorical variables as counts with percentages. The variability of data between clinics will be explored to support the identification of outlier data.

Weekly site-specific reports, including the patient enrollment list, expected follow-up timing and identification of missing, erroneous or inconsistent data, are sent to the respective local project staff. Data-related queries will be resolved remotely or by on-site monitoring visits before the final analyses are performed.

There is no plan for auditing project conduct other than via reporting at the annual meetings of the project scientific board.

Data management

Study data will be stored using the REDCap web-based electronic data capture system [71 72] on a server that is hosted at Schulthess Klinik. REDCap conforms with Good Clinical Practice guidelines that provide required features for data protection and integrity, e.g., password-protected access and change tracking.

Study data will be coded and exported from the REDCap system into Stata software (StataCorp LP, College Station, USA) for statistical analyses. Data transformations and analyses will be primarily implemented using Stata and fully documented within Stata programming files. Data subsets will be prepared for analyses using alternative software (e.g. R for prediction models) as appropriate.

All patients with an intraoperatively confirmed RC tear and operated by ARCR will be included in the analyses. Existing missing data will be imputed if the number of missing data is non-negligible or could potentially bias the results and conclusions.

Systematic review of prognostic factors

For peer review only - http://bmjopen.bmj.com/site/about/guidelines.xhtml

For peer review only

A systematic review of prognostic factors for ARCR outcomes is implemented (PROSPERO registration ID: CRD42020199257). Briefly, literature from 2014 to 2020 will be checked to identify longitudinal studies including patients diagnosed with a RC tear. These studies should report the effect of at least one factor on one of the following outcomes: shoulder stiffness, RC tear repair integrity, and shoulder function. Data extraction will follow a pre-defined template and the collected data will be stored within a separate database using REDCap. Data from different studies will be described and may be synthesized depending on the data type and heterogeneity. These data will be used to generate a list of factors most likely to influence our project outcomes and therefore, should be considered for inclusion in the predictive model development process.

Predictive model development

To develop the predictive model(s), the seven steps proposed by Steyerberg et al. [73 74] will be used. The steps comprise: 1) consideration of the research question and initial data inspection, 2) coding of the predictors, 3) model specification, 4) model estimation, 5) evaluation of model performance, 6) internal validation, and 7) model presentation. Depending on the type of outcome, different models will be fitted and evaluated, i.e. multiple regression models for the change in OSS at 6 months and multivariable logistic regression models for shoulder stiffness. Model diagnostics will be performed for all models to check the underlying assumptions.

The prediction of the model(s) will be based on the baseline, operative, and postoperative management variables. Firstly, a subset of the potential prognostic factors will be defined based on whether it is thought to be most predictive. The subset will be selected separately for each outcome by the Delphi method among the investigators, whereby the factors will be noted for their known or potential prognostic value on a 5-point Likert scale from 1 (not important) to 5 (extremely important). These factors, with the highest mean score among investigators, will form the subset.

For peer review only - http://bmjopen.bmj.com/site/about/guidelines.xhtml

We will then use criterion-based procedures (e.g. Akaike Information Criterion [AIC] or adjusted R^2) to select the best set of predictors for the continuous outcome(s) and for the binary outcome, we will use the area under receiving operating characteristics curve (AUC). To assess the predictive performance of the final models as well as the updated version of the prediction models, the calibration plot and discrimination measures will be used. Thereby, apparent performance will be evaluated on the respective development data, and internal validated performance will be determined by bootstrapping. Independent external validation will be estimated by applying the resulting models from the development data set in the respective validation data sets. The resulting models will be used to predict the change of outcome value (i.e. OSS in 6 months) and assess whether a patient will experience the event (i.e. shoulder stiffness).

If we observe missing data, then missing data imputation will be performed using a method that allows for uncertainty in the imputed values (e.g. multiple imputations using chained equation [75]). We will account for the clustering of records within clinics as appropriate.

Adverse events

Occurring AEs other than those listed in the CES as well as events occurring outside the periods defined by the core set will be analyzed separately for consideration of clinical relevance. This analysis will be made by the review committee and project scientific board (PSB) comprising all local project leaders (principal investigators). Recommendations for change of the ARCR CES 1.0 by the PSB will be formulated.

The incidence of AEs, specific individual events and groups of events defined within the ARCR CES 1.0 up to 24 months postoperatively will be displayed as the frequency of patients with an event relative to the number of patients observed, reported together with its 95% Wilson confidence interval. These results will be presented in a summary table together

For peer review only - http://bmjopen.bmj.com/site/about/guidelines.xhtml

with the absolute frequency. Further details on the period of occurrence will be given by stratifying for the time point of event occurrence. We will also stratify AEs according to their severity level and patient relevance. Validation of the postoperative local AE severity classification system will be implemented using previously used methods [76 77].

Patient and public involvement

No patient or member of the public was involved in the design of this cohort study protocol. Enrolled patients will contribute to the evaluation and validation of documented AEs and their severity grading, therefore to a potential revision of the ARCR CES. We are planning to present initial results to patients and the public, and get feedback for further analyses and future model development as well as documentation system in ARCR.

Ethics and dissemination

Research ethics approval

Ethical approval was obtained on April 1st 2020 from the lead ethics committee (EKNZ, Basel Switzerland; ID: 2019-02076).

Protocol amendments

Minor protocol amendments, e.g. database production changes to facilitate monitoring processes or improve outcome assessment by questionnaire, are fully documented. Major amendments, e.g. changes to the patient information sheet and consent form, change of a local project leader or the inclusion of a new project site, will be submitted for approval by the lead ethics committee as required.

Consent or assent

All participants will provide informed written consent prior to being enrolled into the study. The English version of the informed consent form used at the University Hospital of Basel is available as Supplement file 1.

For peer review only - http://bmjopen.bmj.com/site/about/guidelines.xhtml

Confidentiality

Project data will be handled with utmost discretion and can only be accessed by authorized personnel as outlined by a study delegation list created for each project site. Patient data will be coded, i.e. identified by a unique participant number. A participant identification list will be managed and kept in a place (an electronic folder or paper-based form) only accessible to authorized staff at each site.

The project leader affirms and upholds the principle of each patient's right to privacy and that they shall comply with applicable privacy laws. In particular, anonymity of all patients shall be guaranteed when presenting the data at scientific meetings or publishing them in scientific journals.

Declaration of interests

None declared.

Access to data

Project data will be shared at the end of the analysis process by the PSB. The Department of Clinical Research (German Departement Klinische Forschung, DKF) at the University Hospital of Basel will act as an independent data access committee, and will store the data at the time of publication on secure servers, maintained and backed-up by the Information and Communication Technology department at the University Hospital of Basel. Researchers who wish to reuse data will be able to submit a project synopsis to the DKF at dkf.unibas.ch/contact. A data-sharing statement referring researchers to the DKF for data access will be disseminated in the publications. Metadata describing the type, size and content of the data sets will be shared along with the study protocol on the Harvard Dataverse repository available online (https://dataverse.harvard.edu/). Additionally, the case report forms will be uploaded on a medical data models portal (https://medical-data-

For peer review only

For peer review only - http://bmjopen.bmj.com/site/about/guidelines.xhtml

models.org/) and all variables will be annotated by their Unified Medical Language System Concept Unique Identifier to improve accessibility to other clinicians.

Dissemination policy

This project will lead to multiple open-access, peer-reviewed scientific publications, which will be prepared according to international standards (e.g. the STROBE statement [78] for cohort studies; TRIPOD [79] statements for prognostic studies; PRISMA[80] statement for systematic reviews). Publication authorship will regulated according to the guidelines of the Swiss Academies of Arts and Sciences [81]. Results will be submitted for presentation at national and international conferences. In addition, lay summary results will be developed and made available for patients and the public.

Scientific relevance and broader impact

This project initiates the development of personalized risk predictions to support the surgical decision process in ARCR. The consensus CES may become an international reference for the reporting of complications in clinical studies and registries, and may therefore provide a solid metric for the documentation of surgical safety in ARCR. Methodological insight gained from this project will be easily transferable to similar initiatives and thus, may foster the realization of other cohorts on safety and effectiveness outcome in shoulder surgery (e.g. arthroplasty) and orthopedics in general.

For patients affected by RC tears and their surgeons, this study will be the first to provide solid data on the incidence of patient-validated AEs and other core outcomes up to two years after surgical repair based on international consensus COS and CES. This study will allow the investigation of a comprehensive list of potential prognostic factors to generate predictive models for these core outcomes and hence, offer personalized health information to support future patients and surgeons in the decision process for surgery. Outcome predictors and risk calculators are increasingly being developed in numerous medical fields including surgery and orthopedics, and they are in development in the field of ARCR.

For peer review only - http://bmjopen.bmj.com/site/about/guidelines.xhtml

This study will assess the structure and content of the ARCR CES and consolidate its validity in capturing unfavorable events of importance to both patients and surgeons; considering the patient's perspective is an essential step in the development of a COS. Furthermore, the validation of an adapted severity classification of AEs in this study will provide an essential system for assessing surgical morbidity in orthopedics. We expect that the ARCR CES and the event severity classification will become international standards for the reporting of ARCR AEs in clinical studies and registries, and therefore provide a solid metric for the documentation of surgical safety in ARCR.

This study fosters the enterprise in developing a Swiss-wide registry of ARCR, which will allow the ongoing evaluation and prediction of targeted core safety, clinical and patient-reported outcomes. The identification of factors mostly associated with relevant outcomes will facilitate a lean and straightforward documentation process for ARCR patients in Switzerland and abroad.

Acknowledgments

The authors acknowledge the support of Dr. Martina Gosteli, medical librarian at the University of Zurich, for implementing the preliminary literature database search to support this protocol development. The authors would also like to acknowledge the support of Dr. Melissa Wilhelmi, medical writer at Schulthess Klinik, Zurich, Switzerland, for manuscript proofreading.

For peer review only - http://bmjopen.bmj.com/site/about/guidelines.xhtml

References

1. Schmidt CC, Morrey BF. Management of full-thickness rotator cuff tears: appropriate use criteria. *J Shoulder Elbow Surg* 2015;24(12):1860-7. doi: 10.1016/j.jse.2015.05.042
2. Carr AJ, Cooper CD, Campbell MK, et al. Clinical effectiveness and cost-effectiveness of open and arthroscopic rotator cuff repair [the UK Rotator Cuff Surgery (UKUFF) randomised trial]. *Health Technol Assess* 2015;19(80):1-218. doi: 10.3310/hta19800 [published Online First: 2015/10/16]
3. Moosmayer S, Lund G, Seljom US, et al. At a 10-Year Follow-up, Tendon Repair Is Superior to Physiotherapy in the Treatment of Small and Medium-Sized Rotator Cuff Tears. *J Bone Joint Surg Am* 2019;101(12):1050-60. doi: 10.2106/JBJS.18.01373 [published Online First: 2019/06/21]
4. Grobet C, Audige L, Eichler K, et al. Cost-Utility Analysis of Arthroscopic Rotator Cuff Repair: A Prospective Health Economic Study Using Real-World Data. *Arthrosc Sports Med Rehabil* 2020;2(3):e193-e205. doi: 10.1016/j.asmr.2020.02.001 [published Online First: 2020/06/18]
5. Piper CC, Hughes AJ, Ma Y, et al. Operative versus nonoperative treatment for the management of full-thickness rotator cuff tears: a systematic review and meta-analysis. *J Shoulder Elbow Surg* 2018;27(3):572-76. doi: 10.1016/j.jse.2017.09.032 [published Online First: 2017/11/25]
6. Ensor KL, Kwon YW, Dibeneditto MR, et al. The rising incidence of rotator cuff repairs. *J Shoulder Elbow Surg* 2013;22(12):1628-32. doi: 10.1016/j.jse.2013.01.006
7. Iyengar JJ, Samagh SP, Schairer W, et al. Current trends in rotator cuff repair: surgical technique, setting, and cost. *Arthroscopy* 2014;30(3):284-8. doi: 10.1016/j.arthro.2013.11.018
8. Colvin AC, Egorova N, Harrison AK, et al. National trends in rotator cuff repair. *J Bone Joint Surg Am* 2012;94(3):227-33. doi: 10.2106/JBJS.J.00739
9. Jung C, Buchmann S, Flury M. Die arthroskopische Rotatorenmanschetten-Rekonstruktion - Von der Aussnahme zum Standard. Swiss Journal of Medicine and Medical Technology (Swiss Med), 2012:35-39.
10. Keener JD, Patterson BM, Orvets N, et al. Degenerative Rotator Cuff Tears: Refining Surgical Indications Based on Natural History Data. *J Am Acad Orthop Surg* 2019;27(5):156-65. doi: 10.5435/JAAOS-D-17-00480 [published Online First: 2018/10/20]
11. Randelli P, Spennacchio P, Ragone V, et al. Complications associated with arthroscopic rotator cuff repair: a literature review. *Musculoskelet Surg* 2012;96(1):9-16. doi: 10.1007/s12306-011-0175-y

For peer review only

For peer review only - http://bmjopen.bmj.com/site/about/guidelines.xhtml

12. Felsch Q, Mai V, Durchholz H, et al. Complications within 6 months after arthroscopic rotator cuff repair: registry-based evaluation according to a core event set and severity grading. *Arthroscopy* 2020:in Press. doi: 10.1016/j.arthro.2020.08.010 [published Online First: 2020/08/25]

13. Muller AM, Flury M, Alsayed HN, et al. Influence of patient and diagnostic parameters on reported retear rates after arthroscopic rotator cuff repair. *Knee Surg Sports Traumatol Arthrosc* 2017 doi: 10.1007/s00167-017-4481-2

14. Yoo JH, Cho NS, Rhee YG. Effect of postoperative repair integrity on health-related quality of life after rotator cuff repair: healed versus retear group. *Am J Sports Med* 2013;41(11):2637-44. doi: 10.1177/0363546513499152 [published Online First: 2013/08/15]

15. Robinson HA, Lam PH, Walton JR, et al. The effect of rotator cuff repair on early overhead shoulder function: a study in 1600 consecutive rotator cuff repairs. *Journal of Shoulder & Elbow Surgery* 2017;26(1):20-29. doi: 10.1016/j.jse.2016.05.022

16. Huberty DP, Schoolfield JD, Brady PC, et al. Incidence and treatment of postoperative stiffness following arthroscopic rotator cuff repair. *Arthroscopy : the journal of arthroscopic & related surgery : official publication of the Arthroscopy Association of North America and the International Arthroscopy Association* 2009;25(8):880-90. doi: 10.1016/j.arthro.2009.01.018 [published Online First: 2009/08/12]

17. Denard PJ, Ladermann A, Burkhart SS. Prevention and management of stiffness after arthroscopic rotator cuff repair: systematic review and implications for rotator cuff healing. *Arthroscopy* 2011;27(6):842-8. doi: 10.1016/j.arthro.2011.01.013

18. Itoi E, Arce G, Bain GI, et al. Shoulder Stiffness: Current Concepts and Concerns. *Arthroscopy : the journal of arthroscopic & related surgery : official publication of the Arthroscopy Association of North America and the International Arthroscopy Association* 2016;32(7):1402-14. doi: 10.1016/j.arthro.2016.03.024 [published Online First: 2016/05/18]

19. Audige L, Blum R, Muller AM, et al. Complications Following Arthroscopic Rotator Cuff Tear Repair: A Systematic Review of Terms and Definitions With Focus on Shoulder Stiffness. *Orthopaedic Journal of Sports Medicine* 2015;3(6) doi: 10.1177/2325967115587861

20. Gargon E, Gurung B, Medley N, et al. Choosing important health outcomes for comparative effectiveness research: a systematic review. *PLoS One* 2014;9(6):e99111. doi: 10.1371/journal.pone.0099111

21. Buchbinder R, Page MJ, Huang H, et al. A Preliminary Core Domain Set for Clinical Trials of Shoulder Disorders: A Report from the OMERACT 2016 Shoulder Core Outcome Set Special Interest Group. *J Rheumatol* 2017;44(12):1880-83. doi: 10.3899/jrheum.161123 [published Online First: 2017/01/17]

For peer review only - http://bmjopen.bmj.com/site/about/guidelines.xhtml

22. Page MJ, Huang H, Verhagen AP, et al. Identifying a core set of outcome domains to measure in clinical trials for shoulder disorders: a modified Delphi study. *RMD Open* 2016;2(2):e000380. doi: 10.1136/rmdopen-2016-000380 [published Online First: 2017/01/27]
23. Audige L, Flury M, Muller AM, et al. Complications associated with arthroscopic rotator cuff tear repair: definition of a core event set by Delphi consensus process. *J Shoulder Elbow Surg* 2016;25(12):1907-17. doi: 10.1016/j.jse.2016.04.036
24. Felsch Q, Mai V, Durchholz H, et al. Complications within 6 months after arthroscopic rotator cuff repair: registry-based evaluation according to a core event set and severity grading. *Arthroscopy : the journal of arthroscopic & related surgery : official publication of the Arthroscopy Association of North America and the International Arthroscopy Association* 2020 doi: 10.1016/j.arthro.2020.08.010 [published Online First: 2020/08/25]
25. Steyerberg EW, Moons KG, van der Windt DA, et al. Prognosis Research Strategy (PROGRESS) 3: prognostic model research. *PLoS Med* 2013;10(2):e1001381. doi: 10.1371/journal.pmed.1001381 [published Online First: 2013/02/09]
26. Sepucha KR, Atlas SJ, Chang Y, et al. Informed, Patient-Centered Decisions Associated with Better Health Outcomes in Orthopedics: Prospective Cohort Study. *Med Decis Making* 2018;38(8):1018-26. doi: 10.1177/0272989X18801308 [published Online First: 2018/11/08]
27. Guo W, Jin J, Paynabar K, et al. A decision support system on surgical treatments for rotator cuff tears. *IIE Transactions on Healthcare Systems Engineering* 2015;5(3):197-210. doi: 10.1080/19488300.2015.1065935
28. Kwon J, Kim SH, Lee YH, et al. The Rotator Cuff Healing Index: A New Scoring System to Predict Rotator Cuff Healing After Surgical Repair. *Am J Sports Med* 2019;47(1):173-80. doi: 10.1177/0363546518810763 [published Online First: 2018/11/30]
29. Jeong HY, Kim HJ, Jeon YS, et al. Factors predictive of healing in large rotator cuff tears: Is it possible to predict retear preoperatively? *The American journal of sports medicine* 2018;46(7):1693-700.
30. Frangiamore S, Dornan GJ, Horan MP, et al. Predictive Modeling to Determine Functional Outcomes After Arthroscopic Rotator Cuff Repair. *Am J Sports Med* 2020;48(7):1559-67. doi: 10.1177/0363546520914632 [published Online First: 2020/05/15]
31. Koorevaar RC, van't Riet E, Ipskamp M, et al. Incidence and prognostic factors for postoperative frozen shoulder after shoulder surgery: a prospective cohort study. *Archives of orthopaedic and trauma surgery* 2017;137(3):293-301.

For peer review only

For peer review only - http://bmjopen.bmj.com/site/about/guidelines.xhtml

32. Fermont AJ, Wolterbeek N, Wessel RN, et al. Prognostic factors for recovery after arthroscopic rotator cuff repair: a prognostic study. *J Shoulder Elbow Surg* 2015;24(8):1249-56. doi: 10.1016/j.jse.2015.04.013

33. Lambers Heerspink FO, Dorrestijn O, van Raay JJ, et al. Specific patient-related prognostic factors for rotator cuff repair: a systematic review. *J Shoulder Elbow Surg* 2014;23(7):1073-80. doi: 10.1016/j.jse.2014.01.001

34. McElvany MD, McGoldrick E, Gee AO, et al. Rotator cuff repair: published evidence on factors associated with repair integrity and clinical outcome. *American Journal of Sports Medicine* 2015;43(2):491-500. doi: 10.1177/0363546514529644

35. Saccomanno MF, Sircana G, Cazzato G, et al. Prognostic factors influencing the outcome of rotator cuff repair: a systematic review. *Knee Surgery, Sports Traumatology, Arthroscopy* 2016;24(12):3809-19.

36. Raman J, Walton D, MacDermid JC, et al. Predictors of outcomes after rotator cuff repair-A meta-analysis. *J Hand Ther* 2017;30(3):276-92. doi: 10.1016/j.jht.2016.11.002

37. Dindo D, Demartines N, Clavien PA. Classification of surgical complications: a new proposal with evaluation in a cohort of 6336 patients and results of a survey. *Ann Surg* 2004;240(2):205-13. [published Online First: 2004/07/27]

38. Lafosse L, Jost B, Reiland Y, et al. Structural integrity and clinical outcomes after arthroscopic repair of isolated subscapularis tears. *J Bone Joint Surg Am* 2007;89(6):1184-93. doi: 10.2106/JBJS.F.00007

39. Dawson J, Rogers K, Fitzpatrick R, et al. The Oxford shoulder score revisited. *Arch Orthop Trauma Surg* 2009;129(1):119-23.

40. Huber W, Hofstaetter JG, Hanslik-Schnabel B, et al. The German version of the Oxford Shoulder Score--cross-cultural adaptation and validation. *Arch Orthop Trauma Surg* 2004;124(8):531-6.

41. Murena L, Vulcano E, D'Angelo F, et al. Italian cross-cultural adaptation and validation of the Oxford Shoulder Score. *J Shoulder Elbow Surg* 2010;19(3):335-41. doi: 10.1016/j.jse.2009.07.068 [published Online First: 2009/11/10]

42. Olley LM, Carr AJ. The use of a patient-based questionnaire (the Oxford Shoulder Score) to assess outcome after rotator cuff repair. *Ann R Coll Surg Engl* 2008;90(4):326-31. doi: 10.1308/003588408X285964 [published Online First: 2008/05/22]

43. Xu S, Chen JY, Lie HME, et al. Minimal Clinically Important Difference of Oxford, Constant, and UCLA shoulder score for arthroscopic rotator cuff repair. *J* 2020;19:21-27. doi: 10.1016/j.jor.2019.11.037 [published Online First: 2020/02/06]

44. Allom R, Colegate-Stone T, Gee M, et al. Outcome analysis of surgery for disorders of the rotator cuff: a comparison of subjective and objective scoring tools. *J Bone Joint*

For peer review only

For peer review only - http://bmjopen.bmj.com/site/about/guidelines.xhtml

Surg Br 2009;91(3):367-73. doi: 10.1302/0301-620X.91B3.20714 [published Online First: 2009/03/05]

45. Constant CR, Gerber C, Emery RJ, et al. A review of the Constant score: modifications and guidelines for its use. *J Shoulder Elbow Surg* 2008;17(2):355-61. doi: 10.1016/j.jse.2007.06.022
46. Gilbart MK, Gerber C. Comparison of the subjective shoulder value and the Constant score. *J Shoulder Elbow Surg* 2007;16(6):717-21. doi: 10.1016/j.jse.2007.02.123 [published Online First: 2007/12/07]
47. Cappelleri JC, Bushmakin AG, McDermott AM, et al. Psychometric properties of a single-item scale to assess sleep quality among individuals with fibromyalgia. *Health and quality of life outcomes* 2009;7(1):54.
48. Potter MQ, Wylie JD, Greis PE, et al. Psychological distress negatively affects self-assessment of shoulder function in patients with rotator cuff tears. *Clin Orthop Relat Res* 2014;472(12):3926-32. doi: 10.1007/s11999-014-3833-1
49. Makhni EC, Meadows M, Hamamoto JT, et al. Patient Reported Outcomes Measurement Information System (PROMIS) in the upper extremity: the future of outcomes reporting? *Journal of Shoulder and Elbow Surgery* 2017;26(2):352-57. doi: 10.1016/j.jse.2016.09.054
50. Tubach F, Wells GA, Ravaud P, et al. Minimal clinically important difference, low disease activity state, and patient acceptable symptom state: methodological issues. *J Rheumatol* 2005;32(10):2025-9. [published Online First: 2005/10/06]
51. Slankamenac K, Graf R, Barkun J, et al. The comprehensive complication index: a novel continuous scale to measure surgical morbidity. *Ann Surg* 2013;258(1):1-7. doi: 10.1097/SLA.0b013e318296c732
52. Sugaya H, Maeda K, Matsuki K, et al. Functional and structural outcome after arthroscopic full-thickness rotator cuff repair: single-row versus dual-row fixation. *Arthroscopy* 2005;21(11):1307-16. doi: 10.1016/j.arthro.2005.08.011 [published Online First: 2005/12/06]
53. Collin P, Yoshida M, Delarue A, et al. Evaluating postoperative rotator cuff healing: Prospective comparison of MRI and ultrasound. *Orthop Traumatol Surg Res* 2015;101(6 Suppl):S265-8. doi: 10.1016/j.otsr.2015.06.006 [published Online First: 2015/08/19]
54. Herdman M, Gudex C, Lloyd A, et al. Development and preliminary testing of the new five-level version of EQ-5D (EQ-5D-5L). *Qual Life Res* 2011;20(10):1727-36. doi: 10.1007/s11136-011-9903-x [published Online First: 2011/04/12]
55. Moor BK, Wieser K, Slankamenac K, et al. Relationship of individual scapular anatomy and degenerative rotator cuff tears. *J Shoulder Elbow Surg* 2014;23(4):536-41. doi: 10.1016/j.jse.2013.11.008

For peer review only

For peer review only - http://bmjopen.bmj.com/site/about/guidelines.xhtml

56. Werner CM, Conrad SJ, Meyer DC, et al. Intermethod agreement and interobserver correlation of radiologic acromiohumeral distance measurements. *J Shoulder Elbow Surg* 2008;17(2):237-40. doi: 10.1016/j.jse.2007.06.002

57. Thomazeau H, Rolland Y, Lucas C, et al. Atrophy of the supraspinatus belly assessment by MRI in 55 patients with rotator cuff pathology. *Acta orthopaedica Scandinavica* 1996;67(3):264-68.

58. Zanetti M, Gerber C, Hodler J. Quantitative assessment of the muscles of the rotator cuff with magnetic resonance imaging. *Investigative radiology* 1998;33(3):163-70.

59. Schiefer M, Mendonca R, Magnanini MM, et al. Intraobserver and interobserver agreement of Goutallier classification applied to magnetic resonance images. *J Shoulder Elbow Surg* 2015;24(8):1314-21. doi: 10.1016/j.jse.2015.02.011

60. Goutallier D, Postel JM, Bernageau J, et al. Fatty muscle degeneration in cuff ruptures. Pre- and postoperative evaluation by CT scan. *Clin Orthop Relat Res* 1994(304):78-83. [published Online First: 1994/07/01]

61. Patte D. Classification of rotator cuff lesions. *Clin Orthop Relat Res* 1990(254):81-6. [published Online First: 1990/05/01]

62. Okoroha KR, Mehran N, Duncan J, et al. Characterization of Rotator Cuff Tears: Ultrasound Versus Magnetic Resonance Imaging. *Orthopedics* 2016:1-7. doi: 10.3928/01477447-20161013-04

63. Zumstein MA, Ladermann A, Raniga S, et al. The biology of rotator cuff healing. *Orthop Traumatol Surg Res* 2016 doi: 10.1016/j.otsr.2016.11.003 [published Online First: 2017/01/04]

64. Audige L, Goldhahn S, Daigl M, et al. How to document and report orthopedic complications in clinical studies? A proposal for standardization. *Arch Orthop Trauma Surg* 2014;134(2):269-75. doi: 10.1007/s00402-011-1384-4

65. Rosenthal R, Hoffmann H, Clavien PA, et al. Definition and Classification of Intraoperative Complications (CLASSIC): Delphi Study and Pilot Evaluation. *World J Surg* 2015;39(7):1663-71. doi: 10.1007/s00268-015-3003-y

66. Dell-Kuster S, Gomes NV, Gawria L, et al. Prospective validation of classification of intraoperative adverse events (ClassIntra): international, multicentre cohort study. *BMJ* 2020;370:m2917. doi: 10.1136/bmj.m2917 [published Online First: 2020/08/28]

67. Flury M, Kolling C, Grobet C, et al. Implementation of a local outcome register for arthroscopic rotator cuff tear repair. *Obere Extremität* 2014;10(1):33-40. doi: 10.1007/s11678-014-0299-4

68. Vach W. Regression models as a tool in medical research. Boca Raton, FL: CRC Press, Taylor & Francis Group 2013.

69. Peduzzi P, Concato J, Kemper E, et al. A simulation study of the number of events per variable in logistic regression analysis. *J Clin Epidemiol* 1996;49(12):1373-9.

For peer review only - http://bmjopen.bmj.com/site/about/guidelines.xhtml

70. Hemingway H, Croft P, Perel P, et al. Prognosis research strategy (PROGRESS) 1: a framework for researching clinical outcomes. *BMJ* 2013;346:e5595. doi: 10.1136/bmj.e5595

71. Harris PA, Taylor R, Thielke R, et al. Research electronic data capture (REDCap)--a metadata-driven methodology and workflow process for providing translational research informatics support. *Journal of biomedical informatics* 2009;42(2):377-81. doi: 10.1016/j.jbi.2008.08.010 [published Online First: 2008/10/22]

72. REDCap web-based Electronic Data Capture system 2017 [Available from: www.project-redcap.org accessed 15.03.2017 2017.

73. Steyerberg EW, Vergouwe Y. Towards better clinical prediction models: seven steps for development and an ABCD for validation. *European Heart Journal* 2014;35(29):1925-31. doi: 10.1093/eurheartj/ehu207

74. Steyerberg EW. Clinical prediction models, 2009.

75. Team RC. R: A language and environment for statistical computing. : R Foundation for Statistical Computing, Vienna, Austria; 2016 [Available from: www.R-project.org.

76. Porembka MR, Hall BL, Hirbe M, et al. Quantitative weighting of postoperative complications based on the accordion severity grading system: demonstration of potential impact using the american college of surgeons national surgical quality improvement program. *Journal of the American College of Surgeons* 2010;210(3):286-98. doi: 10.1016/j.jamcollsurg.2009.12.004

77. de la Rosette JJ, Opondo D, Daels FP, et al. Categorisation of complications and validation of the Clavien score for percutaneous nephrolithotomy. *European urology* 2012;62(2):246-55.

78. Vandenbroucke JP, Von Elm E, Altman DG, et al. Strengthening the Reporting of Observational Studies in Epidemiology (STROBE): explanation and elaboration. *PLoS Med* 2007;4(10):e297.

79. Collins GS, Reitsma JB, Altman DG, et al. Transparent Reporting of a multivariable prediction model for Individual Prognosis or Diagnosis (TRIPOD): the TRIPOD statement. *Ann Intern Med* 2015;162(1):55-63. doi: 10.7326/M14-0697 [published Online First: 2015/01/07]

80. Moher D, Liberati A, Tetzlaff J, et al. Preferred reporting items for systematic reviews and meta-analyses: the PRISMA statement. *PLoS Med* 2009;6(7):e1000097. doi: 10.1371/journal.pmed.1000097 [published Online First: 2009/07/22]

81. Hess CW, Brückner C, Kaiser T, et al. Authorship in scientific publications: analysis and recommendations. *Swiss medical weekly* 2015;145

For peer review only

For peer review only - http://bmjopen.bmj.com/site/about/guidelines.xhtml

911 **Figure legend**

913 **Figure 1**: Flowchart of study procedures

914 w = week; mo = month; FU = follow-up; MRI = Magnetic Resonance Imaging; CT = Arthro-
915 Computer Tomography (*if MRI not possible); Motion = Shoulder range of motion; CS =
916 Constant Score; RC = Rotator Cuff; AE = Adverse Event; Rehab. = recall on postoperative
917 rehabilitation; DE = German; FR = French; IT = Italian; EN = English; NRS = Numeric Rating
918 Scale; OSS = Oxford Shoulder Score; SSV = Subjective Shoulder Value; EQ-5D-5L =
919 European Quality of Life 5 Dimensions 5 Level questionnaire; CES = Core Event Set; AE
920 survey = surgeons and patients survey regarding AE severity (sev)

For peer review only

For peer review only - http://bmjopen.bmj.com/site/about/guidelines.xhtml

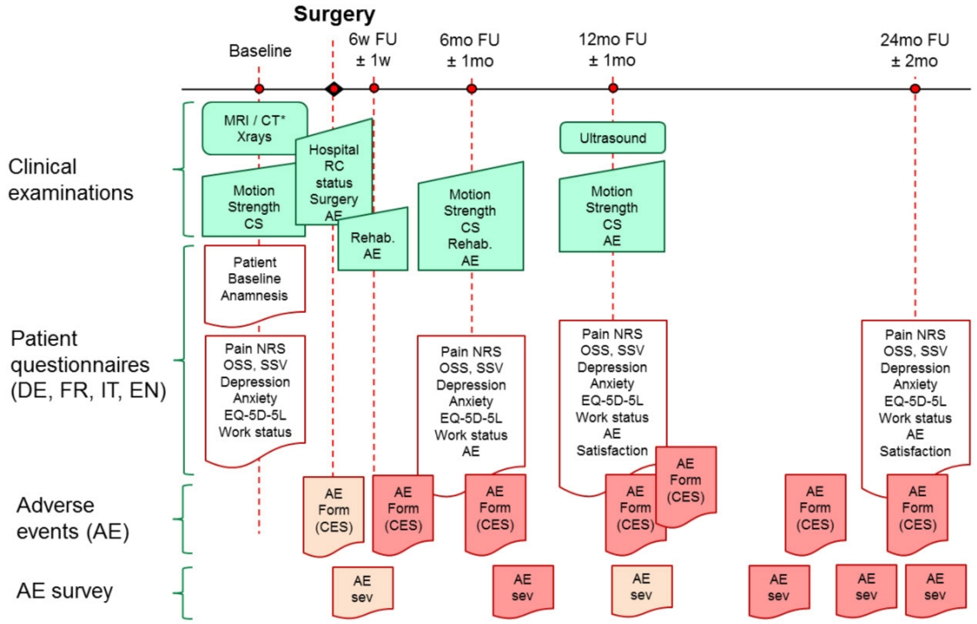

Figure 1: Flowchart of study procedures
w = week; mo = month; FU = follow-up; MRI = Magnetic Resonance Imaging; CT = Arthro-Computer Tomography (*if MRI not possible); Motion = Shoulder range of motion; CS = Constant Score; RC = Rotator Cuff; AE = Adverse Event; Rehab. = recall on postoperative rehabilitation; DE = German; FR = French; IT = Italian; EN = English; NRS = Numeric Rating Scale; OSS = Oxford Shoulder Score; SSV = Subjective Shoulder Value; EQ-5D-5L = European Quality of Life 5 Dimensions 5 Level questionnaire; CES = Core Event Set; AE survey = surgeons and patients survey regarding AE severity (sev)

342x220mm (96 x 96 DPI)

Supplement file 1

**Article title**	Protocol for the ARCR_Pred cohort-study: Swiss-wide multicenter evaluation and prediction of core outcomes in arthroscopic rotator cuff repair
**Journal name**	BMJ Open Access
**Author names**	Laurent Audigé, Heiner C. Bucher, Soheila Aghlmandi, Thomas Stojanov, David Schwappach, Sabina Hunziker, Christian Candrian, Gregory Cunningham, Holger Durchholz, Karim Eid, Matthias Flury, Bernhard Jost, Alexandre Lädermann, Beat Moor, Philipp Moroder, Claudio Rosso, Michael Schär, Markus Scheibel, Christoph Spormann, Thomas Suter, Karl Wieser, Matthias A. Zumstein, ARCR_Pred Study Group*, Andreas Müller
**Corresponding author**	Laurent Audigé, Schulthess Klinik, CH-8008 Zurich, Switzerland
**e-mail address**	laurent.audige@kws.ch

* Members of the ARCR_Pred study group are listed in the main publication of this project protocol.

Patient information sheet and informed consent form (English version) for the project site “University Hospital of Basel” (USB)

For peer review only

Universitätsspital Basel

Engl. Surgical safety and effectiveness in orthopaedics: evaluation of an international consensus core set of adverse events in arthroscopic rotator cuff repair

This project has been organised by: PD Dr. Andreas Müller (project leader; Deputy Consultant of Orthopaedics, Shoulder/Elbow team leader, Orthopaedic and Traumatology Department, University Hospital of Basel) and Prof. Dr. Laurent Audigé (project manager; Research Associate, Orthopaedic and Traumatology Department, University Hospital of Basel and Head of Research Group Upper Extremities, Department of Research and Development, Schulthess Clinic, Zurich)

Sponsor: University Hospital of Basel, PD Dr. Andreas Müller, Deputy Consultant of Orthopaedics and Traumatology

Dear Sir / Madam,

We would like to ask you if you are interested in participating in a research project. The planned project is first presented as a short summary in the table provided below followed by a more detailed description.

Summary of the project

1	**Aim of the project**<br>The research project will investigate how easily the most important results are predicted following surgical interventions to treat tears of the shoulder muscle (rotator cuff) tendons. In order to do this, we will use and assess a predefined set of adverse events (such as frozen shoulder or persistent pain) in practice.
2	**Choice**<br>You are an adult suffering from a tear of the shoulder muscle tendons, which can be treated with an initial, minimally invasive (i.e. arthroscopic) surgical intervention. That is why we are sending you this information leaflet.
3	**General information about the project**<br>The collection of clinical data following surgical interventions is very important, and helps to support an established decision-making process within the orthopaedic field. We are carrying out this project so that we can better evaluate and predict the results following the repair of shoulder muscle tendon tears. This evaluation is being carried out on a representative number of male and female patients in Switzerland.<br>Patient safety is essential here. A list of possible adverse events following the arthroscopic intervention was recently defined by a group of more than 80 experts in the field. We would now like to better understand the development of these adverse events from the patient's point of view.
4	**Procedure**<br>This project will last four years. In total, 970 patients from various clinics in Switzerland and one in Germany will be included in the study within the first year. Various examinations (e.g. measurements of range of motion and strength) will be carried out in the clinic before the operation (= preoperative) and then at the 6- and 12-month postoperative time points. At these times and at 2 years post-surgery, you will receive a questionnaire to complete. We will use ultrasound to check how the tendon is healing twelve months after the operation. Any adverse events will be evaluated independently by the doctor and the patient in question.

For peer review only - http://bmjopen.bmj.com/site/about/guidelines.xhtml

Universitätsspital Basel

5	**Usefulness**<br>You will gain no personal benefit from participating in the project. However, the results could be important to others who have the same condition.
6	**Rights**<br>You decide voluntarily whether you want to participate in this project or not. Your decision does not affect your medical treatment/care and you do not have to justify it.
7	**Duties**<br>If you participate, we ask you to adhere to certain requirements (e.g. attending visits and completing the questionnaires).
8	**Risks**<br>You are not exposed to any additional risk by participating in the project.
9	**Outcomes**<br>You will be informed of new results during the project. We will promptly inform you of any additional findings (known as incidental findings) that are detected during the regular study examinations, which may affect your health directly. Any further course of action will then be discussed in detail with you.
10	**Confidentiality of data and samples**<br>We collect your personal and medical data from you. The Swiss National Science Foundation supports the exchange and reuse of research data. The data will be used for other projects if you give your separate consent. We comply with all legal data protection regulations. All parties involved are bound by confidentiality.
11	**Withdrawal**<br>You can withdraw from the project at any time and no longer participate. The data collected so far are still being evaluated.
12	**Indemnity**<br>You will not receive any compensation for participating in the study. Neither your health insurance provider nor you will incur any additional costs from your participation in the study.
13	**Liability**<br>The liability insurance of the project management is liable for any damages within the scope of the project.
14	**Funding**<br>The project is paid for by the Swiss National Science Foundation.
15	**Contact person:**<br>You can receive information on all your questions at any time:<br><br>PD Dr. Andreas Müller, Senior Consultant, Head Shoulder and Elbow,<br>University Hospital of Basel, Spitalstrasse 21, CH-403 Basel<br>Tel 061 315 25 17 , Email A.Mueller@usb.ch

For peer review only - http://bmjopen.bmj.com/site/about/guidelines.xhtml

Universitätsspital Basel

More detailed information

1. Aim of the project

The aim of this project is to investigate how the most important results (for example, the occurrence of adverse events or shoulder function) are easily predicted following a surgical intervention to treat tears of the shoulder muscle tendons. Furthermore, we want to investigate how well a predefined set of adverse events, which could occur as part of such a surgical intervention, corresponds to what actually occurs in practice.

2. Choice

Participation is open to anyone with a torn tendon in the shoulder muscles, which can be repaired with minimally invasive (i.e. arthroscopic) surgery.

Important: This must be the first intervention on the shoulder in question.

Participation is not open to anyone for whom a detailed medical examination would not be possible or who cannot be called back for check-ups within the follow-up period (e.g. if they live outside Switzerland). Underage persons should also not participate.

3. General information about the project

This project will be carried out in accordance with the laws of Switzerland. The responsible ethics committee has reviewed and approved this project

The collection of clinical data following surgical interventions is very important. You will help:

- to assess the effectiveness and safety of the intervention,
- to make comparisons with other methods,
- to support an established decision-making process within the orthopaedic field.

The study intends to document the most important events following arthroscopic interventions, especially in terms of:

- safety (occurrence of adverse events),
- healing of the tendon repair,
- shoulder pain and function,
- the general state of health and quality of life as well as
- patient satisfaction.

Patients' socio-demographic characteristics, examination parameters and treatment parameters will be investigated to enable a prediction of these events to be made.

In most areas of orthopaedics, there is currently no international standard for the description of adverse events (often known as complications) resulting from surgical interventions. In shoulder orthopaedics, such a list of events does not exist for arthroscopic interventions used in the treatment of tears of the shoulder muscle tendons (rotator cuff). For this reason, the Shoulder and Elbow Surgery research group at the Schulthess Clinic, Zurich, working together with the Orthopaedic and Traumatology Department of the University Hospital of Basel and over 80 experts working in the field of arthroscopy for rotator cuff tears, have defined a list of possible adverse events.

The aim is to apply and evaluate this predefined list of events. Each event will be evaluated independently by the doctor and the patient in question.

For peer review only - http://bmjopen.bmj.com/site/about/guidelines.xhtml

Universitätsspital Basel

This project is supported by the Swiss National Science Foundation and will last four years. In total, 970 patients will be included in the study within the first year. These patients will be recruited from various clinics in Switzerland and one clinic in Germany. At the University Hospital of Basel about 60 patients are recruited.

4. Procedure

The following diagram shows the course of study events from the time of diagnosis to the follow-up appointment at two years post-surgery.

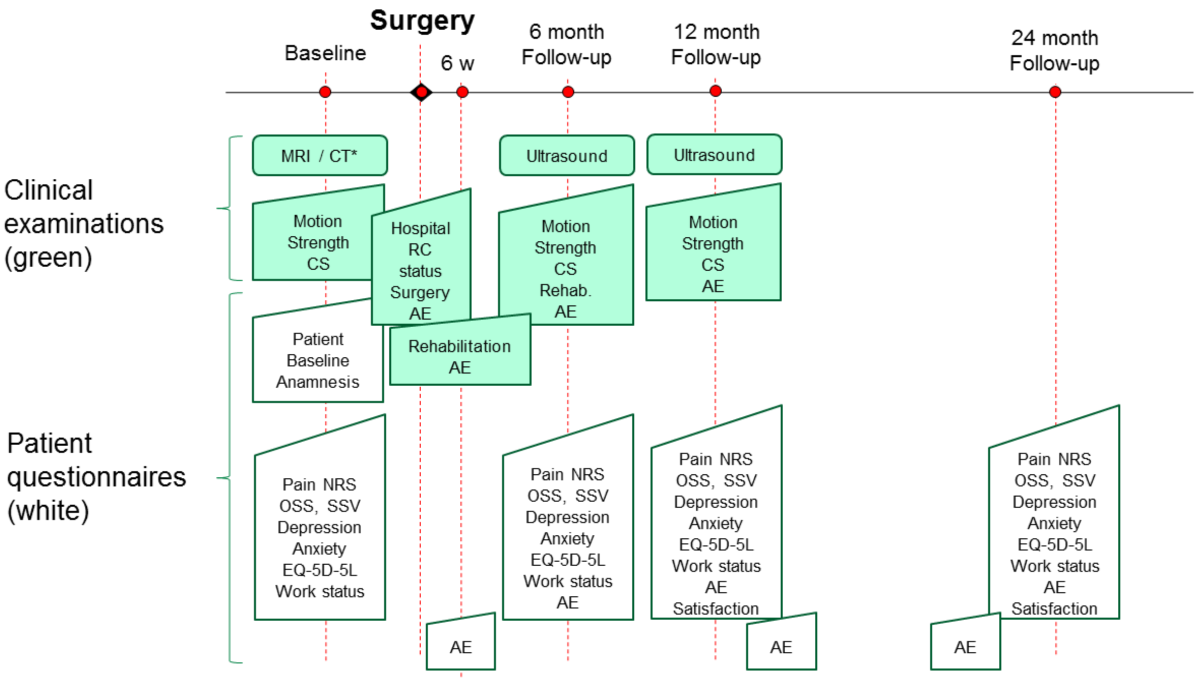

6 W = 6-week follow-up; AE = adverse events

Figure 1: Schematic representation of the study's progress

For your diagnosis, you will be examined by the doctor using various methods (e.g. functional testing, radiological and magnetic resonance imaging (= MRI)) during the first consultation. If you agree to participate in the project, you will receive a questionnaire, which will ask you to provide your personal details and estimate your current level of functional ability in everyday life. Shortly before the operation, your surgeon will record detailed information about your shoulder injury and the surgical intervention. Six weeks after the operation, your surgeon will ask you about your pain levels, rehabilitation programme and the occurrence of any adverse events.

During further follow-up appointments (at 6 and 12 months post-surgery), various examinations (e.g. measurements of range of motion and strength, see figure 1) are routinely performed in the clinic. An independent examiner will carry out an ultrasound routinely 12 months after the operation to document how the tendon is healing. You will also receive a questionnaire to complete. If you wish, you can complete this questionnaire at home - either on paper or electronically by following an invitation link that will be emailed to you. At the end of this document, you will be asked to provide your email address, if you do prefer to receive the electronic version of the questionnaire. Various questionnaires are already routinely recorded at the University Hospital of Basel.

For peer review only - http://bmjopen.bmj.com/site/about/guidelines.xhtml

Universitätsspital Basel

Two years after the operation, you will be sent the questionnaire again by post or email (no further check-ups will take place at the clinic). This takes place within the framework of the project. If you have experienced one or more adverse events, the questionnaire will ask you to rate each event by severity and by its relationship to your treatment. The study doctors will evaluate all reported events by severity without knowing the patient, to whom these relate or the clinic in which the events occurred.

Each visit to the clinic (before surgery and 6- and 12-months post-surgery) will last 40 to 60 minutes including all the examinations. Completing the patient questionnaire will take an additional 20 to 30 minutes. An adverse event tends to be evaluated in only a few minutes.

We may have to exclude you from this project prematurely. This can occur if no tear of the rotator cuff can be confirmed intraoperatively (i.e. during the operation) (this occurs very rarely) or if a tendon repair is not possible without additional interventions on the shoulder (a so-called irreparable tear). The study doctor will inform you of any such exclusion from the project.

5. Usefulness

You will not personally benefit from participating in the project. The results can be important for others who have the same condition. As described above, the study aims to enable better evaluation and prediction of the risk of adverse events and the effectiveness of a surgical intervention. We want to support the decision-making processes of future interventions of this kind.

6. Rights

You're volunteering. If you do not want to participate or later withdraw your participation, you do not have to justify this. Your medical treatment/care is guaranteed regardless of your decision. You may ask questions about participation and the project at any time. Please contact the person named at the end of this information.

7. Duties

As a participant, it is necessary that you

- adhere to the necessary specifications and requirements of the project management.
- inform your investigator/project management about the course of the disease and report new symptoms, new complaints and changes in well-being.
- inform your investigator/project management about the simultaneous treatment and therapy with another doctor and about taking medication.

8. Risks

You are not exposed to any additional risk by participating in the project.

9. Outcomes

The investigator/project manager will inform you during the project about any new findings that may affect the benefit or your safety and thus your consent to participate. You will be informed of random findings which may contribute to the prevention, detection or treatment of existing or future diseases.

10. Confidentiality of data and samples

Your personal and medical data will be collected for this project. Very few professionals will see your unencrypted data, and only to perform tasks within the scope of the project. Data collection for study purposes is encrypted. Encryption means that all reference data that could identify you (name, date of birth) are deleted and replaced by a key. The key list always remains in the institution/hospital. Those who do not know the key cannot therefore draw any conclusions about you. In the

For peer review only - http://bmjopen.bmj.com/site/about/guidelines.xhtml

Universitätsspital Basel

case of a publication, the summarised data cannot therefore be traced back to you as an individual. Your name will never appear on the Internet or in any publication. Sometimes there is a requirement in a journal for publication that individual data (so-called raw data) must be transmitted. If individual data must be transmitted, then the data is always encrypted and cannot be traced back to you as a person. All persons who have access to your data within the scope of the project are subject to confidentiality. The requirements of data protection are adhered to and you as a participating person have the right to inspect your data at any time.

If data are stored on site, it is a database for research purposes.

Each centre will encode and save the data centrally in the project database (server location: Schulthess Clinic, Zurich). The data will be deleted 10 years after the end of the project.
It is possible that your data may be used for other investigations (projects) at a later date or that they may be sent to another databank in Switzerland for investigations (further use) not yet defined in more detail. This other database must meet the same standards as the database for this project. For this further use we ask you to sign a further declaration of consent at the very end of this document.

This project may be reviewed by the relevant ethics committee or by the institution that initiated the project. The project manager may need to disclose your personal and medical information for such checks. All persons must maintain absolute confidentiality. We comply with all data protection regulations and will not make your name public either in a publication or on the Internet.

It is possible that your aftercare physician will be contacted to provide information about your medical condition.

11. Withdrawal
You can stop at any time and withdraw from the project if you wish. The data collected so far are still evaluated in encrypted form, otherwise the entire project loses its value. It is not possible to anonymize your data in case of withdrawal, i.e. the data remain encrypted. Please check whether you agree with this before you participate in the project.

12. Indemnity
If you participate in this project, you will not receive any compensation. You or your health insurance company will not incur any costs for participation.

13. Liability
The prerequisites and procedure relating to liability and safeguarding in the event of a claim are legally regulated. If you suffer a health impairment as a result of the study, please contact the study doctor. The institution that is responsible for carrying out the study is liable for the claim, if you can prove that the injury is due to the project-specific examinations. Liability will not be accepted if the project manager can prove that the injury is only minor and temporary, and does not extend beyond the degree expected by current scientific knowledge.

14. Funding
The project is being funded by the Swiss National Science Foundation (SNSF).

15. Contact person(s)
If you have any questions, concerns, or emergencies that arise during or after the project, you can always contact one of these contacts.

For peer review only - http://bmjopen.bmj.com/site/about/guidelines.xhtml

Universitätsspital Basel

Head at the study location:
PD Dr. Andreas Müller, Senior Consultant, Head Shoulder and Elbow
University Hospital of Basel, Spitalstrasse 21, CH-403 Basel
Tel 061 315 25 17 , Email A.Mueller@usb.ch

24-hour emergency number: +41 61 265 25 25

Local project coordination:

PD Dr. Andreas Müller, Senior Consultant, Head Shoulder and Elbow
University Hospital of Basel, Spitalstrasse 21, CH-403 Basel
Tel 061 315 25 17 , Email A.Mueller@usb.ch

For peer review only

For peer review only - http://bmjopen.bmj.com/site/about/guidelines.xhtml

Universitätsspital Basel

Declaration of consent

Written declaration of consent for participation in a study project
Please read this form carefully. Please ask if you do not understand or want to know something

**BASEC number (after submission):**	
**Title of the project (scientific and lay)**:	Surgical safety and effectiveness in orthopaedics: Swiss-wide multicenter evaluation and prediction of core outcomes in arthroscopic rotator cuff reconstruction<br><br>Surgical safeguarding and effectiveness in orthopaedics: Swiss-wide multicentre evaluation and prediction of the most important effects following arthroscopic repair of shoulder tendons (rotator cuff reconstruction)
**Responsible institution (Project management with address)**:	University Hospital of Basel<br>PD Dr. Andreas Müller<br>Orthopaedics and Traumatology<br>Spitalstrasse 21, CH-4031 Basel
**Place of implementation**:	Universitätsspital Basel
**Head of the project at the place of study:**	PD Dr. Andreas Müller
**Participant:**	Name, first name: ______________________<br><br>Date of Birth: ______________________<br><br>☐ Female ☐ Male

The undersigned investigator informed me verbally and in writing about the purpose, the course of the project, about possible advantages and disadvantages as well as about possible risks.

- I voluntarily participate in this project and accept the content of the written information provided on the above mentioned project. I've had plenty of time to make my decision.
- My questions concerning the participation in this project have been answered. I keep the written information and receive a copy of my written consent.
- I agree that the responsible experts of the project management/client of the project and the ethics committee responsible for this project may inspect my unencrypted data for verification and control purposes, but in strict compliance with confidentiality.
- I will be informed of study results or random findings that directly affect my health. If I don't want that, I'll inform my investigator.
- I know that my health-related and personal data can only be passed on in encrypted form for research purposes **for this project**.

For peer review only - http://bmjopen.bmj.com/site/about/guidelines.xhtml

Universitätsspital Basel

- In the event of further treatment outside the test centre, I authorise my after-treating doctor(s) to forward my after-treatment data relevant to the project to the investigator/project management.
- I can withdraw from participation at any time and without giving reasons, without having any disadvantages in further medical treatment/care. The data collected so far will still be used for the evaluation of the project.
- The liability insurance of the hospital/institution covers any damages.
- I am aware that the obligations stated in the participant information must be complied with.
- If you agree that your email address can be used solely for receiving questionnaires and project-related communications, please enter it here:

______________________________@______________________

Place, Date	Signature of participant

Confirmation from the investigator: I hereby confirm that I have explained the nature, significance and scope of the project to this participant. I assure you that I will fulfil all obligations in connection with this project in accordance with applicable law. If, at any time during the implementation of the project, I become aware of any aspects that might affect the participant's willingness to participate in the project, I will inform the participant immediately.

Place, Date	Name and first name of the informing investigator in block capitals<br><br>Signature of the investigating physician

For peer review only - http://bmjopen.bmj.com/site/about/guidelines.xhtml

Universitätsspital Basel

Declaration of consent for the further use of data in encrypted form.
(for further use of data of THIS project)

**Participant:**	Name, first name:_______________________<br>Date of birth: __________________________<br>☐ Female ☐ Male

I allow my data from this project to be used in encrypted form for medical research. This means that the data may be stored in a databank and used for future, not yet defined research projects for an indefinite period of time. This consent is unlimited.

I decide voluntarily and can revoke this decision at any time. When I step back, my data is anonymized. I simply inform my investigator/project manager and do not have to justify this decision.

I understand that the data are encrypted and the code is kept safe. The data can be sent to other databanks in Switzerland and abroad for analysis if they comply with the same standards as in Switzerland. All legal requirements regarding data protection are complied with.

Normally, all data are evaluated in their entirety and the results published in summary form. Should a result be relevant for me, it is possible that I will be contacted via my investigator. If I do not wish this, I will inform my investigator/project manager.

If results from the data are handled for commercial purposes, I hereby make no claims on any part of this commercial use.

Place, Date	Signature of participant

Confirmation from the investigator: I hereby confirm that I have explained to this participant the nature, significance and implications of the further use of data.

Place, Date	Name and first name of the informing investigator in block capitals<br><br>Signature of the investigating physician

For peer review only - http://bmjopen.bmj.com/site/about/guidelines.xhtml

Reporting checklist for protocol of a clinical trial.

Based on the SPIRIT guidelines.

Instructions to authors

Complete this checklist by entering the page numbers from your manuscript where readers will find each of the items listed below.

Your article may not currently address all the items on the checklist. Please modify your text to include the missing information. If you are certain that an item does not apply, please write "n/a" and provide a short explanation.

Upload your completed checklist as an extra file when you submit to a journal.

In your methods section, say that you used the SPIRITreporting guidelines, and cite them as:

Chan A-W, Tetzlaff JM, Gøtzsche PC, Altman DG, Mann H, Berlin J, Dickersin K, Hróbjartsson A, Schulz KF, Parulekar WR, Krleža-Jerić K, Laupacis A, Moher D. SPIRIT 2013 Explanation and Elaboration: Guidance for protocols of clinical trials. BMJ. 2013;346:e7586

		Reporting Item	Page Number
**Administrative information**			
Title	#1	Descriptive title identifying the study design, population, interventions, and, if applicable, trial acronym	1
Trial registration	#2a	Trial identifier and registry name. If not yet registered, name of intended registry	4
Trial registration: data set	#2b	All items from the World Health Organization Trial Registration Data Set	n.a.
Protocol version	#3	Date and version identifier	4
Funding	#4	Sources and types of financial, material, and other support	4
Roles and responsibilities: contributorship	#5a	Names, affiliations, and roles of protocol contributors	4

For peer review only

Roles and responsibilities: sponsor contact information	#5b	Name and contact information for the trial sponsor	4
Roles and responsibilities: sponsor and funder	#5c	Role of study sponsor and funders, if any, in study design; collection, management, analysis, and interpretation of data; writing of the report; and the decision to submit the report for publication, including whether they will have ultimate authority over any of these activities	4
Roles and responsibilities: committees	#5d	Composition, roles, and responsibilities of the coordinating centre, steering committee, endpoint adjudication committee, data management team, and other individuals or groups overseeing the trial, if applicable (see Item 21a for data monitoring committee)	4
**Introduction**			
Background and rationale	#6a	Description of research question and justification for undertaking the trial, including summary of relevant studies (published and unpublished) examining benefits and harms for each intervention	7
Background and rationale: choice of comparators	#6b	Explanation for choice of comparators	n.a.
Objectives	#7	Specific objectives or hypotheses	9
Trial design	#8	Description of trial design including type of trial (eg, parallel group, crossover, factorial, single group), allocation ratio, and framework (eg, superiority, equivalence, non-inferiority, exploratory)	9
**Methods: Participants, interventions, and outcomes**			
Study setting	#9	Description of study settings (eg, community clinic,	9

For peer review only

		academic hospital) and list of countries where data will be collected. Reference to where list of study sites can be obtained	
Eligibility criteria	#10	Inclusion and exclusion criteria for participants. If applicable, eligibility criteria for study centres and individuals who will perform the interventions (eg, surgeons, psychotherapists)	10
Interventions: description	#11a	Interventions for each group with sufficient detail to allow replication, including how and when they will be administered	10
Interventions: modifications	#11b	Criteria for discontinuing or modifying allocated interventions for a given trial participant (eg, drug dose change in response to harms, participant request, or improving / worsening disease)	n.a.
Interventions: adherance	#11c	Strategies to improve adherence to intervention protocols, and any procedures for monitoring adherence (eg, drug tablet return; laboratory tests)	n.a.
Interventions: concomitant care	#11d	Relevant concomitant care and interventions that are permitted or prohibited during the trial	10
Outcomes	#12	Primary, secondary, and other outcomes, including the specific measurement variable (eg, systolic blood pressure), analysis metric (eg, change from baseline, final value, time to event), method of aggregation (eg, median, proportion), and time point for each outcome. Explanation of the clinical relevance of chosen efficacy and harm outcomes is strongly recommended	11
Participant timeline	#13	Time schedule of enrolment, interventions (including any run-ins and washouts), assessments, and visits for participants. A schematic diagram is highly recommended (see Figure)	13
Sample size	#14	Estimated number of participants needed to achieve study objectives and how it was determined, including clinical and statistical assumptions supporting any sample size	15

For peer review only

		calculations	
Recruitment	#15	Strategies for achieving adequate participant enrolment to reach target sample size	16
**Methods: Assignment of interventions (for controlled trials)**			
Allocation: sequence generation	#16a	Method of generating the allocation sequence (eg, computer-generated random numbers), and list of any factors for stratification. To reduce predictability of a random sequence, details of any planned restriction (eg, blocking) should be provided in a separate document that is unavailable to those who enrol participants or assign interventions	n.a.
Allocation concealment mechanism	#16b	Mechanism of implementing the allocation sequence (eg, central telephone; sequentially numbered, opaque, sealed envelopes), describing any steps to conceal the sequence until interventions are assigned	n.a.
Allocation: implementation	#16c	Who will generate the allocation sequence, who will enrol participants, and who will assign participants to interventions	n.a.
Blinding (masking)	#17a	Who will be blinded after assignment to interventions (eg, trial participants, care providers, outcome assessors, data analysts), and how	n.a.
Blinding (masking): emergency unblinding	#17b	If blinded, circumstances under which unblinding is permissible, and procedure for revealing a participant's allocated intervention during the trial	n.a.
**Methods: Data collection, management, and analysis**			
Data collection plan	#18a	Plans for assessment and collection of outcome, baseline, and other trial data, including any related processes to promote data quality (eg, duplicate	16

For peer review only

		measurements, training of assessors) and a description of study instruments (eg, questionnaires, laboratory tests) along with their reliability and validity, if known. Reference to where data collection forms can be found, if not in the protocol	
Data collection plan: retention	#18b	Plans to promote participant retention and complete follow-up, including list of any outcome data to be collected for participants who discontinue or deviate from intervention protocols	16
Data management	#19	Plans for data entry, coding, security, and storage, including any related processes to promote data quality (eg, double data entry; range checks for data values). Reference to where details of data management procedures can be found, if not in the protocol	18
Statistics: outcomes	#20a	Statistical methods for analysing primary and secondary outcomes. Reference to where other details of the statistical analysis plan can be found, if not in the protocol	19
Statistics: additional analyses	#20b	Methods for any additional analyses (eg, subgroup and adjusted analyses)	20
Statistics: analysis population and missing data	#20c	Definition of analysis population relating to protocol non-adherence (eg, as randomised analysis), and any statistical methods to handle missing data (eg, multiple imputation)	20
**Methods: Monitoring**			
Data monitoring: formal committee	#21a	Composition of data monitoring committee (DMC); summary of its role and reporting structure; statement of whether it is independent from the sponsor and competing interests; and reference to where further details about its charter can be found, if not in the protocol. Alternatively, an explanation of why a DMC is not needed	4<br>project scientific board (PSB)

For peer review only

Data monitoring: interim analysis	#21b	Description of any interim analyses and stopping guidelines, including who will have access to these interim results and make the final decision to terminate the trial	n.a.
Harms	#22	Plans for collecting, assessing, reporting, and managing solicited and spontaneously reported adverse events and other unintended effects of trial interventions or trial conduct	14
Auditing	#23	Frequency and procedures for auditing trial conduct, if any, and whether the process will be independent from investigators and the sponsor	18
**Ethics and dissemination**			
Research ethics approval	#24	Plans for seeking research ethics committee / institutional review board (REC / IRB) approval	21
Protocol amendments	#25	Plans for communicating important protocol modifications (eg, changes to eligibility criteria, outcomes, analyses) to relevant parties (eg, investigators, REC / IRBs, trial participants, trial registries, journals, regulators)	21
Consent or assent	#26a	Who will obtain informed consent or assent from potential trial participants or authorised surrogates, and how (see Item 32)	21
Consent or assent: ancillary studies	#26b	Additional consent provisions for collection and use of participant data and biological specimens in ancillary studies, if applicable	n.a.
Confidentiality	#27	How personal information about potential and enrolled participants will be collected, shared, and maintained in order to protect confidentiality before, during, and after the trial	22
Declaration of interests	#28	Financial and other competing interests for principal investigators for the overall trial and each study site	22
Data access	#29	Statement of who will have access to the final trial dataset, and disclosure of contractual agreements	22

For peer review only

		that limit such access for investigators	
Ancillary and post trial care	#30	Provisions, if any, for ancillary and post-trial care, and for compensation to those who suffer harm from trial participation	n.a.
Dissemination policy: trial results	#31a	Plans for investigators and sponsor to communicate trial results to participants, healthcare professionals, the public, and other relevant groups (eg, via publication, reporting in results databases, or other data sharing arrangements), including any publication restrictions	23
Dissemination policy: authorship	#31b	Authorship eligibility guidelines and any intended use of professional writers	23
Dissemination policy: reproducible research	#31c	Plans, if any, for granting public access to the full protocol, participant-level dataset, and statistical code	22
**Appendices**			
Informed consent materials	#32	Model consent form and other related documentation given to participants and authorised surrogates	Supplementary file
Biological specimens	#33	Plans for collection, laboratory evaluation, and storage of biological specimens for genetic or molecular analysis in the current trial and for future use in ancillary studies, if applicable	n.a.

None The SPIRIT checklist is distributed under the terms of the Creative Commons Attribution License CC-BY-ND 3.0. This checklist can be completed online using https://www.goodreports.org/, a tool made by the EQUATOR Network in collaboration with Penelope.ai

For peer review only
